# The deacetylases HDAC1/HDAC2 control JAK2^V617F^-STAT signaling through the ubiquitin ligase SIAH2

**DOI:** 10.1038/s41392-025-02369-7

**Published:** 2025-08-29

**Authors:** Al-Hassan M. Mustafa, Giuseppe Petrosino, Marten A. Fischer, Tina M. Schnöder, Désirée Gül, Yanira Zeyn, Christoph Hieber, Johanna Lossa, Sabine Muth, Markus P. Radsak, Walburgis Brenner, Markus Christmann, Matthias Bros, Florian H. Heidel, Oliver H. Krämer

**Affiliations:** 1 Institute of Toxicology, University Medical Center of Johannes Gutenberg University, Mainz, Germany; 2 Department of Zoology, Faculty of Science, Aswan University, Aswan, Egypt; 3 Institute of Molecular Biology (IMB), Mainz, Germany; 4 Hematology, Hemostasis, Oncology and Stem Cell Transplantation, Hannover Medical School (MHH), Hannover, Germany; 5 Department of Otorhinolaryngology Head and Neck Surgery; Molecular and Cellular Oncology, University Medical Center of Johannes Gutenberg University, Mainz, Germany; 6 Department of Dermatology, University Medical Center of Johannes Gutenberg University, Mainz, Germany; 7 Institute of Immunology, University Medical Center of the Johannes Gutenberg University, Mainz, Germany; 8 Department of Hematology and Medical Oncology, University Medical Center of Johannes Gutenberg University, Mainz, Germany; 9 Department of Obstetrics and Gynecology, University Medical Center of Johannes Gutenberg University, Mainz, Germany; 10 Leibniz Institute on Aging, Fritz-Lipmann-Institute, Jena, Germany

**Keywords:** Haematological cancer, Oncogenes, Target identification, Haematopoietic stem cells

## Abstract

Epigenetic modulators of the histone deacetylase (HDAC) family control key biological processes and are frequently dysregulated in cancer. There is superior activity of HDAC inhibitors (HDACi) in patients with myeloproliferative neoplasms (MPNs) that carry the Janus kinase-2 point mutant JAK2^V617F^. This constitutively active tyrosine kinase activates signal-transducer-and-activator-of-transcription (STAT) transcription factors to promote cell proliferation and inflammatory processes. We reveal that the inhibition of HDAC1/HDAC2 with the clinically advanced HDACi romidepsin, the experimental HDACi entinostat and MERCK60, and genetic depletion of HDAC1/HDAC2 induce apoptosis and long-term growth arrest of primary and permanent MPN cells in vitro and in vivo. This treatment spares normal hematopoietic stem cells and does not compromise blood cell differentiation. At the molecular level, HDAC1 and HDAC2 control the protein stability of SIAH2 through acetylation. Genetic knockout experiments show that SIAH2 accelerates the proteasomal degradation of JAK2^V617F^ in conjunction with the E2 ubiquitin-conjugating enzyme UBCH8. SIAH2 binds to the surface-exposed SIAH degron motif VLP1002 in the catalytic domain of JAK2^V617F^. At the functional level, SIAH2 knockout MPN cells are significantly less sensitive to HDACi. Global RNA sequencing verifies that JAK-STAT signaling is a prime target of SIAH2. Moreover, HDAC1 is an adverse prognostic factor in patients with acute myeloid leukemia (*n* = 150, *p* = 0.02), being a possible complication of MPNs. These insights reveal a previously unappreciated link between HDAC1/HDAC2 as key molecular targets, the still undefined regulation of cytoplasmic-to-nuclear signaling by HDACs, and how HDACi kill JAK2^V617F^-positive cells from MPN patients and mice with JAK2^V617F^ in vitro and in vivo.

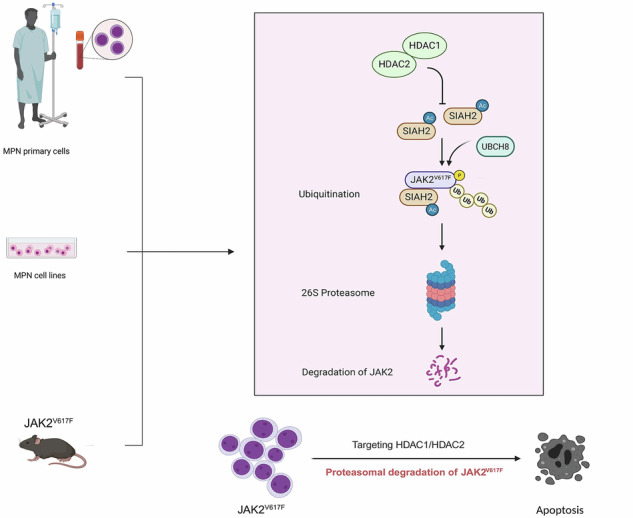

## Introduction

The Janus kinase (JAK) signaling pathway exemplifies the hierarchical organization of cell signaling, enables receptor signaling across class I cytokines and thereby centrally orchestrates integral cellular functions through a network of major signaling nodes. JAKs transduce signals across various cytokines, hormones, and growth factor receptors in hematopoietic cells.^1–3^

Constitutive activation of JAK2 signaling results from its most prevalent mutation at amino acid residue 617 (JAK2^V617F^). This genetic aberration initiates signaling networks that are detectable in most patients with myeloproliferative neoplasms (MPNs). Patients with polycythemia vera (PV) and more than half of those with essential thrombocythemia (ET) and primary myelofibrosis (PMF) carry JAK2^V617F^. Studying JAK2^V617F^ as an early somatic event in chronic myeloid malignancies allows a deeper understanding of initial tumorigenesis.^4^ The constitutive tyrosine kinase activity of JAK2^V617F^ induces signal-transducer-and-activator-of-transcription (STAT) transcription factors to promote MPN cell proliferation and survival as well as MPN-associated inflammatory ailments.^1–3^ High-resolution phospho-proteome analyses allowed for the first time a global visualization of signal transduction dependent on the constitutively active JAK2 kinase.^5^ The inflammatory phenotype observed in MPN patients is a major aspect of JAK2-induced pathophysiology and contributes to morbidity and mortality. JAK inhibitors have shown significant clinical activity in reducing inflammation.^1,6^ Whereas in other cancers, inactivation of oncogenic kinases or drivers results in rapid and durable regression of the malignant clone, pharmacologic inhibition of JAK2 neither significantly affects the overall disease burden nor inhibits the evolution of disease persistence to a major extent.^7,8^ Current preclinical developments strive to prevent disease progression, reduce disease burden, and improve cytopenia.

Despite such limitations of currently available JAK inhibitors, the genetic elimination of JAK2^V617F^ in MPN cells reversed disease and extended the overall survival of mice through the depletion of mutant hematopoietic stem and progenitor cells.^9^ Thus, elimination of JAK2^V617F^ has emerged as a valid strategy in MPNs. A pharmacologically amenable, indirect route to target JAK2^V617F^ relies on epigenetic modulators of the histone deacetylase (HDAC) family.^2^ These enzymes use Zn^2+^ (HDAC classes I, II, IV) or NAD^+^ (HDAC class III) to deacetylate lysine residues within proteins. Class I HDACs include HDAC1, HDAC2, HDAC3, and HDAC8; class IIa HDACs include HDAC4, HDAC5, HDAC7, and HDAC9; class IIb HDACs include HDAC6 and HDAC10; class III HDACs include the sirtuins SIRT1-7; and HDAC11 is the sole member of class IV HDACs. Research has shown that HDACs can be detected in both the nucleus and the cytoplasm.^2^ Even class I HDACs, which are the founding HDAC members and persuasive regulators of transcription by histone deacetylation, have functionally significant cytoplasmic targets. This can be explained by all proteins being produced in the cytosol and the shuttling of low, previously undetected levels of HDACs between compartments.^2,10,11^ Since HDACs also deacetylate nonhistone proteins, attempts have been made to rename them protein deacetylases (PDACs).^2,11^ To maintain consistency with most of the literature on HDACs, the term HDAC is commonly used to describe these enzymes.

Food and drug administrations have approved the Zn^2+^-dependent HDAC inhibitors (HDACi) vorinostat, romidepsin (FK228), belinostat, and tucidinostat for the treatment of patients with peripheral/cutaneous lymphoma and myeloma. JAK2^V617F^-positive MPN patients have been treated with HDACi (www.clinicaltrials.org), resulting in detectable rates of clinical success in such patients.^2,12^ These observations warrant the identification of molecular mechanisms and markers that underlie the sensitivity of JAK2^V617F^-positive MPNs to HDACi. The clinically promising class I/II hydroxamic acid-derived HDACi givinostat depletes JAK2^V617F^ by incompletely defined molecular mechanisms.^13^ Vorinostat and panobinostat are additional hydroxamic acid-based HDACi that eliminate JAK2^V617F^-positive cells in vitro, in mice, and in patients with PV and ET by unresolved molecular pathways.^2,14,15^ Defining such mechanisms can deliver new strategies to treat JAK2^V617F^-positive ailments. A possible mechanism through which HDAC6 regulates HSP90 acetylation and its association with JAK2^V617F^ was questioned. HDAC11 stabilizes phosphorylated JAK2^V617F^ without affecting total JAK2^V617F^ levels.^16^ Thus, additional studies are needed to explain how HDACi attenuate JAK2^V617F^ and which individual HDAC(s) maintain the stability of JAK2^V617F^. Such insights allow the clinical use of highly specific HDACi, which have less adverse effects than broad-acting agents.^2,12^

HDACi modulate the ubiquitin‒proteasome system, which mediates the turnover of more than 80% of cellular proteins.^17,18^ E1, E2, and E3 enzymes catalyze protein ubiquitination. Mammalian seven-in-absentia homologs (SIAHs) are highly active E3 ubiquitin ligases. Human SIAH1 (∼32 kDa) and SIAH2 (∼37 kDa) are present in various cell compartments and accelerate the proteasomal degradation of nuclear and cytoplasmic leukemia-promoting proteins. These include mutant transcription factors and kinases.^17,19^ Remarkably, SIAH2 knockout mice exhibit expansion of myeloid progenitor cells in their bone marrow.^20^ Whether SIAHs affect wild-type and mutant JAK2 is unclear. Further work is needed to clarify whether HDACi increase the acetylation of SIAH2, which has yet only been reported in gastric cancer cells,^21,22^ and if this posttranslational modification controls oncoprotein stability. It is likewise unclear whether the E2 ubiquitin-conjugating enzyme UBCH8, which can deplete oncoproteins in cooperation with SIAH2,^17^ has an effect on JAK2^V617F^ and the fate of MPN cells.

This work reveals that inhibition of HDAC1 and HDAC2 induces the proteasomal degradation of JAK2^V617F^ through an acetylation-dependent stabilization of SIAH2, a defined SIAH2 binding motif in JAK2^V617F^, and a mechanism that requires UBCH8. We further demonstrate that this molecular mechanism determines the survival of primary and permanent MPN cells in vitro and in vivo and that this signal transduction pathway is a targetable vulnerability of MPN cells.

## Results

### Class I HDACs control the survival of MPN cells that are addicted to hyperactive JAK-STAT signaling

**Fig. 1:**
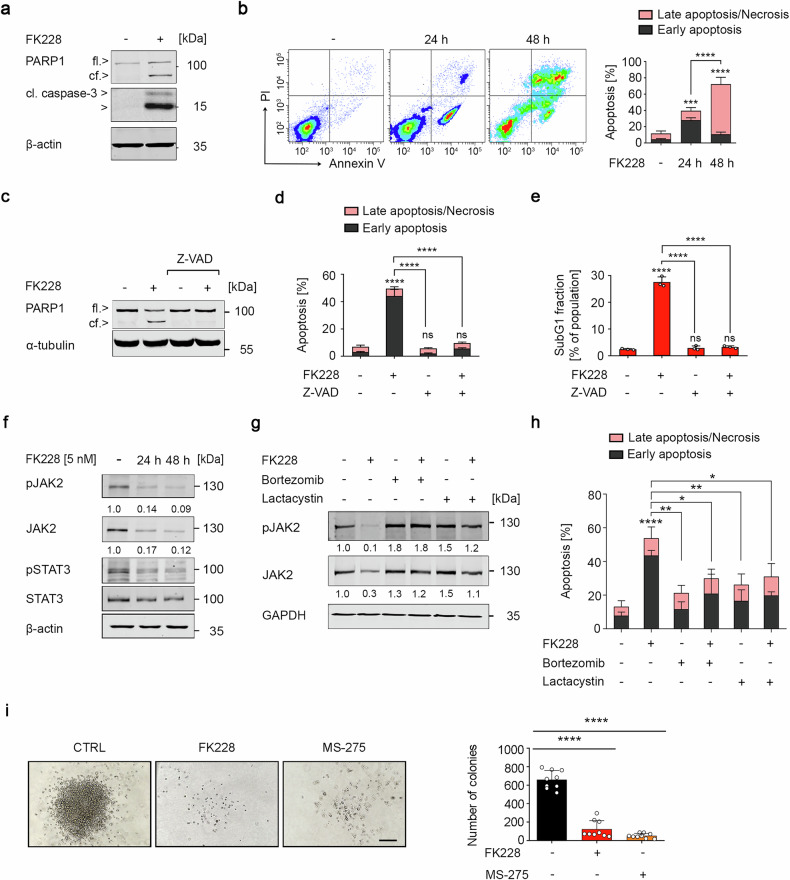
Inhibition of class I HDACs induces apoptosis in JAK2^V617F^-positive cells. **a** HEL cells were treated with 5 nM FK228 for 24 h (-, untreated control cells; +, treated cells). Immunoblotting shows full-length (fl.) and cleaved (cf.) PARP1 and cleaved forms of caspase-3; β-actin was used as a loading control; kDa, kilo Daltons. **b** HEL cells were treated with 5 nM FK228 for 24–48 h, stained with annexin-V-FITC/PI, and analyzed for early (annexin-V-positive) and late (annexin-V/PI-positive) apoptosis by flow cytometry. Left shows representative flow cytometry scans; right shows bar graphs with statistical evaluations. **c** HEL cells were incubated with 5 nM FK228 and/or 50 μM z-VAD-FMK for 24 h. PARP1 was detected by immunoblotting; α-tubulin served as a loading control. **d** HEL cells were treated as described in (**c**) and subsequently stained with annexin-V-FITC/PI and analyzed for apoptosis. **e** HEL cells were treated as described in (**c**), fixed, subsequently stained with PI, and analyzed for subG1 fraction accumulation via flow cytometry. **f** HEL cells were treated with 5 nM FK228 for 24–48 h. The indicated proteins were revealed by immunoblotting; β-actin was used as a loading control. **g** HEL cells were treated with 5 nM FK228 for 24 h and/or proteasome inhibitors (50 nM bortezomib; 10 μM lactacystin) for 6 h before being harvested. Immunoblotting shows the indicated proteins. **h** HEL cells were treated as described in (**g**) and analyzed for apoptosis via flow cytometry. **i** HEL cells were incubated with 5 nM FK228 or 5 µM MS-275 for 24 h, washed, plated into methylcellulose medium, and tested for colony formation. Left, representative images are shown. Scale bar, 200 μm. Right, graph depicts the numbers of colonies. The data are presented as mean ± SD of at least three independent experiments. Statistical analyses (unpaired *t*-test; one-way ANOVA; two-way ANOVA; Bonferroni correction; ns not significant; *p* values are as follows: *P  < 0.05; **P  < 0.01; ***P  < 0.001; ****P  < 0.0001)

Since class I HDACs are key regulators of tumorigenesis,^2^ we hypothesized that MPN cells require these HDACs for their growth and survival. To evaluate this, we incubated the JAK2^V617F^-transformed male and female human MPN cell systems HEL, SET-2, and UKE-1 with the HDACi FK228 for 24 h. This agent inhibits class I HDACs.^2^ FK228 induced proteolysis-dependent activation of the ultimate apoptosis executioner enzyme caspase-3 and cleavage of its substrate PARP1 in all three MPN cell systems (Fig. 1a and Supplementary Fig. 1a–c). These proapoptotic processes increased progressively with increasing duration of FK228 incubation (Supplementary Fig. 1a–c). Analysis of apoptosis resulting from exposure of phosphatidylserine residues on the cell surface and DNA fragmentation via flow cytometry in these three cell types disclosed that 5 nM FK228 induced 40–80% apoptosis after 24–48 h and that the impact of FK228 on these cells was time-dependent (Fig. 1b and Supplementary Fig. 1d‒l). We confirmed the induction of apoptosis by FK228 with the pancaspase inhibitor z-VAD-FMK. This agent blunted the cleavage of PARP1, accumulation of annexin-V/PI-positive cells, and increase in subG1 phase fractions in response to FK228 (Fig. 1c–e).

We aimed to substantiate these data with HDACi that are selective for the class I HDACs HDAC1, HDAC2, HDAC3, or HDAC8. Like FK228, the benzamide MS-275, which inhibits HDAC1, HDAC2, and HDAC3,^2^ significantly induced apoptosis in JAK2^V617F^-expressing cells. MS-275 induced the accumulation of annexin-V/PI-positive cells and the activation of caspase-3 in all tested MPN cell systems (Supplementary Fig. 2a–d).

In contrast, the selective HDAC8 inhibitor PCI-34051 did not induce apoptosis in HEL, SET2, or UKE-1 cells (Supplementary Fig. 2b–d). We ensured the activity of PCI-34051 as hyperacetylation of the HDAC8 target SMC3 (Supplementary Fig. 2e). Since there is a dispute regarding whether MPN cells depend on the class IIb deacetylase HDAC6,^16^ we treated HEL and SET-2 cells with the HDAC6 inhibitor marbostat-100.^23^ Marbostat-100 induced hyperacetylation of the HDAC6 target tubulin but not apoptosis (Supplementary Fig. 2f–i).

Given that patients with JAK2^V617F^-positive MPN frequently benefit from HDACi,^2,12^ we evaluated whether the expression of JAK2^V617F^ can be translated into a cellular susceptibility to HDACi. JAK2^V617F^ allows cytokine-independent growth of the murine myeloblast-like 32D cells. The corresponding 32D cells with JAK2 resemble normal cells, as they require the cytokine IL-3 for survival. We exposed 32D cells expressing JAK2 or JAK2^V617F^ to 5 nM FK228 and probed them for annexin-V/PI and apoptosis-associated DNA fragmentation. The 32D cells harboring JAK2^V617F^ were significantly more sensitive to FK228 than the cognate 32D cells with JAK2 (Supplementary Fig. 2j, k).

Since MPN cells are addicted to JAK2^V617F^-STAT signaling, the loss of this signaling pathway may explain HDACi-induced apoptosis. We investigated whether class I HDACi affected JAK2^V617F^ and its activating phosphorylation. FK228 and MS-275 reduced phosphorylated and total JAK2^V617F^ in various MPN cell systems in dose- and time-dependent manner (Fig. 1f and Supplementary Fig. 3a–i). Consequently, FK228 decreased the levels of phosphorylated STAT1, STAT1, phosphorylated STAT3, STAT3, phosphorylated STAT5, and STAT5 in HEL, SET-2, and UKE-1 cells (Fig. 1f and Supplementary Fig. 3a–i).

PCI-34051 had no effect on JAK2^V617F^ levels in HEL, SET2, or UKE-1 cells (Supplementary Fig. 3j). This corresponds to the lack of apoptosis induction by this agent in such JAK2^V617F^-transformed cells (Supplementary Fig. 2b–d).

We asked whether class I HDACi attenuated the levels of wild-type JAK2. The application of 5 to 25 nM FK228 for 24 h reduced JAK2 in K562 cells, which belong to the CML cell type within the group of MPNs (Supplementary Fig. 3k). Likewise, the treatment of normal human peripheral blood mononuclear cells (PBMCs) with 5 nM FK228 for 24–48 h decreased JAK2 levels as well as phosphorylated and total STAT3 (Supplementary Fig. 3l). Analyses in the DepMap database illustrate that the elimination of JAK2 does not significantly compromise the proliferation of leukemia cells harboring wild-type JAK2 (Supplementary Fig. 3m). These findings suggest JAK2-independent proliferation and survival of leukemia cells with wild-type JAK2.

Next, we aimed to delineate the molecular mechanism by which HDACi deplete JAK2^V617F^. We investigated whether HDACi eliminated JAK2^V617F^ by caspases, being the main executioners of apoptosis. Although the pancaspase inhibitor z-VAD-FMK suppressed apoptosis induction by FK228 (Fig. 1c–e), it could not restore JAK2^V617F^ (Supplementary Fig. 3n). The identification of the proteasomal degradation of oncoproteins in HDACi-treated leukemic cells^2,17^ suggested similar mechanisms for JAK2^V617F^. The structurally different proteasome inhibitors bortezomib and lactacystin prevented the FK228-induced depletion of phosphorylated and total JAK2^V617F^ and significantly attenuated apoptosis in FK228-exposed HEL and SET-2 cells (Fig. 1g, h and Supplementary Fig. 4a, b). Thus, our finding that HDACi reduce the expression of *JAK2* mRNA cannot explain the decrease in JAK2^V617F^ in transformed cells with mutant or wild-type JAK2 (Supplementary Fig. 4c, d).

We asked if class I HDACi had a lasting effect on leukemic cell growth using a colony formation assay in the semi-solid matrix methylcellulose. This resembles cellular growth conditions within the bone marrow. HEL cells lost their colony-founding potential significantly after a single exposure to 5 nM FK228 or 5 µM MS-275 (Fig. 1i).

These insights show that class I HDACi induce apoptosis and long-term growth arrest in cells with JAK2^V617F^ and that these beneficial processes are linked to accelerated proteasomal degradation of JAK2^V617F^.

### HDAC1 and HDAC2 maintain the survival of MPN cells

**Fig. 2:**
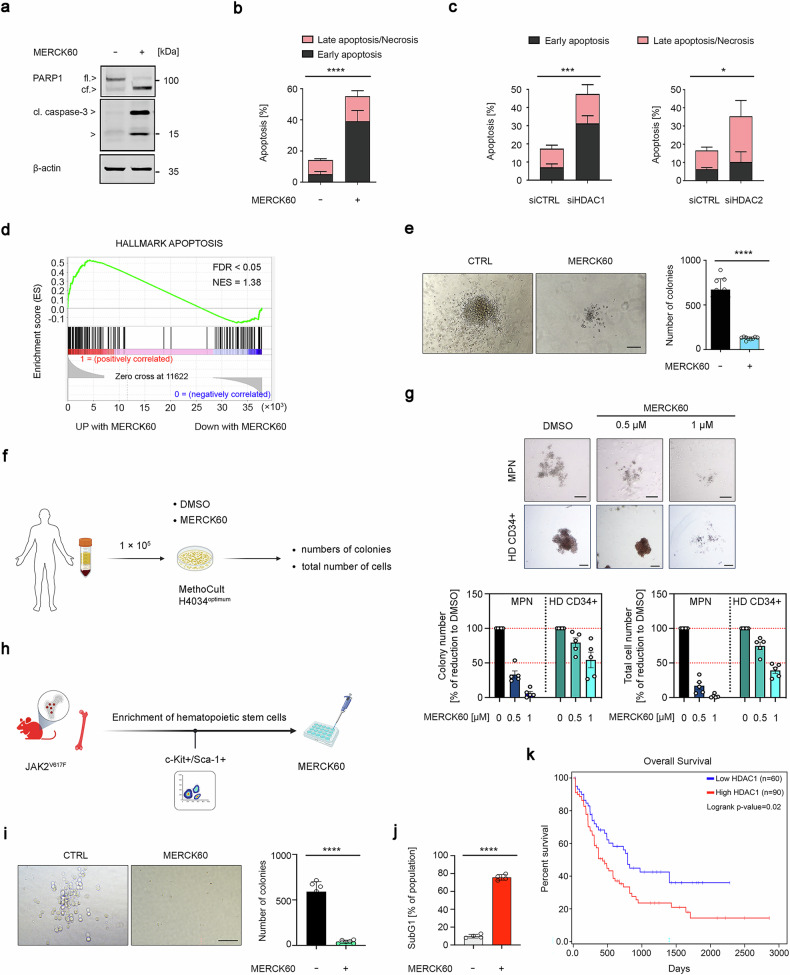
HDAC1 and HDAC2 protect JAK2^V617F^-positive cells from apoptosis. **a** HEL cells were incubated with 5 μM MERCK60 for 24 h. PARP1 and caspase-3 were detected by immunoblotting; β-actin, loading control. **b** HEL cells were treated as described in (**a**), stained with annexin-V-FITC/PI, and analyzed for apoptosis. **c** HEL cells were transfected with siRNAs against HDAC1 or HDAC2 for 48 h. The cells were stained with annexin-V-FITC/PI and analyzed for apoptosis. **d** GSEA analyses revealed the upregulation of proapoptotic gene signatures in HEL cells that were treated with 5 μM MERCK60 for 24 h, with a normalized enrichment score (NES) of 1.38 and an FDR *q* value < 0.05. **e** HEL cells were treated with 5 μM MERCK60 for 48 h, washed, plated in methylcellulose medium, and analyzed for colony formation after 14 days. Left, representative images are shown. Scale bar, 200 μm. Right, graph depicts the numbers of colonies. **f** Scheme depicting the experiment for which (**g**) shows the outcome; created with BioRender (https://BioRender.com). **g** MPN patient-derived JAK2-mutated PBMCs and HSPCs from healthy donors (HD) were treated with 0.5 or 1 μM MERCK60 or with DMSO as a control and plated in methylcellulose. The number of colonies and total number of cells were counted on day 10. Representative images are shown (upper panel). Scale bar, 200 μm. **h** Scheme depicting the experiment for which (**i**, **j**) show the outcome; created with BioRender (https://BioRender.com). **i**, **j** Hematopoietic stem and progenitor cells were isolated from the bone marrow of three mice with PV. Leukemic stem cells were enriched and tested for c-Kit and Sca-1 expression. The cells were treated with 5 µM MERCK60 for 24 h, washed, plated in methylcellulose, and analyzed for colony formation after 10 days; scale bar, 100 μm (**i**) or fixed, subsequently stained with PI, and analyzed for subG1 fraction accumulation via flow cytometry (**j**). **k** TCGA dataset analysis revealed that the overall survival of AML patients inversely correlates with the expression of *HDAC1* mRNA. The data are presented as mean ± SD of three independent experiments. Statistical analyses (unpaired *t*-test; one-way ANOVA; two-way ANOVA; Bonferroni correction; ns not significant; *P  < 0.05; **P  < 0.01; ***P  < 0.001; ****P  < 0.0001)

Above, we disclose that MS-275, which blocks HDAC1, HDAC2, and HDAC3, depletes JAK2^V617F^ and induces apoptosis in MPN cells (Supplementary Fig. 2a–d and Supplementary Fig. 3h, i). MERCK60 is a benzamide derivative that selectively inhibits HDAC1/HDAC2.^24^ The application of 5 µM MERCK60 for 48 h activated caspase-3, induced the cleavage of PARP1, and significantly augmented the number of annexin-V/PI-positive HEL and UKE-1 cells (Fig. 2a, b and Supplementary Fig. 5a, b). We determined the IC_50_ growth inhibitory values for MERCK60 as 1039 nM and 1461 nM in these MPN cells (Supplementary Fig. 5c).

To ensure that HDAC1 and HDAC2 ensure MPN cell survival, we silenced them with validated sequence-specific siRNAs. Depletion of HDAC1 or HDAC2 significantly induced apoptosis in HEL cell cultures (Fig. 2c and Supplementary Fig. 5d). A combined reduction in HDAC1 and HDAC2 was as effective as single knockdown of HDAC1 and HDAC2 (Supplementary Fig. 5e, f), indicating that such cells require both HDACs to prevent programmed cell death through apoptosis. Hence, MERCK60 is a valid compound for analyzing how HDAC1 and HDAC2 control the fate of our cell systems.

This notion encouraged further tests with MERCK60. Profiling the transcriptomes of untreated and MERCK60-treated HEL cells via RNA sequencing confirmed these data. Gene set enrichment analysis (GSEA) disclosed that MERCK60 upregulated proapoptotic genes significantly (Fig. 2d). Coherent with this, MERCK60 abolished the survival and proliferation of HEL cells significantly (Fig. 2e).

We consequently assessed how MERCK60 affected the fate of primary MPN patient cells harboring JAK2^V617F^ and normal hematopoietic stem and progenitor cells (HSPCs) (Fig. 2f). We found that the application of 0.5 to 1 µM MERCK60 sufficed to significantly reduce colony formation in cells from six different JAK2-mutated MPN patients in vitro compared with that in HSPCs from healthy donor controls (Fig. 2g). We obtained similar data with Sca-1^+^/c-Kit^+^ primary hematopoietic stem progenitor cells from JAK2^V617F^-knock-in mice (Fig. 2h). MERCK60 abrogated the survival and proliferative capacity of primary Sca-1^+^/c-Kit^+^ JAK2^V617F^-positive murine hematopoietic stem cells (Fig. 2i). This was associated with a significant induction of apoptotic DNA fragmentation (Fig. 2j).

Leukemic transformation occurs in 1%, 4%, and 20% of patients over a 10-year period in ET, PV, and PMF, respectively.^25^ When we analyzed data from 150 AML patients from the TCGA database, we found that patients with higher HDAC1 expression had poorer survival rates (Fig. 2k). We retrieved similar survival data from the GEPIA2 database. In 54 AML patients, higher expression levels of HDAC1 were significantly associated with worse prognosis (Supplementary Fig. 5g).

These data indicate that HDAC1 and HDAC2 are necessary for the survival of leukemic cells harboring JAK2^V617F^.

### HDAC1 and HDAC2 control the stability of SIAH2 as E3 ubiquitin ligase for JAK2^V617F^

**Fig. 3:**
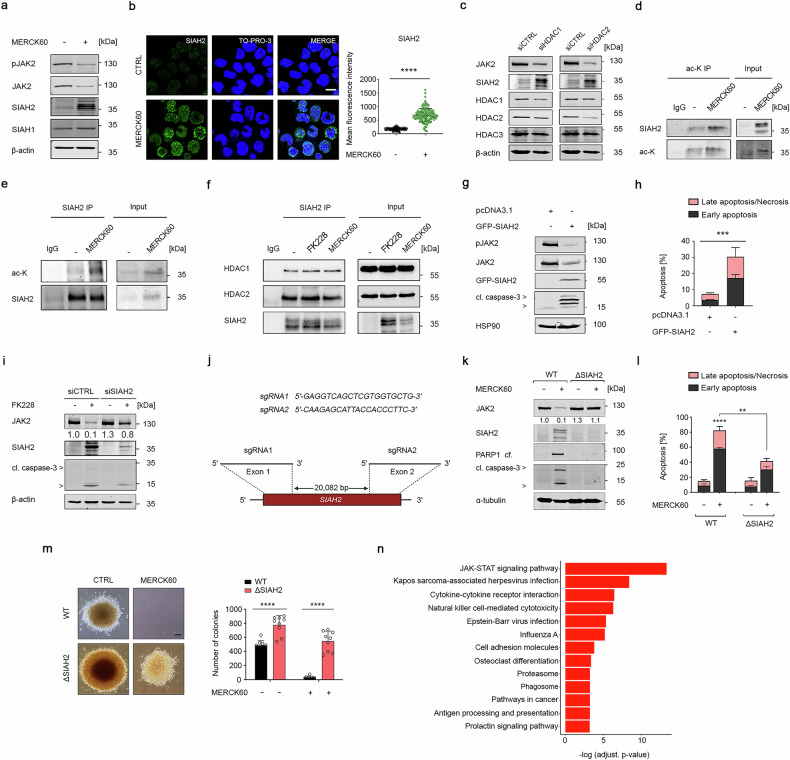
Targeting HDAC1 and HDAC2 induces SIAH2-mediated degradation of JAK2^V617F^ signaling. **a** HEL cells were incubated with 5 μM MERCK60 for 24 h. The cells were lysed, and the indicated proteins were analyzed by immunoblotting; β-actin served as a loading control. **b** HEL cells were treated with MERCK60 as described in (**a**), fixed, and stained for SIAH2. TO-PRO-3 was used to visualize the nuclei. The cells were examined via confocal laser scanning microscopy. Representative images are shown; *n* = 3; scale bar, 10 µm (left panel). The mean fluorescence intensities were measured with ImageJ software (right panel). **c** HEL cells were transfected with siRNAs against HDAC1 or HDAC2 for 48 h. Immunoblotting was used to verify the reduction in HDACs and the specificity of the siRNAs. Levels of JAK2^V617F^ and SIAH2 were detected by immunoblotting; β-actin served as a loading control. **d** HEL cells were treated with 5 μM MERCK60 for 48 h. Immunoprecipitates with anti-pan-acetylated lysine (ac-K) antibody or rabbit preimmune serum (IgG) were analyzed for SIAH2 by immunoblotting. **e** K562 cells were treated as described in (**d**), and immunoprecipitates with an anti-SIAH2 antibody or mouse preimmune serum (IgG) were analyzed for pan-acetylated lysine (ac-K) by immunoblotting. **f** HEL cells were treated with 5 nM FK228 or 5 μM MERCK60 for 48 h. Immunoprecipitates with anti-SIAH2 antibody or mouse preimmune serum (IgG) were analyzed for interaction with HDAC1 and HDAC2 by immunoblotting. **g** HEL cells were transfected with pcDNA3.1 or pcDNA3.1-GFP-SIAH2 plasmids for 48 h. Immunoblotting was used to verify the expression of JAK2^V617F^, GFP-SIAH2, and cleaved caspase-3; HSP90, loading control. **h** HEL cells were transfected as described in (**g**), stained with annexin-V-FITC/PI and analyzed for apoptosis via flow cytometry. **i** HEL cells were transfected with siRNAs against SIAH2 for 48 h. Immunoblotting was used to verify the depletion of SIAH2. The levels of JAK2^V617F^ and cleaved caspase-3 were detected by immunoblotting; β-actin served as a loading control. **j** Exon 1 and exon 2 of the human *SIAH2* gene were disrupted by two gRNAs. **k** Wild-type (HEL^WT^) and SIAH2 knockout HEL cells (HEL^ΔSIAH2^) were incubated with 5 µM MERCK60 for 48 h. Immunoblotting was used to verify the indicated proteins. α-tubulin served as a loading control. **l** Aliquots of cells from the experiments mentioned in (**k**) were stained with annexin-V-FITC/PI and analyzed for apoptosis. **m** HEL^WT^ and HEL^ΔSIAH2^ were treated as described in (**k**), washed, and analyzed for colony formation after 14 days. Left, representative images are shown. Scale bar, 100 μm. Right, graph depicts the numbers of colonies. **n** KEGG analysis shows the most enriched pathways in HEL^ΔSIAH2^ compared with HEL^WT^ cells, with an FDR *q* value < 0.05. The data are presented as mean ± SD of three independent experiments. Statistical analyses (unpaired *t*-test; one-way ANOVA; two-way ANOVA; Bonferroni correction; ns not significant; *P  < 0.05; **P  < 0.01; ***P  < 0.001; ****P  < 0.0001)

Since SIAH E3 ubiquitin ligases accelerate the proteasomal degradation of certain leukemogenic proteins,^17^ we hypothesized that HDAC1/HDAC2 control a previously unidentified SIAH-dependent regulation of JAK2^V617F^. Immunoblots revealed that 5 µM MERCK60 induced SIAH2, but not SIAH1, in HEL and UKE-1 cells. This increase in SIAH2 correlated inversely with phosphorylated and total JAK2^V617F^ levels (Fig. 3a and Supplementary Fig. 5a). Immunofluorescence analysis revealed that the application of 5 µM MERCK60 for 24 h significantly augmented the nuclear and cytosolic levels of SIAH2 in HEL cells (Fig. 3b). Similar observations were obtained when these cells were treated with FK228 (Supplementary Fig. 6a, b).

We further noted that silencing HDAC1 or HDAC2 but not HDAC3 increased SIAH2 levels and reduced phosphorylated and total JAK2^V617F^ in HEL cells (Fig. 3c and Supplementary Fig. 6c). This finding for HDAC3 corresponds to a lack of significant apoptosis induction upon its knockdown (Supplementary Fig. 6d). Like in HEL cells, the knockdown of HDAC1 or HDAC2 induced SIAH2 and reduced JAK2^V617F^ in SET-2 cells (Supplementary Fig. 6e, f). The treatment of K562 cells with 5 nM FK228 for 24 h and 48 h also caused an accumulation of SIAH2 and a depletion of JAK2 (Supplementary Fig. 6g).

The HDACi-induced increase in SIAH2 cannot be explained by increased *SIAH2* mRNA levels. On the contrary, FK228 and MERCK60 reduced their expression (Supplementary Fig. 6h–m). From these data we conclude that HDACi augment SIAH2 protein stability.

Acetylation stabilizes SIAH2 in gastric cancer cells.^21,22^ Testing immunoprecipitates that we formed with an anti-pan-acetylated lysine or SIAH2 antibodies, we found that FK228 induced acetylation of SIAH2 in HEL and K562 cells (Fig. 3d, e). If HDAC1 and HDAC2 control the acetylation of SIAH2, these enzymes should be identified as interaction partners of SIAH2. Immunoprecipitation experiments followed by immunoblotting verified that endogenous HDAC1 and HDAC2 can interact with endogenous SIAH2 in HEL cells (Fig. 3f).

To determine whether acetylation controls the stability and/or activity of SIAH2, we ectopically expressed JAK2^V617F^ with wild-type or acetylation mutants of SIAH2. Immunoblot analyses revealed that SIAH2, SIAH2^K139R,^ which cannot be acetylated at K139,^21,22^ and the acetylation-mimicking mutant SIAH2^K139Q^ decreased JAK2^V617F^ (Supplementary Fig. 6n). We coherently verified that MERCK60 augmented the acetylation of wild-type SIAH2 but not of SIAH2^K139R^ (Supplementary Fig. 6o).

To study whether the levels of SIAH2 determine the turnover of endogenous JAK2^V617F^, we overexpressed SIAH2, which mimics its HDACi-induced accumulation. SIAH2 overexpression depleted JAK2^V617F^ and caused an accumulation of cleaved caspase-3 and annexin-V/PI-positive HEL cells (Fig. 3g, h). To assess via a complementary loss-of-function assay whether SIAH2 determines the HDACi-induced elimination of JAK2^V617F^, we knocked down SIAH2 by RNAi. This rescued JAK2^V617F^ and significantly reduced the FK228-induced processing of caspase-3 (Fig. 3i). These results suggest that the acetylation of SIAH2 increases its levels in cells but that acetylation per se is not required for its ability to deplete JAK2^V617F^ in MPN cells.

To corroborate these data, we disrupted the *SIAH2* gene by CRISPR-Cas9 (Fig. 3j). We analyzed three clones lacking SIAH2 (HEL^ΔSIAH2^) and control clones (HEL^WT^) (Supplementary Fig. 6p,q). There was no clear HDACi-induced degradation of JAK2^V617F^ in HEL^ΔSIAH2^ cells. Consequently, HEL^ΔSIAH2^ cells did not undergo apoptosis when incubated with HDACi (Fig. 3k, l and Supplementary Fig. 6r). These processes were not due to delayed cell proliferation of HEL^ΔSIAH2^ cells. In contrast, HEL^ΔSIAH2^ cells proliferated faster than HEL^WT^ cells (Supplementary Fig. 6s).

Next, we evaluated the self-renewal capacity of JAK2^V617F^-expressing cells upon elimination of SIAH2. Compared to HEL^WT^ cells, HEL^ΔSIAH2^ cells maintained a higher renewal capacity when treated with MERCK60 (Fig. 3m). These data indicate a strong HDAC1/HDAC2-dependent link between SIAH2 and JAK2^V617F^. Coherently, pathway enrichment analysis of the upregulated genes disclosed JAK-STAT signaling as the most strongly enriched signaling pathway in the SIAH2 knockout cells (Fig. 3n).

These results demonstrate that acetylation-dependent accumulation of SIAH2 accelerates the turnover of JAK2^V617F^ and thereby promotes apoptosis of MPN cells.

### JAK2^V617F^ contains a SIAH degron in its surface-exposed kinase domain

**Fig. 4:**
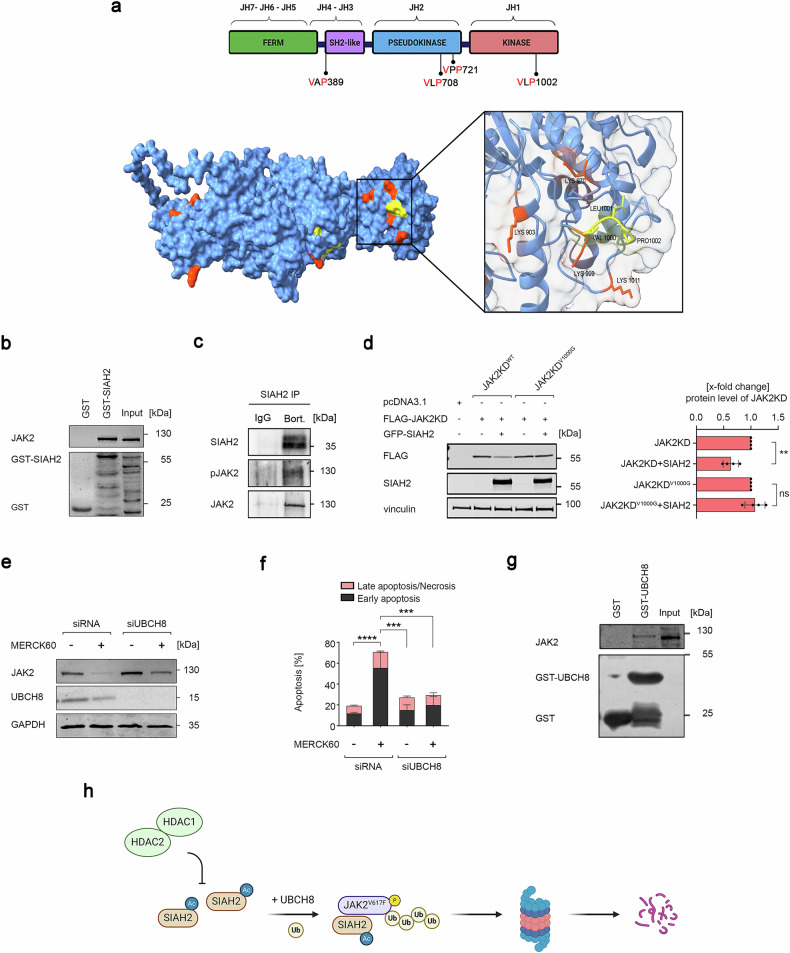
SIAH2 promotes the degradation of JAK2^V617F^ through the VxP motif in its kinase domain. **a** Upper panel, illustration depicting the organization of JAK2 into the structural JH1-7 domains and the distribution of VxP motifs within its four functional domains. Illustration was created with BioRender (https://BioRender.com). Lower panel, 3D visualization of JAK2 showing VxP motifs (yellow) and the ubiquitin-binding domain (orange) via ChimeraX V1.3. **b** SIAH2 was expressed as a glutathione S-transferase (GST) fusion protein, GST-SIAH2. Samples were purified via Glutathione Sepharose 4B beads and analyzed for JAK2 binding by immunoblotting. **c** Immunoprecipitates with anti-SIAH2 antibody or mouse preimmune serum (IgG) were analyzed for pJAK2 and JAK2 by immunoblotting. HEL cells were incubated with 50 nM bortezomib for 6 h to prevent the proteasomal degradation of SIAH2-bound JAK2^V617F^. **d** VLP degron motif in the kinase domain determines JAK2 degradation by SIAH2. FLAG-tagged kinase domains of JAK2^WT^ or mutant JAK2^V1000G^ were coexpressed with GFP-SIAH2 in HEK293T cells. Immunoblotting verified the presence of the indicated proteins, and HSP90 served as a loading control. **e** HEL cells were transfected with siRNA against UBCH8 or noncoding siRNA. The cells were subsequently treated with 5 µM MERCK60 for 48 h. Immunoblotting was used to verify the expression of JAK2^V617F^ and UBCH8; GAPDH served as a loading control. **f** HEL cells were transfected as described in (**e**), stained with annexin-V-FITC/PI and analyzed for apoptosis via flow cytometry. **g** UBCH8 was expressed as the glutathione S-transferase (GST) fusion protein GST-UBCH8. The samples were purified via Glutathione Sepharose 4B beads and analyzed for JAK2 binding by immunoblotting. **h** Illustration summarizing how HDAC1 and HDAC2 protect JAK2^V617F^ from SIAH2-mediated proteasomal degradation. SIAH2 cooperates with UBCH8 and promotes polyubiquitylation and subsequent proteasomal degradation of JAK2^V617F^. Illustration was created with BioRender (https://BioRender.com). The data are presented as mean ± SD of three independent experiments. Statistical analyses (one-way ANOVA; two-way ANOVA; ns not significant; Bonferroni correction; **P  < 0.01; ***P  < 0.001; ****P  < 0.0001)

SIAHs recognize the sequence PxAxVxP (VxP, core sequence; x, any amino acid) to bind and induce polyubiquitination and proteasomal degradation of their substrates.^17^ Such SIAH degron (“degradation on”) motifs occur in JAK2 (Fig. 4a).

To test for an interaction between JAK2^V617F^ and SIAH2, we performed an in vitro GST-pulldown assay utilizing bacterially expressed, GST-tagged SIAH2 with JAK2^V617F^ from HEL cells. We detected JAK2^V617F^ in the GST-SIAH2 pulldowns (Fig. 4b). Consistent herewith, immunoprecipitation of SIAH2 followed by immunoblotting demonstrated an interaction between SIAH2 and JAK2^V617F^ (Fig. 4c).

To functionally examine the VxP motifs in JAK2, we coexpressed SIAH2 along with the FLAG-tagged full-length JAK2 or FLAG-tagged JAK2 domains. Upon SIAH2 coexpression, the kinase domain was degraded most significantly (Supplementary Fig. 6t). To assess whether SIAH2 depletes JAK2 through the surface-exposed VxP motif in this domain (Fig. 4a), we mutated its valine residue to a sterically flexible glycine residue (V1000G). This mutation abolished the SIAH2-induced degradation of JAK2 (Fig. 4d).

Given that our transcriptome analysis revealed elevated levels of *UBE2L6* transcript which encodes the E2 ubiquitin-conjugating enzyme UBCH8 in HEL^ΔSIAH2^ cells (Supplementary Fig. 7a), we hypothesized that UBCH8 may play a critical role in the SIAH2-mediated degradation of JAK2^V617F^. We investigated if the HDACi-induced accumulation of SIAH2 ties in with an increase of its cognate E2 UBCH8. We did not observe such an increase in the mRNA levels of *UBE2L6* gene in HDACi-treated HEL or SET-2 cells (Supplementary Fig. 7b–d). Noteworthy, untreated HEL cells with hyperactive JAK2^V617F^ already carry detectable levels of UBCH8, whereas K562 cells expressing JAK2 do not (Supplementary Fig. 7e). This finding corresponds to the induction of UBCH8 by activated JAK-STAT signaling.^26^

To test if UBCH8 is required for HDACi-induced apoptosis and elimination of JAK2^V617F^, we knocked down UBCH8 by RNAi. Silencing of UBCH8 significantly abolished the MERCK60-induced degradation of JAK2^V617F^ and rescued HEL and SET-2 cells from apoptosis (Fig. 4e, f and Supplementary Fig. 7f, g). Congruent herewith, we detected JAK2^V617F^ in GST-UBCH8 pulldowns from HEL lysates (Fig. 4g). These findings support UBCH8-SIAH2-mediated degradation of JAK2^V617F^ (Fig. 4h).

The data above illustrate that a precise molecular sequence within JAK2 allows its proteasomal degradation through a UBCH8-SIAH2 signaling unit.

### MERCK60 eliminates MPN cells in vivo and displays a safe window for normal hematopoietic cells

**Fig. 5:**
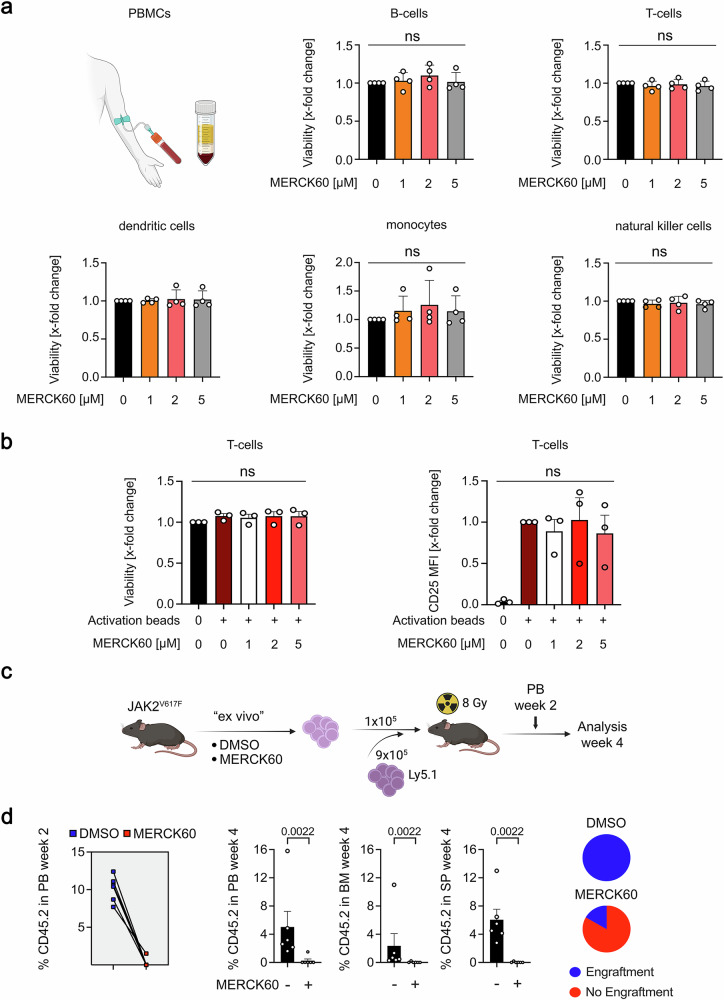
MERCK60 does not harm normal blood cells and is effective against MPN cells in vivo. **a** Fresh PBMCs were treated with increasing doses (1 µM, 2 µM, or 5 µM) of MERCK60 for 24 h. Staining for the survival markers annexin-V AF647 and FVD eFl780 was performed via flow cytometry. The isolated subtypes of cells were defined as follows: CD3-CD19+ as B-cells; CD3+ as T-cells; CD3-CD19-CD14+ as monocytes; CD3-CD19-CD1c+ as dendritic cells; CD3-CD19-CD56+ as natural killer (NK) cells. Illustration was created with BioRender (https://BioRender.com). **b** PBMCs were incubated with Dynabeads™ Human T-Activator CD3/CD28 (5 µl/ml cell suspension) for 24 h. Afterward, the cells were treated with MERCK60 (1 µM, 2 µM, or 5 µM) for 24 h. The cells were harvested and subjected to flow cytometry using FVD to discriminate live cells, CD3 as a T-cell marker, and CD25 to detect T-cell activation. **c** Scheme depicting the experimental setup. Whole bone marrow cells from JAK2^V617F^ mice were treated ex vivo with either DMSO or 5 µM MERCK60 for 24 h. JAK2^V617F^ cells (1 ×10^5^) were cotransplanted with 9 ×10^5^ cells into lethally irradiated Ly5.1 recipient mice. **d** Decrease in JAK2^V617F^-positive cells (CD45.2) in the peripheral blood (PB) at week 2 (left panel). Percentage of JAK2^V617F^-positive cells (CD45.2) at week 4 in the PB, bone marrow (BM), and spleen (SP) (middle panel). Pie charts depicting the engraftment of JAK2^V617F^-positive cells (%) after ex vivo treatment with MERCK60 or the DMSO control (right panel). The data represent mean ± SD of at least three independent experiments. Statistics (unpaired *t*-test; one-way ANOVA; ns not significant; *P* values are significant as shown)

To evaluate whether the cytotoxic effect of MERCK60 was restricted to MPN cells, we used human PBMCs from healthy donors. Since PBMCs consist of cells of the lymphoid and myeloid lineages, we differentiated individual cell types by flow cytometry using lineage-specific markers. We found that treatment with 1, 2, or 5 µM MERCK60 for 24 h or 48 h did not induce apoptosis in any of these cell populations (Fig. 5a and Supplementary Fig. 8a).

Next, we studied whether MERCK60 affected the proliferation and activity of T-cells. We stimulated T-cell proliferation with T-cell receptor activation beads. The treatment with 1, 2, or 5 µM MERCK60 for 24 h did not compromise T-cell viability or T-cell activation, as evidenced by the accumulation of CD25 **(**Fig. 5b**)**. In line herewith, up to 5 µM MERCK60 did not harm Lin^-^/Sca-1^+^/c-Kit^+^ normal murine hematopoietic stem cell progenitors (Supplementary Fig. 8b–d).

These data encouraged us to validate the in vivo efficacy of MERCK60. We aimed to investigate whether pharmacologic targeting of HDAC1 and HDAC2 is sufficient for the outcompetition of JAK2^V617F^-harboring cells by normal hematopoietic cells. To fulfill this task, we followed our previously validated assay.^5^ This assessment was facilitated by cotransplantation of murine DMSO- or MERCK60-treated JAK2^V617F^-positive whole bone marrow cells into lethally irradiated recipient mice. Analysis of peripheral blood at two weeks and peripheral blood, bone marrow, and spleen analyses at four weeks after transplantation revealed a significant reduction in the MERCK60-treated JAK2^V617F^ clone compared to DMSO-exposed control cells (Fig. 5c, d).

These findings reveal that MERCK60 induces regression of JAK2^V617F^-positive cells in vivo but exerts minimal toxicity on normal hematopoietic cells, thereby offering a potential therapeutic window.

## Discussion

We reveal a pharmacologically amenable molecular mechanism through which the nuclear enzymes HDAC1 and HDAC2 control cytoplasmic JAK2^V617F^-STAT signaling cascades. We disclose that HDAC1 and HDAC2 specifically integrate the proteasomal degradation of JAK2^V617F^ by UBCH8 and SIAH2. We additionally show that this signaling node determines MPN cell survival and offers a strategy to combat MPN cell growth in vivo. These findings provide evidence for a previously unappreciated link between epigenetic modifiers and STAT signaling as a paradigm for extra-to-intracellular signaling and the survival of leukemia cells. There are more than 600 E3 ubiquitin ligases in mammalian cells.^17,18^ We unravel that SIAH2 regulates the proteasomal degradation of JAK2^V617F^. This process is associated with the acetylation of SIAH2, which interacts with both HDAC1 and HDAC2. According to our data, the acetylation of SIAH2 promotes its stability. Congruent herewith, SIAH2^K139Q^ is more stable than SIAH2, whereas SIAH2^K139R^ is less stable than SIAH2. It is tempting to speculate that the acetylation of SIAH2 prevents its polyubiquitination for proteasomal degradation and that acetylated SIAH2 molecules do not catalyze their self-ubiquitination.^27^ We cannot exclude the possibility that SIAH2 is acetylated at further sites upon treatment with HDACi. Irrespective of such biochemical details, both of SIAH2^K139R^ and SIAH2^K139Q^ cause remarkable degradation of JAK2^V617F^. Thus, SIAH2 acetylation does not control its activity per se. Consistently, K139 does not lie in regions of SIAH2 that determine interactions with its substrates or its cognate E2 UBCH8. These results illustrate that the accumulation of stabilized SIAH2 drives the loss of mutant JAK2 in MPN cells. Although HDAC1 and HDAC2 share structural similarities and functional redundancies,^28–30^ both HDACs maintain low levels of acetylated SIAH2. Thus, each of these HDACs is a gatekeeper of SIAH2-dependent JAK2^V617F^ degradation. This epistatic function complies with the interaction of SIAH2 with both HDAC1 and HDAC2.

We present evidence that HDAC1 levels in AML cells are associated with poor patient prognosis. It is possible that aberrations in the expression of the epigenetic modifiers HDAC1/HDAC2 represent a transformation-associated control of SIAH2 and consequently of the proliferation and survival of blood cells. The expansion of myeloid progenitor cells in the bone marrow of SIAH2 mutant mice^20^ supports this hypothesis. MERCK60 is a preclinical HDACi with superior specificity for HDAC1 and HDAC2.^24^ In this work, we demonstrate that MERCK60 is significantly less toxic to normal human progenitor/stem cells than to MPN cells with highly active JAK2^V617F^-STAT signaling. Experiments with SIAH2 knockdown and SIAH2 null cells as well as unbiased transcriptomics reveal that the SIAH2-regulated JAK2^V617F^-STAT signaling causally explains such observations. Irrespective of that, HDACi deplete wild-type JAK2 in leukemia cells. As databases show that such cells do not require JAK2 for their survival, this mechanism eliminates only cells with hyperactive JAK2. Coherent with such a specific targeting of rogue cells with JAK2^V617F^, a recent report disclosed that treating aged mice with MERCK60 for two weeks did not cause any toxicity. MERCK60 even improved normal renal, cardiac, and neuronal functions.^31^ Thus, MERCK60 may offer a safe therapeutic window for treating JAK2^V617F^-driven chronic myeloid malignancies and preventing their progression to life-threatening myelofibrosis and AML. Both are major complications that can result from MPN.^2^ We tested the responses of JAK2^V617F^-positive bone marrow cells that were treated with MERCK60 and transplanted into mice. We noted that MERCK60 promoted the outcompetition of such transformed cells by normal hematopoiesis. Although ex vivo effects may have shaped this superior effect of MERCK60, our protocol for ex vivo treatment produced similar effects as the in vivo treatment validation.^5^ Thus, our findings have translational implications. Experiments evaluating more in vivo responses, including clonal tracing as well as patient-derived xenograft models, are currently needed to move toward early clinical trials. Such approaches are encouraged by the known but still underexplored responsiveness of MPN cells to HDACi,^2,12^ and a recent clinical trial that corroborated the safety of HDAC inhibition in patients suffering from leukemia. Although no dose-limiting toxicities were observed in patients receiving the selective class I/II HDACi purinostat, a group of patients with relapsed or refractory diffuse large B-cell lymphoma experienced promising response rates that suggest open-label, multicenter phase Ib/IIa trials with this HDACi.^32^ These findings testify the importance of defining patient subgroups that will benefit optimally from HDACi therapy. The results that we collected from primary human MPN patient cell samples disclose their remarkable sensitivity to specific, pharmacologically feasible inhibition of HDAC1 and HDAC2. The fact that such samples respond better than permanent MPN cell cultures to such agents further supports the consideration of selective HDACi as a therapy for patients suffering from MPN.

The structural features of JAK2 and its activation can contribute to its polyubiquitination by SIAH2 and subsequent proteasomal degradation. It has been reported that the kinase domain of JAK2 becomes surface-exposed upon activation.^33^ The constitutively active JAK2^V617F^ exposes this site, including a SIAH degron, and is hence available for interaction with SIAH2 and the ubiquitination machinery. Our finding that HDACi reduce wild-type JAK2 could indicate that its basal activity allows its degradation or that the motif that SIAH2 targets in wild-type and mutant JAK2 is accessible in transformed blood cells. Independent of such details, our data and unbiased, large-scale analyses demonstrate that leukemia cells with wild-type JAK2 are not compromised in growth when JAK2 is depleted. This finding does not imply that cells with wild-type JAK2 are resistant to HDACi. These agents can have multiple effects that can attack oncogenic drivers. For example, MERCK60 is useful to eliminate B-cell-derived tumors by not further specified mechanisms.^24^ Other isoform-specific HDACi such as the HDAC3 inhibitor RGFP966 or the HDAC10 inhibitor PZ48, target the β-catenin‒MYC or the MYC‒POLD1 axes in AML and ALL cells, respectively.^34,35^ A key task to optimally exploit the arsenal of HDACi is the identification of particularly vulnerable tumor entities and the identification of biomarkers that indicate the efficiency of HDACi in such cancers. We provide promising in vitro and in vivo data on the use of HDACi in MPN cells. We show that the pharmacological and genetic inhibition of HDAC1 and HDAC2 depletes JAK2^V617F^. Consistent with these findings, genetic experiments demonstrate that JAK2^V617F^ is the best therapeutic target in MPNs.^9^ Exploiting HDACi as therapies requires the identification of stratification markers. Our data show that the elimination of SIAH2 and UBCH8 prevents the HDACi-induced proteasomal degradation of JAK2^V617F^ and the induction of apoptosis. Consistently, SIAH2 overexpression evokes apoptosis in MPN cells. Thus, the accumulation of SIAH2 and the loss of JAK2^V617F^-STAT signaling are pharmacodynamic markers for the proapoptotic effects of HDACi.

In conclusion, our study highlights the impact of HDAC1 and HDAC2 on cellular homeostasis. These epigenetic modifiers modulate proteasomal degradation of JAK2^V617F^ and blunt proleukemogenic STAT signaling cascades through acetylation-dependent SIAH2 stabilization. Since clinically interesting, small-molecule inhibitors of HDAC1 and HDAC2 eliminate JAK2^V617F^-positive aberrant blood cells and spare normal hematopoietic stem cells, this targeted approach could benefit patients with such diseases.

## Materials and methods

### Cell cultures and growth conditions

HEL cells are JAK2^V617F^-positive human erythroleukemic cells from the peripheral blood of a 30-year-old male with PV in 1980 (AML M6 erythroleukemia, in relapse after treatment for Hodgkin lymphoma). SET-2 cells are JAK2^V617F^-positive human megakaryoblast cells that were isolated from the peripheral blood of a 71-year-old female with ET at the time of megakaryoblastic leukemic transformation in 1995. UKE-1 cells were obtained from a 59-year-old female patient with ET that transformed into AML in 1997. K562 cells were isolated from the pleural effusion of a 53-year-old female patient, and MV4-11 cells were obtained from a 10-year-old boy and express wild-type JAK2. Myeloblast-like 32D cells are murine bone marrow cells stably expressing JAK2 or JAK2^V617F^. DNA fingerprinting at the Leibniz Institute (DSMZ, Braunschweig, Germany) verified the identity of the cells. The cells were maintained under sterile standard cell culture conditions in RPMI-1640 medium supplemented with 10–20% fetal bovine serum and 1% penicillin/streptomycin (Sigma-Aldrich, Taufkirchen, Germany) in a humidified 5% CO_2_ atmosphere at 37 °C. Cell culture of HEK293T cells has been previously described.^36^ We used only mycoplasma-free cells. All cell lines were repeatedly tested for mycoplasma via PCR or enzymatic assays. Supplementary Table S1 lists detailed information about the chemicals that we used for this study.

### Primary cultures, growth conditions, and approval information

Primary JAK2^V617F^-mutated MPN patient samples and healthy donor controls were obtained after informed consent and in accordance with the Helsinki declaration from the Hematology Tumor Banks Hannover and Greifswald, which was approved by the respective local ethics committee (Ethics Committees: Hannover Medical School 11501_BO_K_2024; University Medicine Greifswald BB_199-20). EDTA-treated blood was separated with Ficoll-Paque Plus (GE Healthcare, Chicago, IL, USA) following the manufacturer’s instructions. PBMCs were plated at 1 × 10^5^ cells/replicate in MethoCult H4034 Optimum (StemCell Technologies, Vancouver, Canada) with either 0.5 or 1 µM MERCK60 or DMSO as a control. Colony numbers were counted on day 10. JAK2^V617F/+^; VavCre double-transgenic mice constitute a model system for PV.^37^ Bone marrow from the tibiae, femurs, and hip bones of PV mice were flushed out with medium using a syringe. Hematopoietic stem cells were enriched via an EasySep^TM^ Mouse Hematopoietic Progenitor Isolation Kit (StemCell Technologies #19856). The purity of the hematopoietic stem cells was verified by flow cytometry using c-Kit-BV605 clone 2B8 (BioLegend #105847) and Sca-1-BV421 (clone D7;BioLegend #108128). HSCs were cultivated in StemSpan SFEM medium (StemCell Technologies, #09600) supplemented with 20% fetal bovine serum and 1% penicillin/streptomycin. Additionally, normal untreated C57BL/6 mice were housed at the Translational Animal Research Center (TARC) of the Johannes Gutenberg University Mainz under specific pathogen-free conditions on a standard diet, according to the guidelines of the regional animal care committee (Landesuntersuchungsamt Rhineland-Palatinate). The guide for the care and use of laboratory animals as well as the 3 R principles in laboratory animal experiments were followed.^38,39^ Mice were sacrificed at the age of 12–16 weeks for organ retrieval according to § 4(3) TierSchG, the tibiae, femurs and hip bones from the hind legs were isolated, and excess muscle tissue was removed. Bone marrow tissues were flushed out with medium using a syringe, and the resulting bone marrow suspensions were filtered through 40 μm strainers. Erythrocytes were lysed, and the remaining cells were counted, cultivated in Iscove’s Modified Dulbecco’s Medium (IMDM) (Thermo Fisher Scientific, Darmstadt, Germany) supplemented with 2 mM L-glutamine, 100 U/ml penicillin, 100 µg/ml streptomycin (all from Sigma‒Aldrich, Deisenhofen, Germany), 20% fetal bovine serum (PAN-Biotech, Aidenbach, Germany) and 50 µM ß-mercaptoethanol (Carl Roth, Karlsruhe, Germany) in 12-well plates at a density of 10^6^/ml, and treated with the inhibitors. After 24 h, the cells were harvested and analyzed by flow cytometry via antibodies against Sca-1-FITC (clone: D7, eBioscience 11–5981–81), c-Kit-APC (clone: ACK2, Thermo Fisher Scientific 17–1172–82), CD3-PE (clone: 17A2, BioLegend 100206), CD4-PE (clone: RM4–5, eBioscience 12–0042–83), CD8-PE (clone: M18/2, BioLegend 101408), CD11b-PE (clone: M1/70, BD 553311), CD19-PE (clone: 1D3, PharMingen 557399), NK1.1-PE (clone: PK136, eBioscience 12–5941–83), Gr-1-PE (clone: RB6–8C5, eBioscience 12–5931–81) and Fixable Viability Dye eFluor® 780, as previously described.^40^ Immortalized mouse hematopoietic progenitors were cultivated in L-glutamine containing IMDM (Thermo Fisher Scientific, Darmstadt, Germany) supplemented with 20% fetal bovine serum, 10 ng/ml IL-3, 10 ng/ml IL-6, and 1% penicillin/streptomycin.

### In vivo animal studies and approval information

For competitive transplant assays, all the mice were housed under pathogen-free conditions in the Animal Research Facility of the Central Animal Laboratory ZTL (Hannover Medical School). All experiments were conducted after approval by the respective authorities of Lower Saxony (24--00575). We cotransplanted normal (Ly5.1) with ex vivo-treated JAK2^V617F^ cells (DMSO or 5 µM MERCK60 for 24 h) into lethally irradiated recipient mice (Ly5.1). In detail, 9 × 10^5^ normal murine whole bone marrow cells (Ly5.1; CD45.1) were transplanted along with 1 × 10^5^ JAK2^V617F^-positive (CD45.2) cells. Peripheral blood analysis was conducted at two weeks posttransplantation, and peripheral blood, bone marrow and the spleen were collected four weeks after transplantation.

### PBMCs and approval information

Buffy coats for the isolation of PBMCs from anonymous healthy blood donors were provided by the Blood Transfusion Unit of the University Medical Center Mainz, in accordance with the ethical norms established by the Nuremberg Code of 1947, the principles proclaimed in the Universal Declaration of Human Rights of 1948, and the Declaration of Helsinki of 1964, including its subsequent amendments and clarifications. The analyses were previously summarized.^41^ Antibodies for lineage-specific cell surface markers: CD3−CD19+, B-cells; CD3+, T-cells; CD3−CD19−CD14+, monocytes; CD3−CD19−CD1c+, dendritic cells; CD3−CD19−CD56+, natural killer (NK) cells. Cell viability was evaluated using annexin-V AF647 (#A23204; an early apoptosis marker) and FVD eFl780 (#65--0865--18; a late apoptosis marker) from Thermo Fisher Scientific. The following antibodies were used: anti-CD11b BV510 (#101263), anti-CD1c BV605 (#331538), and anti-CD3 BV711 (#344838) from BioLegend, San Diego, CA, USA; anti-CD14 PE-eFl610 (#61-0149-42), anti-CD56 Pe-Cy7 (#25-0567-42), anti-CD19 AF488 (#53-0199-42), and anti-CD25 APC (#17-0259-42) from Thermo Fisher Scientific. The activation of T-cells was achieved by incubating PBMCs with Dynabeads™ Human T-Activator CD3/CD28 (5 µl/1 ml cell suspension) for 24 h. Afterward, PBMCs were treated with increasing doses (1 µM, 2 µM, or 5 µM) of MERCK60 for 24 h or 48 h. T-cells were defined as CD3+ cells. FVD eFl780 staining was analyzed via flow cytometry.

### Cell lysis and immunoblotting

After incubation for 24 h, the cells were harvested by centrifugation at 317 × *g* for 5 min and washed with ice-cold phosphate-buffered saline (PBS). The cells were lysed in NET-N buffer (100 mM NaCl, 10 mM Tris-HCl pH 8, 1 mM EDTA, 10% glycerol, 0.5% NP-40; plus cOmplete protease inhibitor cocktail tablets (Roche, Mannheim, Germany) and phosphatase inhibitor cocktail 2 (Sigma‒Aldrich, Deisenhofen, Germany)), kept on ice for 30 min, sonicated (20 pulses, amplitude 40%, 0.1 s pulse duration), and centrifuged (17,000 × *g*, 25 min, 4 °C). The protein content of the lysates was estimated via Bradford assay. Proteins were detected and analyzed by SDS‒PAGE (95‒125 V for 90 min) and immunoblotting (175 mA per gel for 120 min). After being transferred onto nitrocellulose membranes (Amersham Protran, GE Healthcare), the membranes were blocked with 5% (w/v) nonfat milk for 1 h at room temperature and incubated with primary antibodies overnight at 4 °C. Afterward, the membranes were washed multiple times in Tris-buffered saline/Tween (TBS-T) for 5 min each and incubated with HRP-linked IgG (1:2000) or LI-COR infrared fluorescence secondary antibodies (1:10,000) (LI-COR, NE, USA). Proteins were detected using enhanced chemiluminescence (Western Lightning Plus-ECL, Perkin Elmer, Rodgau, Germany) and the iBright CL1000 imaging system (Invitrogen, Thermo Fisher Scientific, Darmstadt, Germany) or the Odyssey Infrared Imaging System (LI-COR, NE, USA). Densitometric analysis was performed using Image Studio Lite V4.0 software (LI-COR, NE, USA). The primary antibodies used for immunoblotting were as follows: anti-UBCH8/AP-2118a (Abgent, CA, USA); anti-UBCH8/ARP-43034 (Aviva Systems Biology, CA, USA); anti-GAPDH/ab128915, anti-HDAC3/ab32369 (Abcam, Cambridge, UK); anti-PARP1/556362, anti-STAT5/610192 (BD Bioscience, Heidelberg, Germany); anti-cleaved caspase-3/9664, anti-HDAC1/34589S, anti-pJAK2 (Y1007/Y1008)/3771, anti-JAK2/3230, anti-pSTAT1 (Y701)/9167 (Cell Signaling Technology, Frankfurt, Germany); anti-ac-H3 (K14)/06-599, anti-ac-SMC3 (K105/106)/MABE1925 (Merck Millipore, Darmstadt, Germany); anti-HSP90/ADI-SPA-830-F (Enzo Life Sciences, NY, USA); anti-SIAH1/NB300-974 (Novus Biologicals, CO, USA), anti-β-actin/sc-47778, anti-FLAG/sc-807, anti-GAPDH/sc-137179, anti-GFP/sc-9996, anti-GST/sc-138, anti-HDAC2/sc-9959, anti-HSP90/sc-13119, anti-p-JAK2 (Y1007/1008)/sc-2187, anti-STAT1/sc-346, anti-SIAH2/sc-81787, anti-pSTAT3 (Y705)/sc-7993, anti-STAT3/sc-482, anti-vinculin/sc-73614 (Santa Cruz Biotechnology, Heidelberg, Germany); anti-ac-tubulin (K40)/T7451, anti-α-tubulin/T5168 (Sigma-Aldrich, Darmstadt, Germany); anti-pSTAT5 (Y694)/MA5-14973 (Thermo Fisher Scientific, Darmstadt, Germany). The secondary antibodies used for immunoblotting included anti-rabbit IgG HRP-linked/7074), anti-mouse IgG HRP-linked/7076 (Cell Signaling Technology, Frankfurt, Germany), IRDye® 680RD anti-mouse IgG/925-68070, IRDye® 680RD anti-rabbit IgG/925-68071, IRDye® 800CW anti-mouse IgG/925-32210, and IRDye® 800CW anti-rabbit IgG/925-32211 (LI-COR, NE, USA).

### GST pulldown

SIAH2 and UBCH8 were expressed as glutathione S-transferase (GST) fusion proteins, GST-SIAH2 and GST-UBCH8, in *Escherichia coli* BL21 Gold (DE3) pLysS (Agilent Technologies, Waldbronn, Germany). Bacteria were lysed in PBS containing 0.5 mg/ml lysozyme, 1 mM dithiothreitol (DTT), 5 mM EDTA, 5 mM EGTA, 1% Triton X-100, 1% N-lauroylsarcosine, and protease inhibitors, followed by sonication (20 pulses, amplitude 40%, 0.1 s pulse duration). Glutatione Sepharose 4B beads were incubated with these lysates for 16 h at 4 °C under continuous rotation. GST only (negative control), GST-SIAH2, and GST-UBCH8 were purified via Glutathione Sepharose 4B beads according to the manufacturer’s instructions (GE Healthcare, München, Germany). Pulldowns were performed with 5 µg of bound GST-fusion proteins and 500–1000 µg of protein from HEL cell lysates in 1000 µl of NETN buffer supplemented with proteasome and phosphatase inhibitors. After incubation for 16 h at 4 °C under continuous rotation, the beads were thoroughly washed three times in PBS. The bound proteins were eluted with boiled 6x SDS sample loading buffer at 95 °C while shaking and were further assessed by immunoblotting.

### Immunoprecipitation

To further analyze the interaction between JAK2 and SIAH2, immunoprecipitations (IPs) were performed. HEL cells were treated with 5 nM FK228 for 24 h prior to harvesting. The cells were washed twice with ice-cold PBS to eliminate all residues of the RPMI-1640 medium. The cells were subsequently lysed in 1 ml of IP lysis buffer supplemented with protease and phosphatase inhibitors. After incubation for 30 min on ice, the lysates were centrifuged (18,800 × *g*, 25 min, 4 °C) before being transferred to new reaction tubes. The protein concentrations of the lysates were estimated via the Bradford assay. IP of SIAH2 was performed using 1 mg of protein per sample. The cell lysates were further incubated overnight with 1 μg of anti-SIAH2 (Santa Cruz Biotechnology, sc-81787) or anti-pan-acetylated lysine (Cell Signaling Technology, 9441) antibodies per 1 mg of protein, a mouse IgG antibody (Santa Cruz Biotechnology, sc-2025), or a rabbit IgG antibody (Santa Cruz Biotechnology, sc-2030) under moderate rotation at 4 °C. Afterward, 60 μl of protein G fast-flow Sepharose beads (50% slurry in PBS) were incubated for 4 h at 4 °C under rotation. Unbound antibodies were discarded via repeated washing steps (one time with high-salt PBS washing buffer, followed by another two washing steps with PBS washing buffer). Immunoprecipitated SIAH2 and its corresponding interaction partners were further eluted by heating the samples in 6x SDS sample loading buffer at 95 °C for 5 min while shaking. The samples were analyzed via SDS‒PAGE and immunoblotting.

### Immunofluorescence

HEL cells were treated with 5 µM MERCK60 for 24 h or 5 nM FK228 for 16 h, harvested, resuspended in 20 µl of PBS and transferred onto coverslips. The cells were subsequently fixed with acetone-methanol solution (3:7) at −20 °C for 9 min and washed once with ice-cold PBS. Blocking solution (10% BSA, 0.25% Triton X-100 in PBS) was added, and the samples were incubated for 1 h at room temperature. The cells were incubated overnight in a wet chamber at 4 °C with anti-SIAH2 (Santa Cruz Biotechnology, sc-81787) (1:500, diluted in the blocking solution). Coverslips were washed three times with PBS and incubated for 1 h at room temperature with anti-mouse AlexaFluor-488 secondary antibody from Thermo Fisher Scientific (1:300 dilution in the blocking solution). The cells were washed twice with ice-cold PBS, once with high-salt PBS (0.4 M NaCl in PBS) for 2 min and rinsed once more with PBS. Using a scalpel and tweezers, the coverslips were carefully placed on slides and mounted with 10 µl of Vectashield® (Biozol, Vec-H-1.000), containing TO-PRO-3™ (Thermo Fisher Scientific, T3605) at a dilution of 1:100 to visualize the nuclei. Images were captured via a Zeiss Axio Observer Z1 confocal microscope equipped with an LSM710 laser-scanning unit (Carl Zeiss, Jena, Germany). Analysis was performed with ImageJ software.

### Analysis of cell death by annexin-V-FITC/propidium iodine (PI) staining

The cells were collected and centrifuged (317 × *g*, 5 min) at room temperature. The cell pellets were washed twice with PBS. The resulting cell pellets were eventually resuspended in 50 μl 1x annexin-V binding buffer (1 mM HEPES, 140 mM NaCl, 0.25 mM CaCl_2_, pH was adjusted to 7.4 with KOH) and 2.5 μl annexin-V-FITC (Miltenyi Biotec, Bergisch Gladbach, Germany). Samples were incubated for 15 min on ice in darkness. Afterward, 430 μl 1x annexin binding buffer and 10 μl PI (50 μg/ml) were mixed into each sample. Proper gating of populations of interest using forward (FSC) and sideward (SSC) scatter excluded cellular debris. Cells were immediately measured with the BD FACS Canto II gadget with 488 nm excitation laser. Early apoptotic cells are annexin-V-positive and PI-negative; late apoptotic or necrotic cells are annexin-V-positive and PI-positive. Data analysis was carried out using FACSDiva software (BD Biosciences).

### Analysis of cell cycle distribution by PI staining

The cells were harvested and centrifuged (317 × *g*, 5 min). The cell pellet was washed twice in 1 ml of PBS. The cells were fixed by the addition of 2 ml of ice-cold (−20 °C) 80% ethanol dropwise while vortexing. The samples were subsequently stored at −20 °C (for at least 2 h). Prior to measurement, the samples were centrifuged (317 × *g*, 5 min), and the resulting pellets were resuspended in 334 μl of RNAse A/PBS master mix (final concentration of RNAse A for each sample: 20 μg/ml) and incubated for 1 h at room temperature. The samples were incubated for 10 min on ice following the addition of 164 μl of PI staining solution (final concentration of PI for each sample: 16.5 μg/ml). Cellular debris were outgated. The intensity of PI fluorescence signal was measured with a FACSCanto II flow cytometer (BD Biosciences) and analyzed using FACSDiva software.

### Colony formation assay

This assay shows the proliferation and self-renewal capacity of individual hematopoietic progenitor cells and whether they generate colonies in a semi-solid methylcellulose matrix. HEL and primary hematopoietic stem cells derived from MPN patients, or the murine PV model were treated with HDACi for 24 h. Post-treatment, cells were harvested, pelleted (317 × *g*, 5 min) at room temperature and washed twice with PBS. Subsequently, cells were plated evenly as triplicates (1 × 10^3^ cells/ml for HEL cells or 5 × 10^3^ cells/ml for primary cells) in methylcellulose medium (Human Methylcellulose Base Media, Catalog #HSC002, R&D Systems, MN, USA). The cells were incubated under standard cell culture conditions in a humified, 5% CO_2_ atmosphere at 37 °C. After 14 days, the colony-forming units (CFUs) were assessed and counted according to the manufacturer’s protocol via the ECHO Pro Imaging system.

### Analysis of cell proliferation

The cells were seeded at 1 × 10^4^ cells per ml in 12-well plates. Cell growth was monitored in real time by live microscopy daily over a time course of 5 days. Cell counts were determined via the TC20 automated Bio-Rad counter.

### RNA isolation and quantitative RT‒PCR

HEL, SET-2, UKE-1, K562, and MV4-11 cells were treated with FK228 for 16 h. The cells were harvested as described above. Total RNA was isolated via the NucleoSpin RNA Kit (Machery-Nagel, Düren, Germany). Afterward, cDNA was synthesized, and qPCR was performed via a C1000 thermal cycler real-time PCR device (Bio-Rad, Feldkirchen, Germany). In a final volume of 20 μl, Power SybrGreen master mix (Applied Biosystems, CA, USA), 20 ng of cDNA, and 100 nM forward or reverse primers were prepared. The experiments were carried out in technical and biological triplicate, and the ΔΔCt method was used for the analysis. β-actin and GAPDH served as housekeeping controls. The primer efficiency was maintained at 10^(−1/slope). Supplementary Table S2 lists the specific primers used.

### Plasmids

The plasmid encoding human V5-tagged JAK2^V617F^ was a kind gift from Dr. Claude Haan (University of Luxembourg). GFP-tagged sequences encoding wild-type and mutants of human SIAH2 were generously provided by Dr. Laureano de la Vega (University of Dundee). Plasmids encoding murine FLAG-tagged JAK2 and sequences encompassing its domains were generous gifts from Dr. Ludger Hengst (Medical Biochemistry Division, University of Innsbruck, Austria).^42^ A FLAG-tagged construct encoding the JAK2 kinase domain with a V1000G mutation was generated via a site-directed mutagenesis approach, as previously described.^36^ The noncoding vector pcDNA3.1 was used to ensure equal amounts of transfected DNA. The plasmids used to express the GST-SIAH2 and GST-UBCH8 fusion proteins for pulldown experiments were previously described.^43,44^

### siRNAs and transfections

Gene knockdown was performed via RNAi technology by transfecting the following SMARTpool: ON-TARGETplus siRNA: siHDAC1 (Dharmacon, L-003493-00-0005), siHDAC2 (Santa Cruz, HDAC2: sc-29346), SMARTpool: ON-TARGETplus siRNA (Dharmacon, HDAC3: L-003496-00-0005), siSIAH2 SMARTpool: ON-TARGETplus siRNA (Dharmacon, SIAH2: L-006561-00-0005), siUBCH8 Silencer^®^ Select siRNA (Thermo Fisher Scientific, *UBE2L6*: s17680) or corresponding amounts of nontargeting control siRNA (Santa Cruz, siRNA-A: sc-37007). All transfections were performed with the Neon™ Transfection System (Invitrogen, Carlsbad, CA, USA). The cells were incubated for 48 h in 6-well plates with 4 ml of fresh medium per well. Afterward, the cells were harvested for further verification and analyses. Successful knockdown was verified by immunoblotting.

### Generation of CRISPR-Cas9 SIAH2 knockout cells

Single guide RNAs (sgRNAs) were designed using the online tool Integrated Technologies (IDT) retrieved from https://www.idtdna.com. The most efficient guide RNA combination was chosen on the basis of the previously described scoring system.^45^ The sgRNAs were tested in pairs, and the most efficient combination was kept for further knockouts. Exon 1 and exon 2 of the human *SIAH2* gene were targeted using two sgRNAs. The sequences of the sgRNAs used were 5′-*GAGGTCAGCTCGTGGTGCTG*-3′ and 5′-*CAAGAGCATTACCACCCTTC*-3′. Ribonucleotide protein complexes of the sgRNAs and the GFP-tagged Cas9 enzyme were electroporated into target cells via the Neon transfection system (Thermo Fisher Scientific). Transfection efficiency was assessed 24 h later by measuring the GFP signal with flow cytometry. GFP-positive cells were sorted and cultured as single cells in 96-well plates with a BD FACSAria™ III. Single cells were maintained for approximately three weeks. Single-cell-derived colonies were screened for knockout efficiency by immunoblotting.

### 3D visualization of JAK2 structural features

The structure of JAK2 was predicted via the AlphaFold Monomer v2.0 pipeline.^46,47^ The 3D model was visualized with ChimeraX V1.3.^48^ Ubiquitination sites were mapped using data available from PhosphoSitePlus.^49^

### Site-directed mutagenesis

The FLAG-JAK2 kinase domain^42^ was mutated by overlap extension PCR using the Q5^®^ Site-Directed Mutagenesis Kit protocol (E0554) from New England Biolabs as previously described.^36^ The specific TaqMan primers used for the mouse mRNA encoding JAK2 are listed in Supplementary Table S3.

### RNA sequencing

NGS library prep was performed with Illumina’s Stranded mRNA Prep Ligation Kit following the Stranded mRNA Prep Ligation Reference Guide (June 2020) (Document # 1000000124518 v00). Libraries were prepared with a starting amount of 1000 ng and amplified in 9 PCR cycles. After PCR purification, an additional round of purification was performed using 1x AMPure XP beads. Libraries were profiled in high-sensitivity DNA via a 2100 Bioanalyzer (Agilent Technologies) and quantified using the Qubit dsDNA HS Assay Kit in a Qubit 2.0 fluorometer (Life Technologies). All nine samples (untreated HEL^WT^ cells, MERCK60-treated cells, and HEL^ΔSIAH2^ cells) were first collected as biological triplicates and subsequently pooled at equimolar ratios and sequenced on a 1 NextSeq500 high-output FC, with an SR of 1 ×79 cycles plus 10 cycles for the index read and 2 dark cycle upfront R1. Reads were aligned on the *Homo sapiens* genome assembly hg38 (ENSEMBL release 98) via STAR (version 2.7.10a, parameters: --outMultimapperOrder Random --outSAMattributes NH HI AS nM MD –outSJfilterReads Unique --outSAMunmapped Within --outFilterMismatchNoverReadLmax 0.04 –outFilterMismatchNmax 999 –sjdbOverhang 79).^50^ The featureCounts program (version 2.0.0, parameter: s2) was used to count the number of reads overlapping genes.^51^ Differentially expressed genes (DEGs) were determined via DESeq2 (release: 1.34.0) with an FDR cutoff of 1%.^52^ Gene Ontology (GO) and pathway enrichment analyses of DEGs were performed using clusterProfiler (release: 4.2.0) and ReactomePA (release: 1.38.0) packages, respectively.^53,54^

### The Cancer Genome Atlas (TCGA) and Gene Expression Profiling Interactive Analysis version 2 (GEPIA2)

We obtained data from the TCGA database, which assesses the survival of 150 AML patients in relation to HDAC1, via the Oncolnc platform (http://www.oncolnc.Org).^55^ Additionally, data from GEPIA2 rely on AML subtypes from the TCGA and GTEx databases and include gene expression quantification according to RNA sequencing.^56^ The survival of 54 AML patients was analyzed with the expression of *HDAC1* mRNA. Statistics are provided in figure legends.

### Cancer dependency map (DepMap) database analysis

Dependency data from the publicly available DepMap database via the Broad Institute’s DepMap portal (https://depmap.org/portal/) of RNAi screenings were analyzed to determine sensitivity across 25 human leukemic cells of myeloid origin toward JAK2 elimination.

### Statistical analysis

Data analyses were conducted using GraphPad Prism 8. Statistical significance was assessed via *t*-tests or one-way/two-way ANOVA. Multiple comparisons were made with Bonferroni correction. Asterisks indicate *p* values (ns not significant; ∗*p* < 0.05; ∗∗*p* < 0.01; ∗∗∗*p* < 0.001; ∗∗∗∗*p* < 0.0001). Unless otherwise stated, all experiments were performed in biological triplicates. The specifics of the statistical methods used are provided in figure legends.

## Supplementary information

Supplementary Materials

Original Immunoblots

Change_of_authorship_request_form-Journals -Complete

## Data Availability

All data that are related to this study are included in the main text or supplementary materials. The raw sequencing data generated in this study have been deposited in the Gene Expression Omnibus (GEO) database (https://www.ncbi.nlm.nih.gov/geo/) under accession number GSE243648.
