# Supplementary material for: The deacetylases HDAC1/HDAC2 control JAK2V617F-STAT signaling through the ubiquitin ligase SIAH2
Source: Signal Transduct Target Ther. 2025 Aug 29;10:275. doi: 10.1038/s41392-025-02369-7 (PMC12394589; doi:10.1038/s41392-025-02369-7)
Supplement: Supplementary file 1 — Supplementary Materials [file 41392_2025_2369_MOESM1_ESM.docx]

Supplementary Materials for

The deacetylases HDAC1/HDAC2 control JAK2^V617F^-STAT signaling through the ubiquitin ligase SIAH2

Al-Hassan M. Mustafa^1,2,*^, Giuseppe Petrosino^3^, Marten A. Fischer^1^, Tina M. Schnöder^4^, Désirée Gül^5^, Yanira Zeyn^6^, Christoph Hieber^6^, Johanna Lossa^7^, Sabine Muth^7^, Markus P. Radsak^8^, Walburgis Brenner^9^, Markus Christmann^1^, Matthias Bros^6^, Florian H. Heidel^4,10^, and Oliver H. Krämer^1,*^

Correspondence to: alabdeen@uni-mainz.dealabdeen@uni-mainz.de, https://orcid.org/0000-0002-4453-2573 or [okraemer@uni-mainz.de](mailto:okraemer@uni-mainz.de), https://orcid.org/0000-0003-3973-045X

**This file includes:**

Figures. S1 to S8

Tables S1 to S3

**
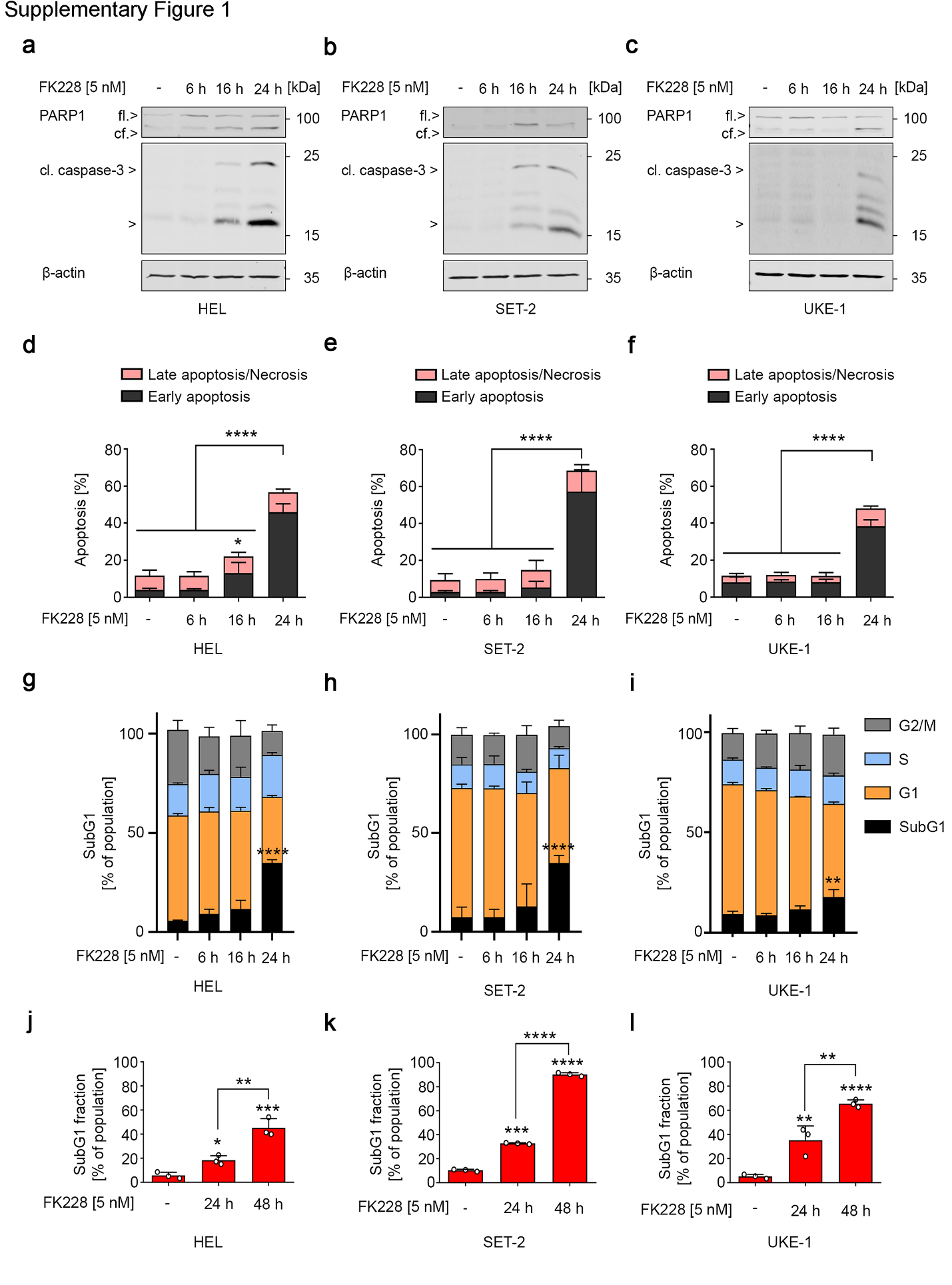
**

Figure. S1. FK228 induces apoptosis in JAK2^V617F^-positive cells. a-c HEL, SET-2, and UKE-1 cells were treated with 5 nM FK228 for a time course over 6, 16 to 24 h. Immunoblotting shows cleavage of PARP1 and caspase-3; β-actin served as a loading control. d-f HEL cells were incubated with 5 nM of FK228 as described in (a-c), stained with annexin-V-FITC/PI, analyzed for apoptosis by flow cytometry. g-i HEL cells were incubated with 5 nM FK228 as described in (a-i), fixed, and stained with PI. Cell cycle distribution and subG1 fraction were analyzed via flow cytometry. j-l HEL cells were incubated with 5 nM FK228 for 24 and 48 h, fixed, stained with PI, and analyzed for subG1 fraction accumulation by flow cytometry. The data show mean ± SD of three independent experiments. Statistics (one-way ANOVA; two-way ANOVA; Bonferroni correction; *P < 0.05; **P < 0.01; ***P < 0.001; ****P < 0.0001).


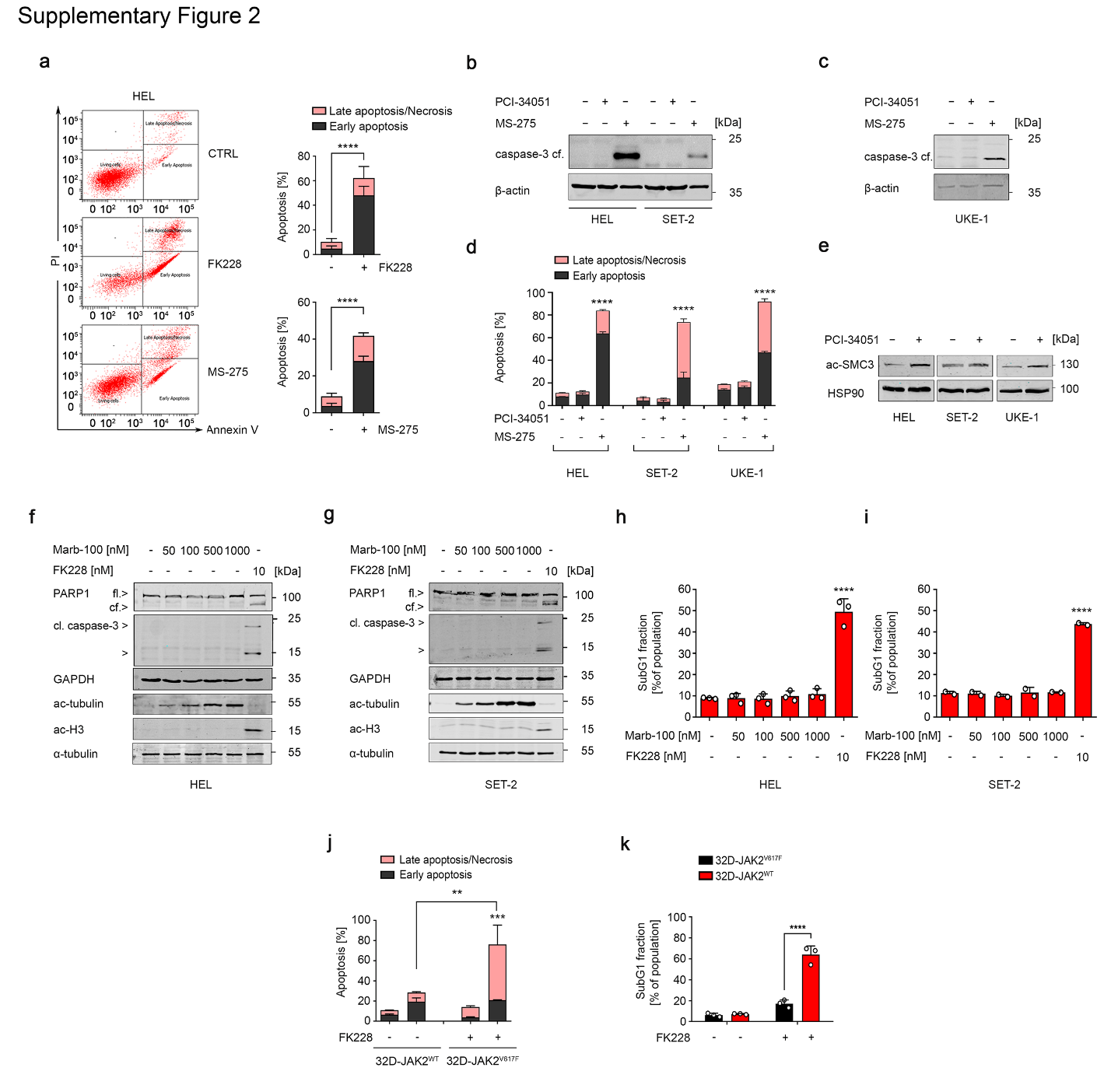


Figure. S2. Class I HDACi induce apoptosis in JAK2^V617F^-positive cells. a HEL were treated with 5 nM FK228 or 5 μM MS-275 for 24 h. The cells were stained with annexin-V-FITC/PI and analyzed for apoptosis via flow cytometry. b,c HEL, SET-2, and UKE-1 cells were treated with 5 µM PCI-34051 or 5 µM MS-275 for 48 h. The cells were lysed and cleavage of caspase-3 was analyzed via immunoblotting; β-actin served as a loading control. d HEL, SET-2, and UKE-1 cells were treated as described in (b,c), stained with annexin-V-FITC/PI, and analyzed for apoptosis by flow cytometry. e HEL, SET-2, and UKE-1 cells were treated with 5 µM PCI-34051 for 48 h. The cells were lysed and analyzed for hyperacetylation of SMC3 (ac-SMC3) by immunoblotting; HSP90 served as a loading control. f,g HEL and SET-2 cells were treated with increasing concentrations (50-1000 nM) of marbostat-100 (Marb-100) or 5 nM FK228 for 24 h. The cells were lysed and indicated proteins were analyzed by immunoblotting; GAPDH and α-tubulin served as loading controls. h,i HEL and SET-2 cells were treated as described in (f,g), fixed, stained with PI, and analyzed for subG1 fractions by flow cytometry. j,k (32D-JAK2^WT^ and 32D-JAK2^V617F^) cells were incubated with 5 nM FK228 for 24 h. The cells were stained with annexin-V-FITC/PI or PI and analyzed for apoptosis or subG1 fraction via flow cytometry. The data represent mean ± SD of at least two independent experiments. Statistics (one-way ANOVA; two-way ANOVA; Bonferroni correction; ns: not significant; **P < 0.01; ***P < 0.001; ****P < 0.0001).


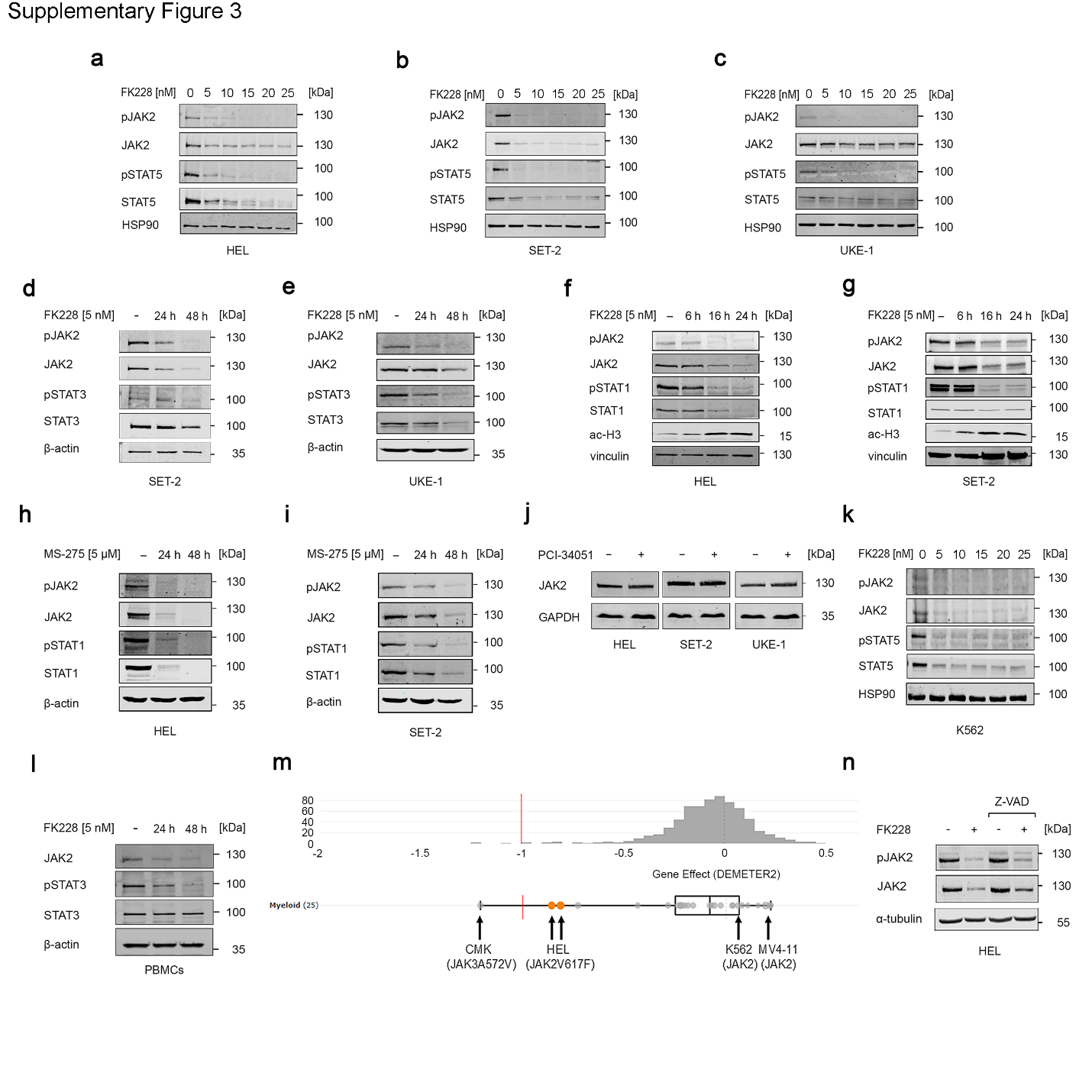
Figure. S3. Class I HDACi abrogate JAK-STAT signaling in JAK2^V617F^-positive MPN cells. a-c HEL, SET-2, and UKE-1 cells were treated with increasing concentrations of FK228 (5-25 nM) for 24 h. The cells were lysed and indicated proteins were analyzed by immunoblotting; HSP90 served as a loading control. d,e SET-2 and UKE-1 cells were treated with 5 nM FK228 for 24 and 48 h. Indicated proteins were analyzed by immunoblotting; β-actin served as a loading control. f,g HEL and SET-2 cells were treated with 5 nM FK228 for a time course over 6, 16 to 24 h. The cells were lysed and indicated proteins were detected by immunoblotting; vinculin served as a loading control. h,i HEL and SET-2 cells were incubated with 5 µM MS-275 for 24 and 48 h. Phosphorylated and total JAK2 and STAT1 were detected by immunoblotting; β-actin served as a loading control. j HEL, SET-2, and UKE-1 cells were treated with 5 µM PCI-34051 for 48 h. The cells were lysed and protein levels of JAK2^V617F^ were analyzed via immunoblot; GAPDH served as a loading control. k K562 cells were treated with increasing concentrations of FK228 (5-25 nM) for 24 h. The cells were lysed and indicated proteins were analyzed by immunoblotting; HSP90 served as a loading control. l PBMCs from healthy donors were treated with 5 nM FK228 for 24 and 48 h. The cells were lysed and indicated proteins were analyzed by immunoblotting; β-actin served as a loading control. m Analyses in the DepMap database show the dependency of MPN cells on JAK2^V617F^ compared with leukemic cells expressing wild-type JAK2. n HEL cells were incubated with 5 nM FK228 and/or 50 μM z-VAD-FMK for 24 h. Phosphorylated and total JAK2 were detected by immunoblotting; α-tubulin served as a loading control.


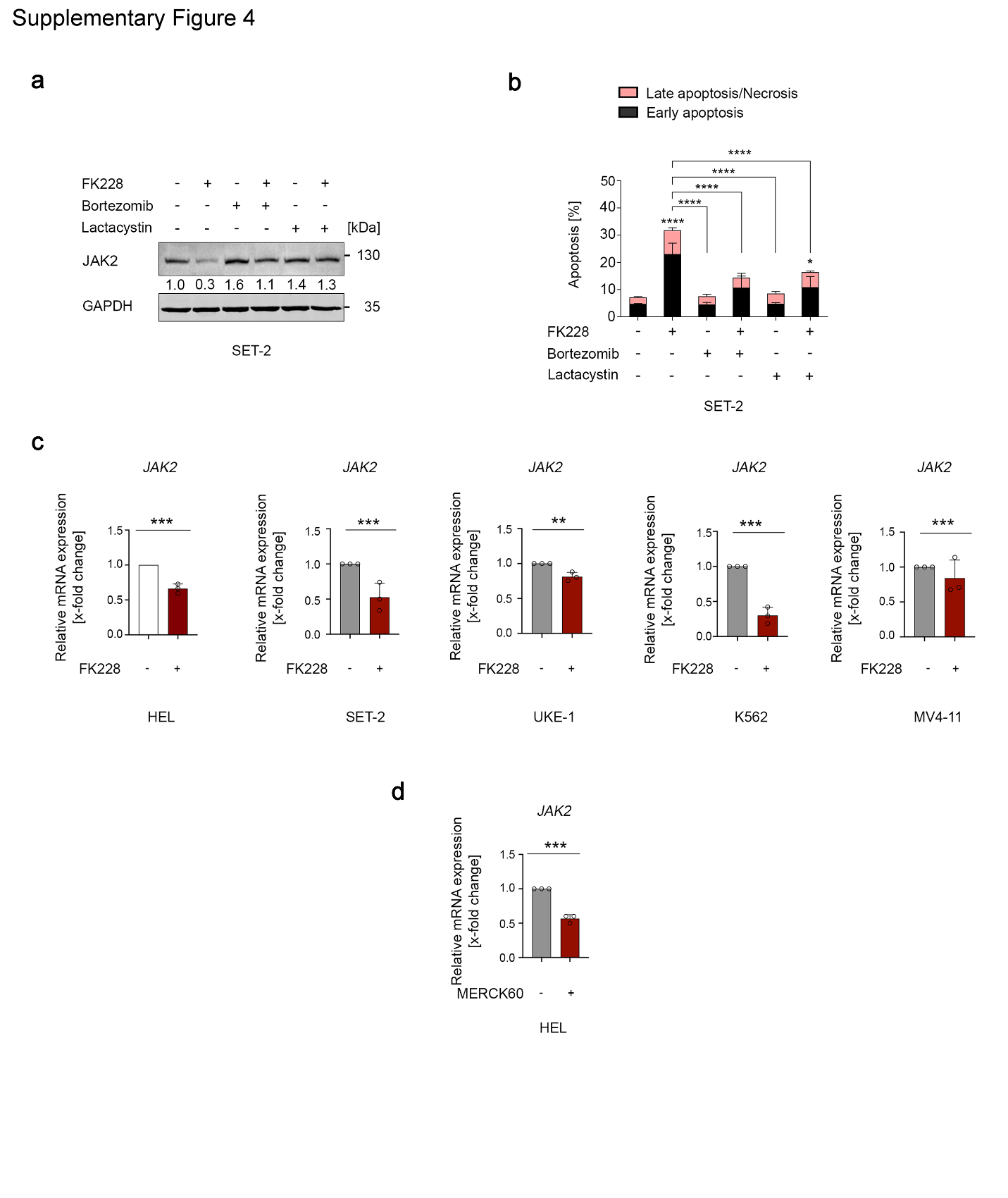
Figure. S4. FK228 induces proteasomal degradation of JAK2^V617F^ and depletes mRNA levels of JAK2^V617F^ and JAK2 in leukemic cells. a SET-2 cells were treated with 5 nM FK228 for 24 h and/or proteasome inhibitors (50 nM bortezomib; 10 μM lactacystin) for 6 h before harvesting them. Immunoblotting shows JAK2 levels and GAPDH served as a loading control b SET-2 cells were treated as described in (a), stained with annexin-V-FITC/PI, and analyzed for apoptosis by flow cytometry. c The expression of *JAK2* was measured by qPCR shown as fold change in the indicated cell lines treated with 5 nM FK228 for 16 h compared with untreated cells. d RNA sequencing of HEL cells shows mRNA levels of *JAK2* in response to MERCK60. HEL cells were treated with 5 µM MERCK60 for 24 h and total RNA was extracted. Purified total RNA was subjected to NextSeq500 and mRNA levels of *JAK2* were analyzed. The data represent at least three independent experiments as mean ± SD. Statistics (unpaired t-test; one-way ANOVA; two-way ANOVA; Bonferroni correction; **P < 0.01; ***P < 0.001; ****P < 0.0001).


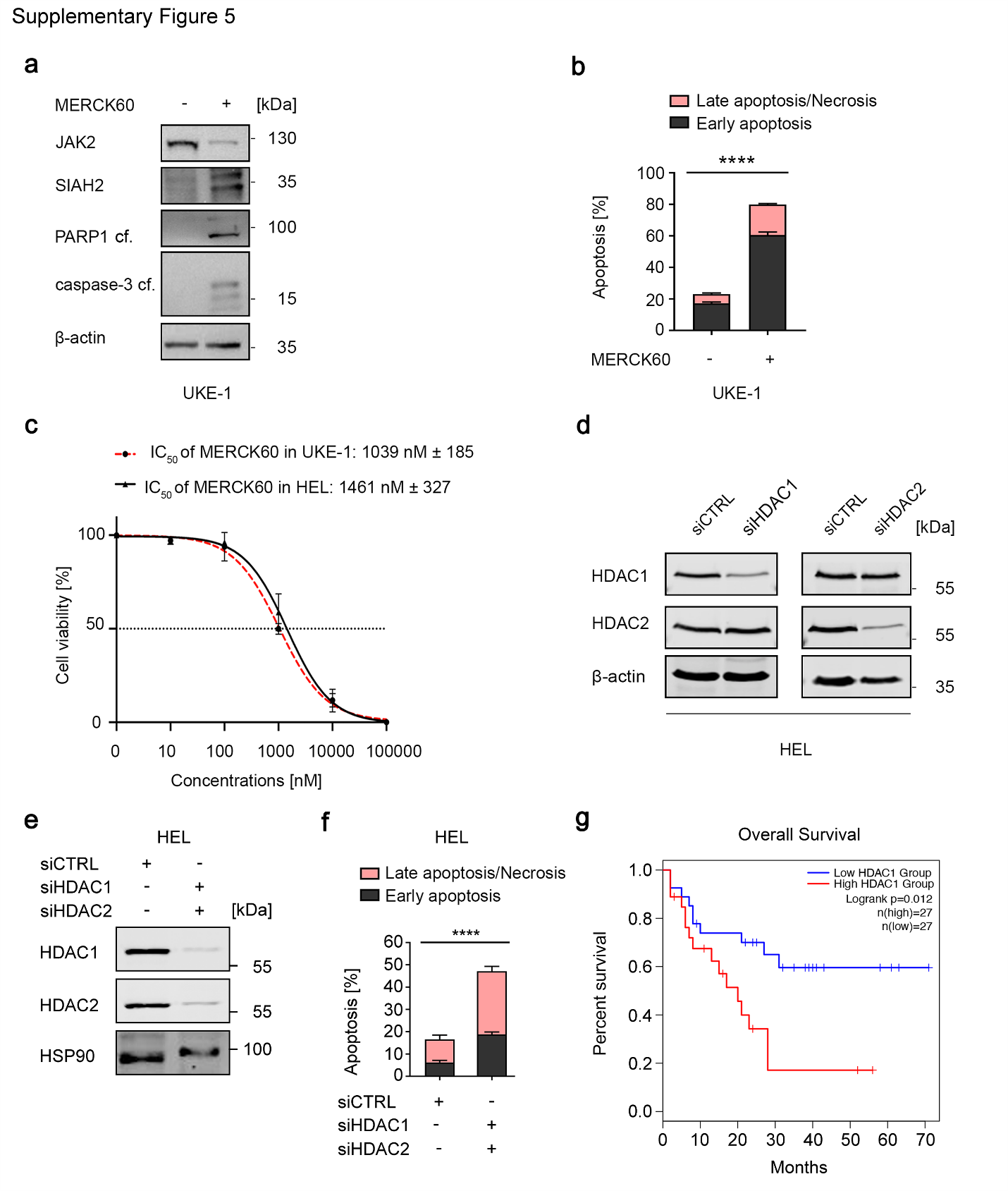
Figure. S5. MERCK60 and efficient knockdown of HDAC1/HDAC2 induce apoptosis in MPN cells and survival data of AML patients from GEPIA2. a UKE-1 cells were incubated with 5 μM MERCK60 for 48 h. The cells were lysed and the indicated proteins were analyzed by immunoblotting; β-actin served as a loading control. b HEL cells were treated as described in (a), stained with annexin-V-FITC/PI, and analyzed for apoptosis by flow cytometry. c HEL and UKE-1 cells were treated with increasing concentrations of MERCK60 for 48 h. IC_50_ values were determined from three biological replicates. d HEL cells were transfected individually with siRNAs against HDAC1 or HDAC2 for 48 h. The efficient knockdown of HDACs was validated by immunoblotting. β-actin served as loading controls. e A dual knockdown of HDAC1 or HDAC2 using siRNA for 48 h in HEL cells. The efficient knockdown of HDACs was validated by immunoblotting. HSP90 served as a loading control. f HEL cells were transfected as mentioned in (e), stained with annexin-V-FITC/PI, and analyzed for apoptosis by flow cytometry. g GEPIA2 database shows that the overall survival of AML patients (n=54) inversely correlates with the expression of *HDAC1* mRNA. The data represent at least three independent experiments as mean ± SD. Statistics (one-way ANOVA; two-way ANOVA; Bonferroni correction; ns: not significant; ****P < 0.0001).


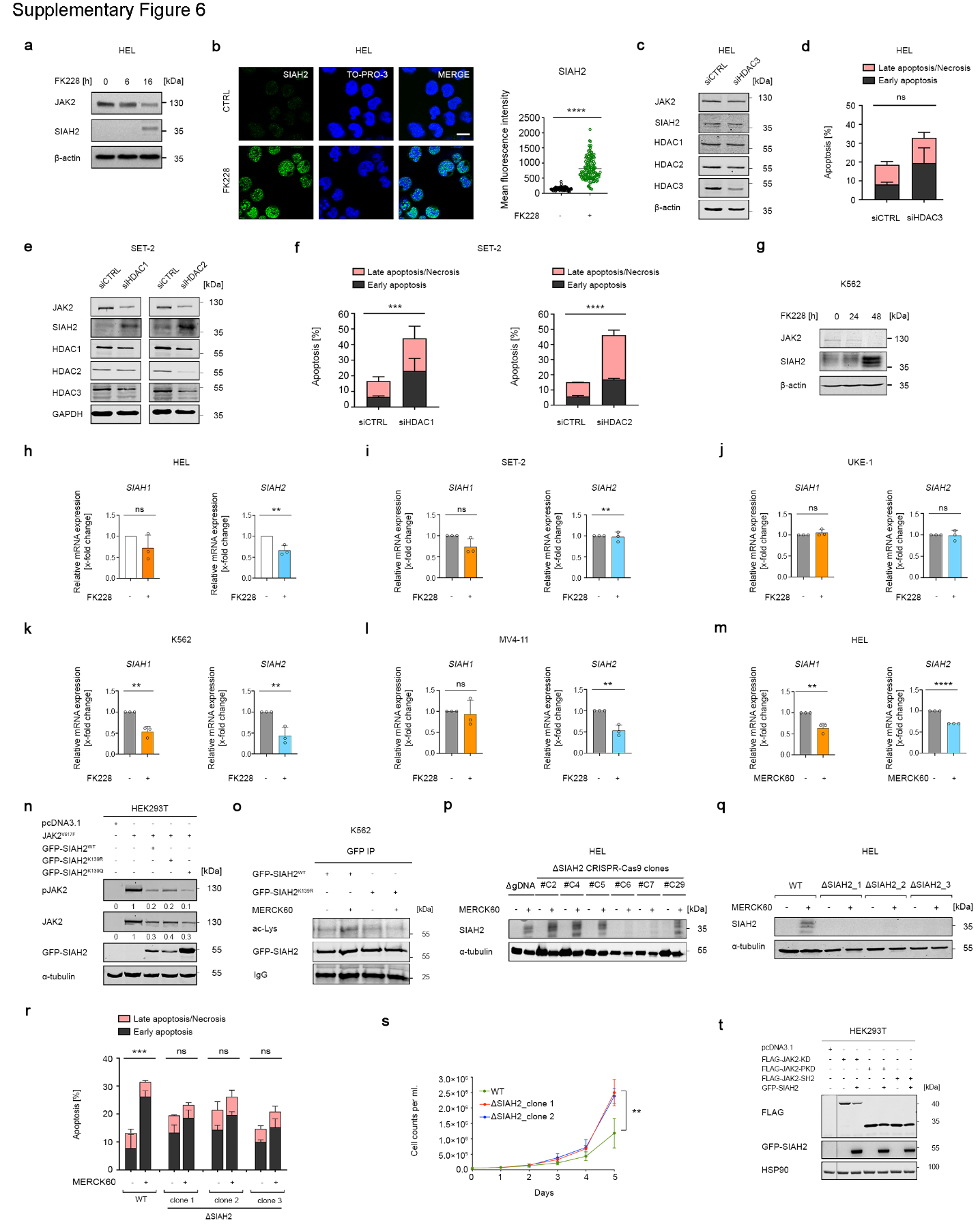


Figure. S6. Acetylation-dependent stabilization of SIAH2 targets JAK2^V617F^ for proteasomal degradation. a HEL cells were treated with 5 nM FK228 for a time course up to 16 h. Protein levels of JAK2^V617F^ and SIAH2 were assessed by immunoblotting; β-actin served as a loading control. b HEL cells were treated with 5 nM FK228 for 16 h. The cells were fixed, and immunofluorescence was performed anti-SIAH2 antibody (green). TO-PRO-3 was used to visualize the cell nuclei (blue). The cells were examined under confocal laser scan microscopy. Representative images are shown; n=3; scale bar, 10 µm (left panel). Mean fluorescence intensities were measured with ImageJ software (right panel). c HEL cells were transfected with siRNAs against HDAC3 for 48 h. Efficient knockdown of HDAC3 and protein levels of the indicated proteins were analyzed by immunoblotting. d Aliquots from HEL cells that were transfected in (c), stained with annexin-V-FITC/PI, and analyzed for apoptosis by flow cytometry. e SET-2 cells were transfected with the indicated siRNAs against HDAC1 and HDAC2 for 48 h. Efficient knockdown of HDACs and protein levels of the indicated proteins were analyzed by immunoblotting. β-actin served as a loading control. f HEL cells were transfected as stated in (d), stained with annexin-V-FITC/PI, and analyzed for apoptosis by flow cytometry. g K562 cells were incubated with 5 nM FK228 for 24 and 48 h. Protein levels of JAK2 and SIAH2 were detected by immunoblotting; β-actin served as a loading control. h-l The expression of *SIAH1* and *SIAH2* were measured by qPCR shown as fold change in the indicated cell lines treated with 5 nM FK228 for 16 h compared with untreated cells. m RNA sequencing of HEL cells shows mRNA levels of the indicated transcripts in response to MERCK60. HEL cells were treated with 5 µM MERCK60 for 24 h and total RNA was extracted. Purified total RNA was subjected to NextSeq500 and mRNA levels of the indicated genes were analyzed. n HEK293T cells were transfected with pcDNA3.1, V5-JAK2^V617F^ or GFP-SIAH2 plasmids as indicated for 48 h. Immunoblot verifies the expression of the V5-JAK2^V617F^ and/or GFP-SIAH2 plasmids (SIAH2^WT^, SIAH2^K139R^, or SIAH2^K139Q^) and the specificity for their individual targets. o Analysis of K139 acetylation in SIAH2^WT^ and mutant SIAH2^K139R^. K562 cells were transfected with two plasmids expressing GFP-tagged SIAH2^WT^ and GFP-tagged SIAH2^K139R^ and treated with 5 µM MERCK60 for 48 h. Immunoprecipitates with anti-GFP antibody or mouse preimmune serum (IgG) were analyzed for pan-acetylated lysine (ac-K), GFP-SIAH2, and IgG via immunoblot. p,q HEL^WT^ and different clones of HEL^ΔSIAH2^ cells were treated with 5 µM MERCK60 for 48 h. Immunoblotting verifies the expression of JAK2^V617F^ and/or SIAH2 in HEL^ΔSIAH2^ compared with HEL^WT^ cells. α-tubulin served as a loading control. r HEL^WT^ and HEL^ΔSIAH2^ cells were treated as in (q), stained with annexin-V-FITC/PI and analyzed for apoptosis via flow cytometry. s Proliferation rates of HEL^WT^ and two different HEL^ΔSIAH2^ cells were assessed for 5 days. The graph shows the cell counts of in HEL^ΔSIAH2^ compared with HEL^WT^ cells. t Cotransfection of full-length JAK2 or JAK2 domains (SH2, pseudokinase, kinase) with GFP-SIAH2 in HEK293T. Transfection of 1 μg FLAG-tagged, mouse JAK2 full-length (131 kDa), JAK2 kinase domain (46 kDa), JAK2 pseudokinase domain (27 kDa), JAK2 SH2-like domain (27 kDa), and JAK2 FERM domain (35 kDa) were transfected with or without 0.2 μg GFP-SIAH2 (62 kDa) plasmid into HEK293T cells and expressed for 48 h. pcDNA3.1 was used as an empty control. Immunoblotting verifies the expression of the indicated proteins. HSP90 served as a loading control. Data represents three independent experiments as mean ± SD. Statistics (unpaired t-test; one-way ANOVA; two-way ANOVA Bonferroni correction; ns: not significant; **P < 0.01; ***P < 0.001; ****P < 0.0001).


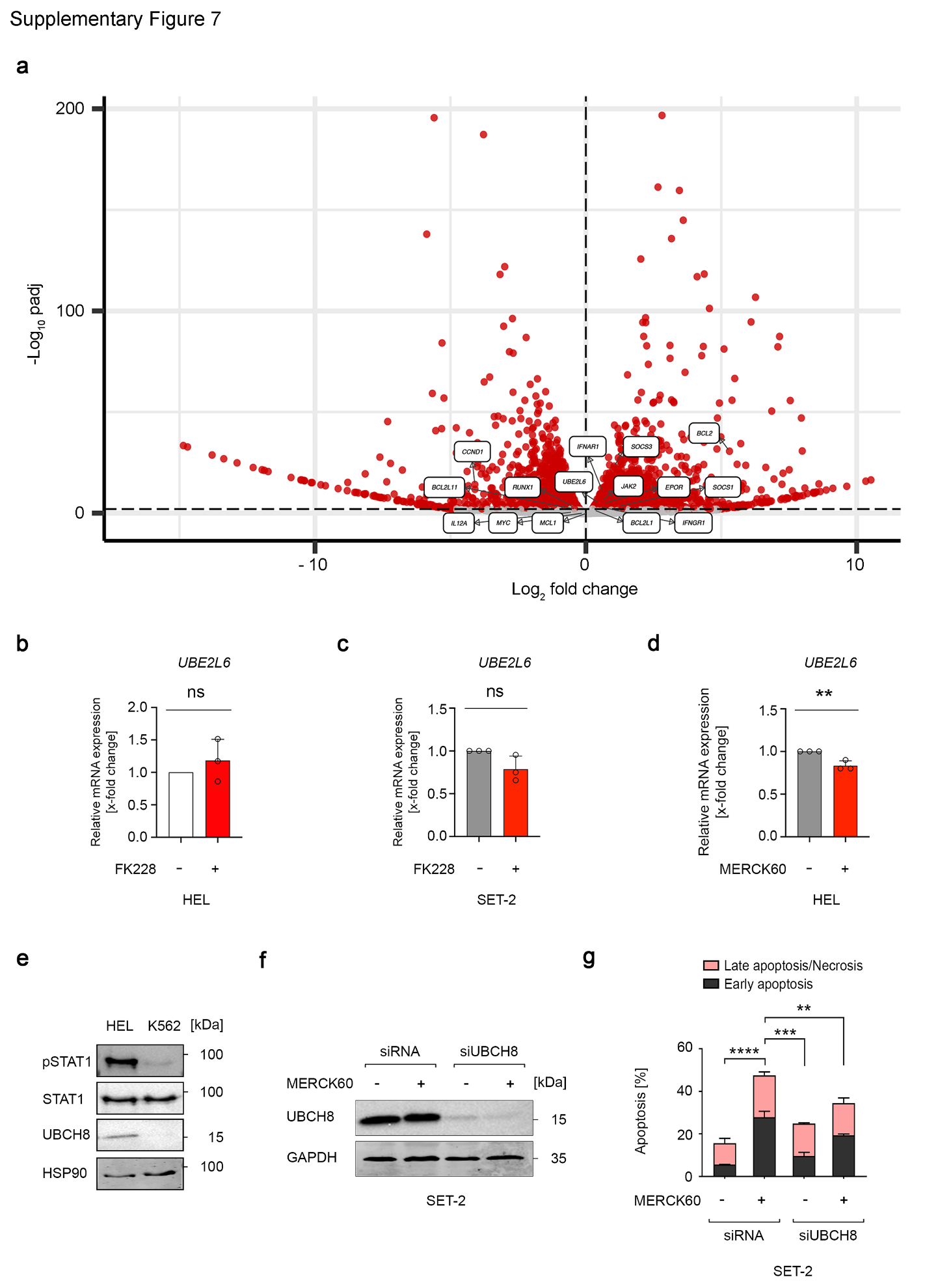
Figure. S7. UBCH8 contributes the MERCK60-induced apoptosis in the JAK2^V617F^-positive cells. a Volcano blot shows differently expressed proteins of HEL and HEL^ΔSIAH2^ cells. b,c The expression of *UBE2L6* was analyzed by qPCR shown as fold change in HEL and SET-2 cells treated with 5 nM FK228 for 16 h compared with untreated cells. d RNA sequencing analysis shows mRNA levels of *UBE2L6* as fold change in MERCK60-treated HEL cells compared with untreated cells. HEL cells were treated with 5 µM MERCK60 for 24 h and total RNA was extracted. Purified total RNA was subjected to NextSeq500 and mRNA levels of *UBE2L6* were analyzed. e A comparison of basal levels of phosphorylated and total STAT1 and UBCH8 in HEL and K562 cells by immunoblotting. HSP90 served as a loading control. f SET-2 cells were transfected with siRNA against UBCH8 or noncoding siRNAs. The cells were thereafter treated with 5 µM MERCK60 for 48 h. Immunoblotting verifies the expression of JAK2^V617F^ and UBCH8; GAPDH served as a loading control. g HEL cells were transfected as mentioned in (f), stained with annexin-V-FITC/PI and analyzed for apoptosis via flow cytometry. Data represents at least three independent experiments as mean ± SD. Statistics (unpaired t-test; one-way ANOVA; two-way ANOVA; Bonferroni correction; ns: not significant; **P < 0.01; ***P < 0.001; ****P < 0.0001).


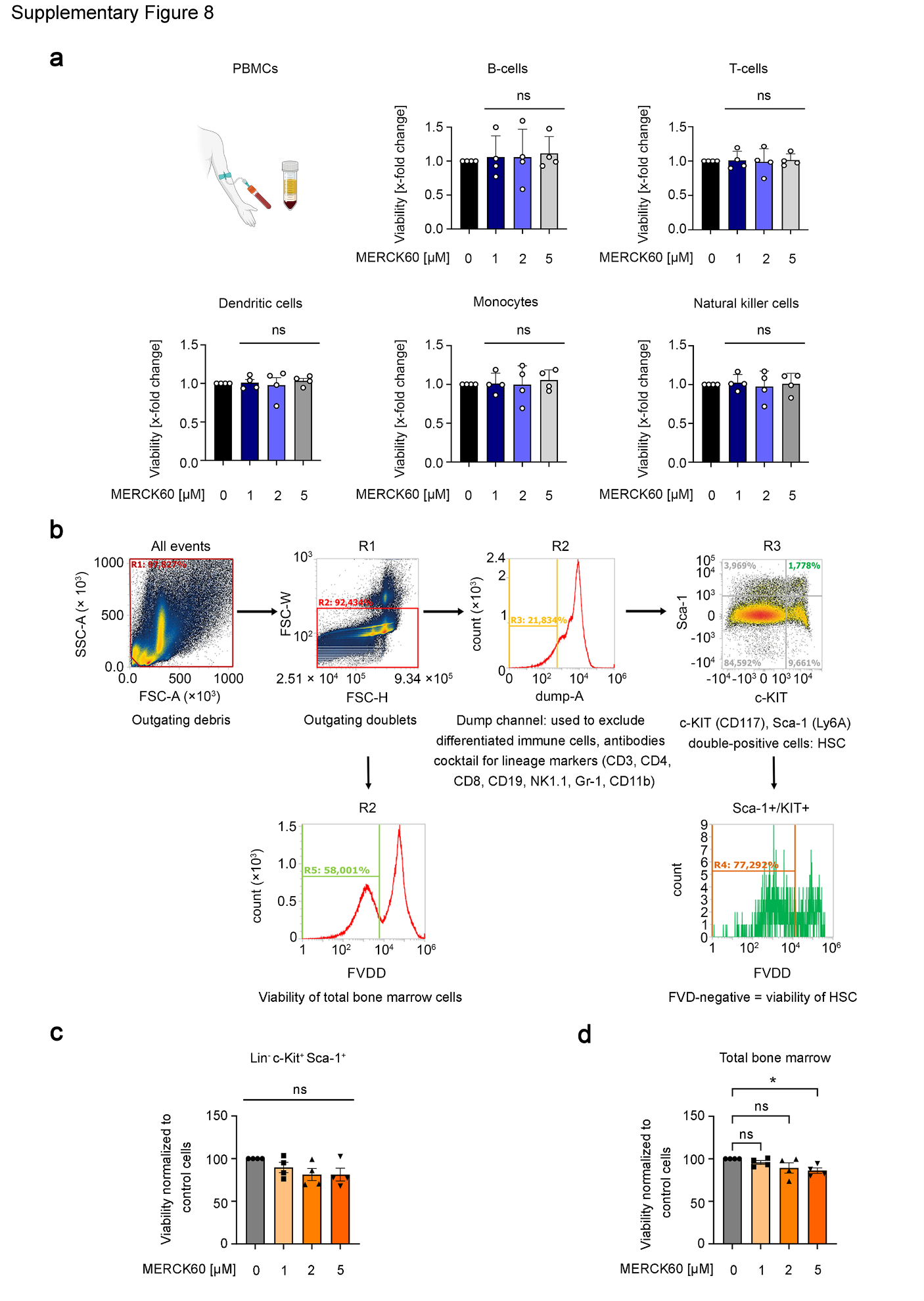
Figure. S8. MERCK60 does not harm normal blood cells and murine hematopoietic progenitor cells. a PBMCs were treated with increasing doses (1 µM, 2 µM or 5 µM) of MERCK60 for 24 h. Staining of annexin-V AF647 and FVD eFl780 was analyzed using flow cytometry. Isolated subtypes of cells were defined as: CD3-CD19+ as B-cells; CD3+ as T-cells, CD3-CD19-CD14+ as monocytes; CD3-CD19-CD1c+ as dendritic cells; CD3-CD19-CD56+ as natural killer (NK) cells; and CD3-CD14-CD19-CD56-CD11b+ as PMNs. Illustration was created in https://BioRender.com. b-d Freshly isolated murine hematopoietic progenitors were isolated from the bone marrow tissue of C57BL/6 mice. The cells were incubated ex vivo with increasing doses of MERCK60 as described in (a) for 24 h. The cells were harvested and analyzed by flow cytometry. The viability of total bone marrow cells as well as cells that were negative for lineage markers (Lin-; CD3, CD19, CD11b, NK1.1, Gr-1, CD4, CD8) but double-positive for Sca-1 and c-Kit was analyzed with the Viability Dye eFluor® 780. Results represent four independent experiments (n=4, mean ± SD; one-way ANOVA; ns: not significant; *P < 0.05).

Table S1.

| **Item** | **Provider** | **Identifier** |
| --- | --- | --- |
| Bortezomib | Sigma-Aldrich | Cat#: 504314 |
| Entinostat (MS-275) | Selleck Chemicals | Cat#: S1053 |
| N-Acetyl-L-cysteine (NAC) | Sigma-Aldrich | Cat#: A9165 |
| Romidepsin (FK228) | Selleck Chemicals | Cat#: S3020 |
| 40% Acrylamide/bisacrylamide 37.5:1 | Carl Roth | Cat#: T802.1 |
| Acetone | Carl Roth | Cat#: CP40.1 |
| Agarose standard | Carl Roth | Cat#: 3810.3 |
| Ammonium persulfate (APS) | Carl Roth | Cat#: 9592.2 |
| Annexin-V FITC | Miltenyi Biotec | Cat#: 130-093-060 |
| Bovine serum albumin (BSA) Fraction V | Carl Roth | Cat#: 1ET9.3 |
| CaCl_2_ | Merck | Cat#: 6781 |
| Coomassie Brilliant Blue G-250 | Merck | Cat#: 115444 |
| Dimethyl sulfoxide (DMSO) | Sigma-Aldrich | Cat#: D8418 |
| Dithiothreitol (DTT) | Carl Roth | Cat#: 6908 |
| Dulbecco’s Modified Eagle’s Medium | Sigma-Aldrich | Cat#: D5796 |
| EGTA | Sigma-Aldrich | Cat#: 3054.1 |
| Entinostat (MS-275) | Selleck Chemicals | Cat#: S1053 |
| Ethanol | Carl Roth | Cat#: 9065.4 |
| Ethylenediaminetetraacetic acid (EDTA) | Applichem | Cat#: 131669.1211 |
| Fetal bovine serum | PAN-Biotech | N/A |
| Fetal bovine serum | Sigma-Aldrich | Cat#: S0615 |
| Fetal bovine serum | Thermo Fisher Scientific | Cat#: 16000044 |
| Gibco OptiMEM | Thermo Fisher Scientific | Cat#: 31985070 |
| Gibco Penicillin/streptomycin | Thermo Fisher Scientific | Cat#: 15140122 |
| Glycerin | Sigma-Aldrich | Cat#: G5516 |
| Glycine | Carl Roth | Cat#: 0079.4 |
| Hydrochloric acid (HCl) | Carl Roth | Cat#: 4625 |
| Hydroxyethyl piperazineethane sulfonic acid (HEPES) | Sigma-Aldrich | Cat#: H3375-100G |
| IPTG | Carl Roth | Cat#:2316.3 |
| Isopropanol (2-Propanol) | Fisher Scientific | Cat#: P/7500/15 |
| Lactacystin | Sigma-Aldrich | Cat#: L6785 |
| Lipofectamine® 3000 | Invitrogen | Cat#: L3000-008 |
| Marbostat-100 | Sellmer et al. | Journal of medicinal chemistry 61, 3454-3477 (2018) |
| MERCK60 (BRD6929) | Sigma-Aldrich | Cat#: SML2521 |
| Methanol | Carl Roth | Cat#: 4627.2 |
| NaCl | Sigma-Aldrich | Cat#: 3957 |
| New Blot™ Nitro Stripping Buffer, 5x | LI-COR | Cat#: 928-40030 |
| Non-fat dry milk powder | Carl Roth | Cat#: T145 |
| NP-40 | Fluka Analytical | Cat#: 74385 |
| N-lauroylsarcosine sodium | Sigma-Aldrich | Cat#: L5125 |
| PageRulerTM prestained protein ladder | Thermo Fisher Scientific | Cat#: 26616 |
| PageRuler^TM^ prestained protein ladder | Thermo Fisher Scientific | Cat#: 26619 |
| PCI-34051 | Med Chem Express | Cat#: HY-15224 |
| Phosphatase inhibitor cocktail 2 | Sigma-Aldrich | Cat#: P5726 |
| Phosphate-buffered saline (PBS) powder | Bio & Sell | Cat#: BS.L182-50 |
| Phosphoric acid (85% (w/v) in H_2_O) | Carl Roth | Cat#: 90791 |
| Potassium Chloride | Carl Roth | Cat#: 6781 |
| Propidium iodide (PI) | Sigma-Aldrich | Cat#: P4864 |
| protease inhibitor (cOmplete tablets, Mini, EDTA-free, EASYpack) | Roche | Cat#: 11697498001 |
| Puromycin | InvivoGen | Cat#: ant-pr-1 |
| RNAse A | Sigma-Aldrich | 10109142001 |
| RNASE AWAY | Molecular Bioproduct | Cat#: 7003 |
| RPMI-1640 | Sigma-Aldrich | Cat#: R8758 |
| Serva Blue G | Serva Electrophoresis | Cat#: 35050 |
| Sodium dodecyl sulfate (SDS) | Carl Roth | Cat#: 8029 |
| Sodium hydroxide | Carl Roth | Cat#: 6771 |
| Sodium pyruvate | Fisher Scientific | Cat#: 12539059 |
| Tetramethylethylenediamine (TEMED) | Carl Roth | Cat#: 2367 |
| Tris base | Carl Roth | Cat#: 5429 |
| Triton X-100 | Sigma-Aldrich | Cat#: X100 |
| Trypsin-EDTA | Thermo Fisher Scientific | Cat#: 15090046 |
| Tween-20 | Carl Roth | Cat#: 9127 |
| Western Lightening Plus-ECL | PerkinElmer | Cat#: NEL104001EA |
| Z-VAD-FMK | Selleck Chemicals | Cat#: S7023 |
| Critical commercial assays | | |
| EasySep^TM^ Mouse Hematopoietic Progenitor Isolation Kit | StemCell | Cat#: 19856 |
| MethoCult Optimum | StemCell | Cat#: H4034 |
| NucleoSpin RNA, Mini kit for RNA purification | Machery-Nagel | Cat#: 74095550 |
| Neon Transfection System 100 µL Kit | Thermo Fisher Scientific | Cat#: MPK10096 |
| Human Methylcellulose Base Media | R&D Systems | Cat#: 390394 |

Chemicals used in the study

Table S2.

| **Gene** | **Primer** | **Sequence** |
| --- | --- | --- |
| *ACTB* | F | TGGCATCCACGAAACTACC (19) |
| *ACTB* | R | GTGTTGGCGTACAGGTCTT (19) |
| *GAPDH* | F | CATGAGAAGTATGACAACAG (20) |
| *GAPDH* | R | ATGAGTCCTTCCACGATA (18) |
| *JAK2* | F | TGGGGTTTTCTGGTGCCTTT (20) |
| *JAK2* | R | TAGAGGGTCATACCGGCACA (20) |
| *SIAH1* | F | TCACCAGCAGTTCTTCGCAA (20) |
| *SIAH1* | R | TCGCTTCCCAAGTCAATCGT (20) |
| *SIAH2* | F | CGCCCACAAGAGCATTACCA (20) |
| *SIAH2* | R | CACCAGCATGAAGTGATGGC (20) |
| *UBE2L6* | F | AGACCGAATATCAGGGAGCC (20) |
| *UBE2L6* | R | GGGTGAACTCTTCGGCATTC (20) |

Primers used for qRT-PCR

Table S3.

| **Gene** | **Primer** | **Sequence** |
| --- | --- | --- |
| *Mouse Jak2* | F | CGGATTAACCAAAGGCTTGCCGCAGGAC (28) |
| *Mouse Jak2* | R | GTCCTGCGGCAAGCCTTTGGTTAATCCG (28) |

Primers used for site-directed mutagenesis
