# Supplementary figures and images for: The deacetylases HDAC1/HDAC2 control JAK2V617F-STAT signaling through the ubiquitin ligase SIAH2
Source: Signal Transduct Target Ther. 2025 Aug 29;10:275. doi: 10.1038/s41392-025-02369-7 (PMC12394589; doi:10.1038/s41392-025-02369-7)

**(Original immunoblots)**


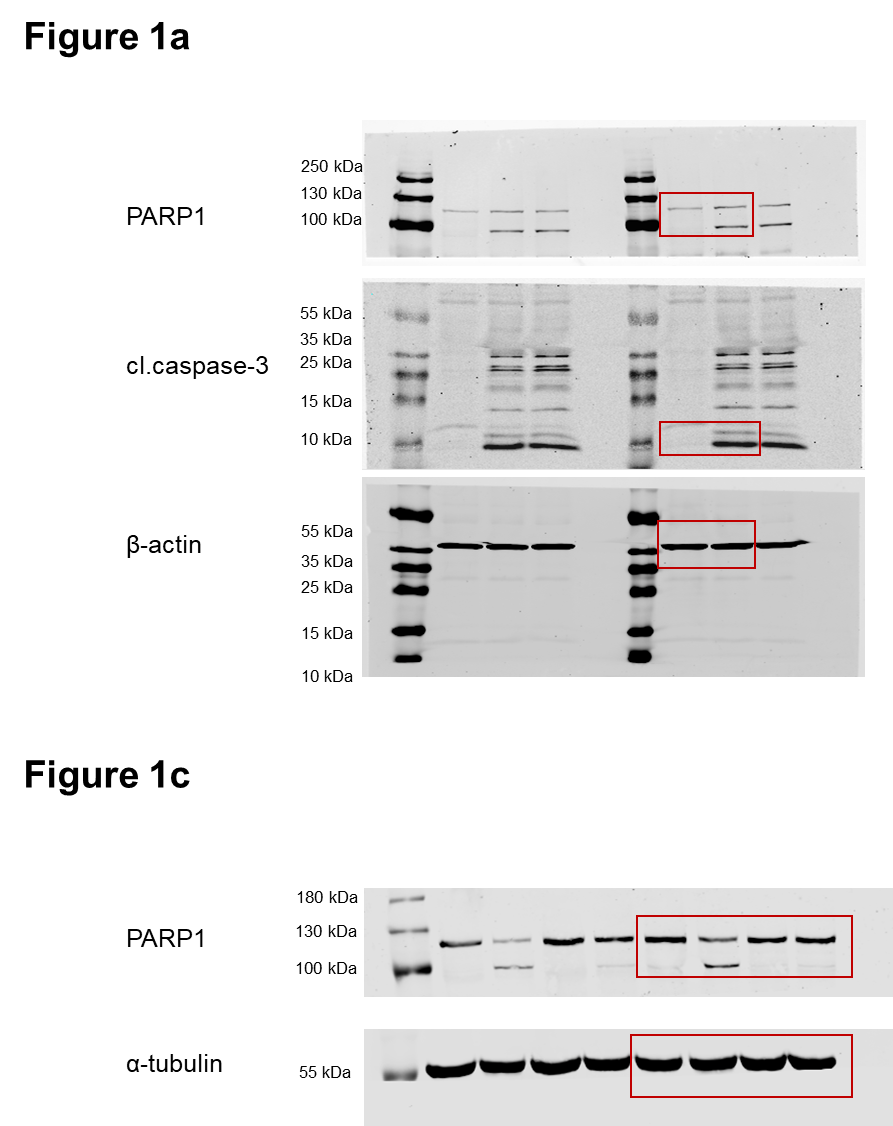


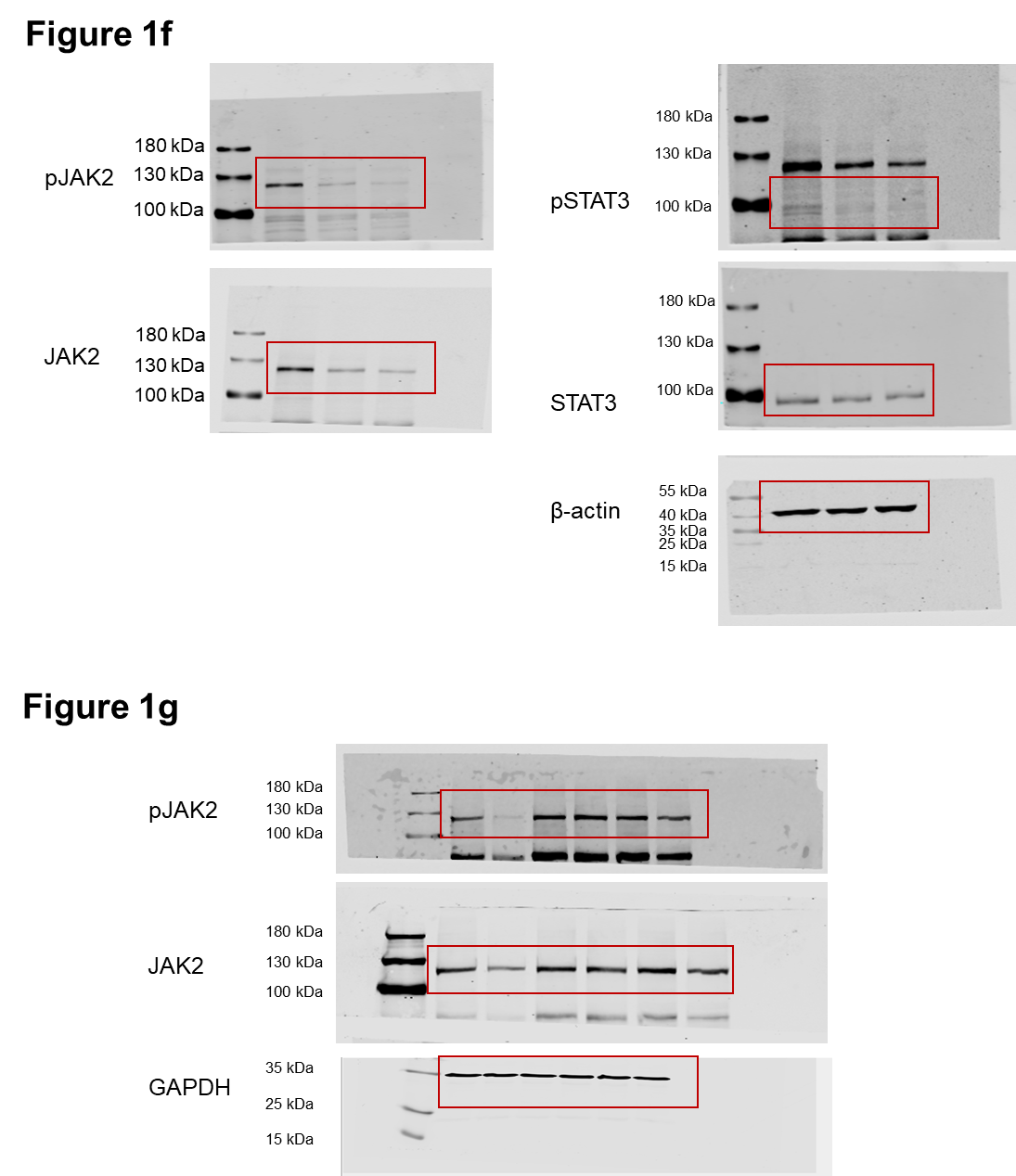


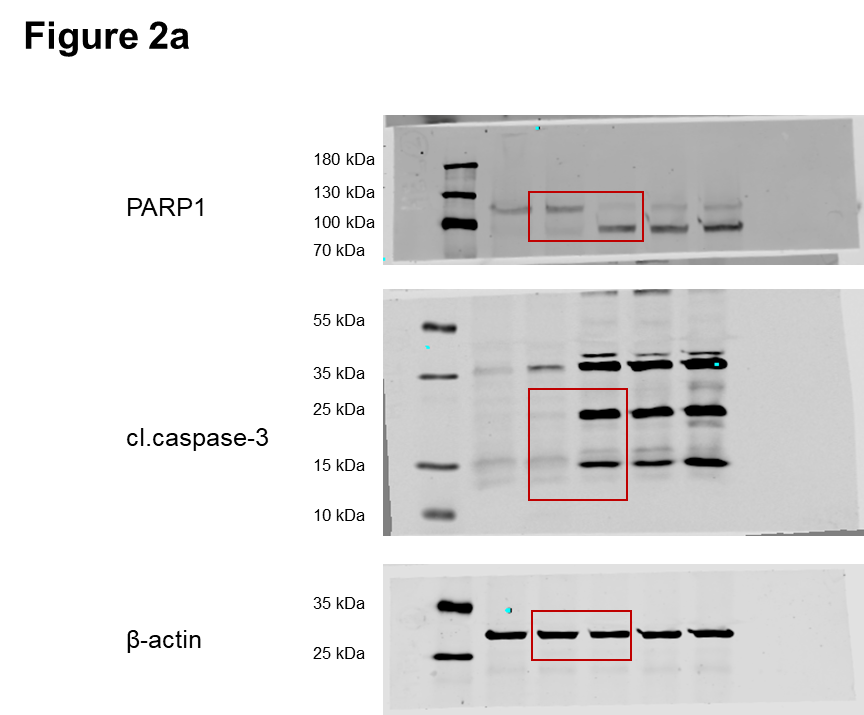


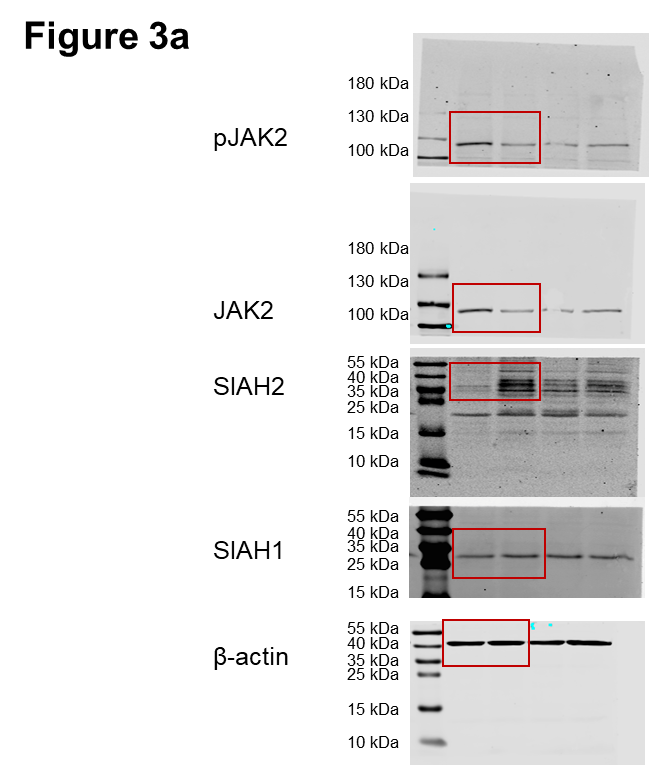


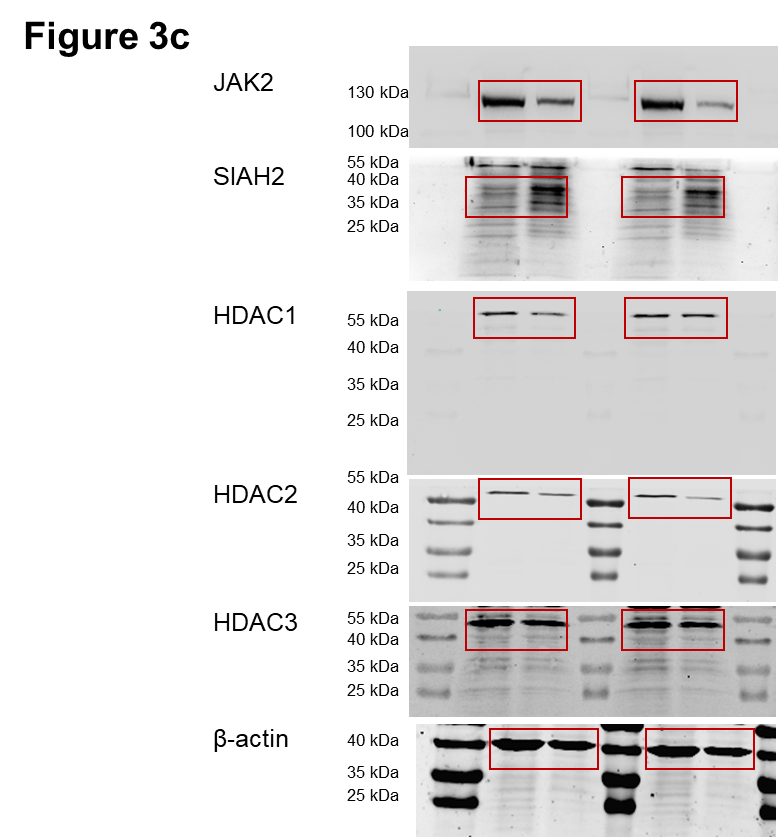


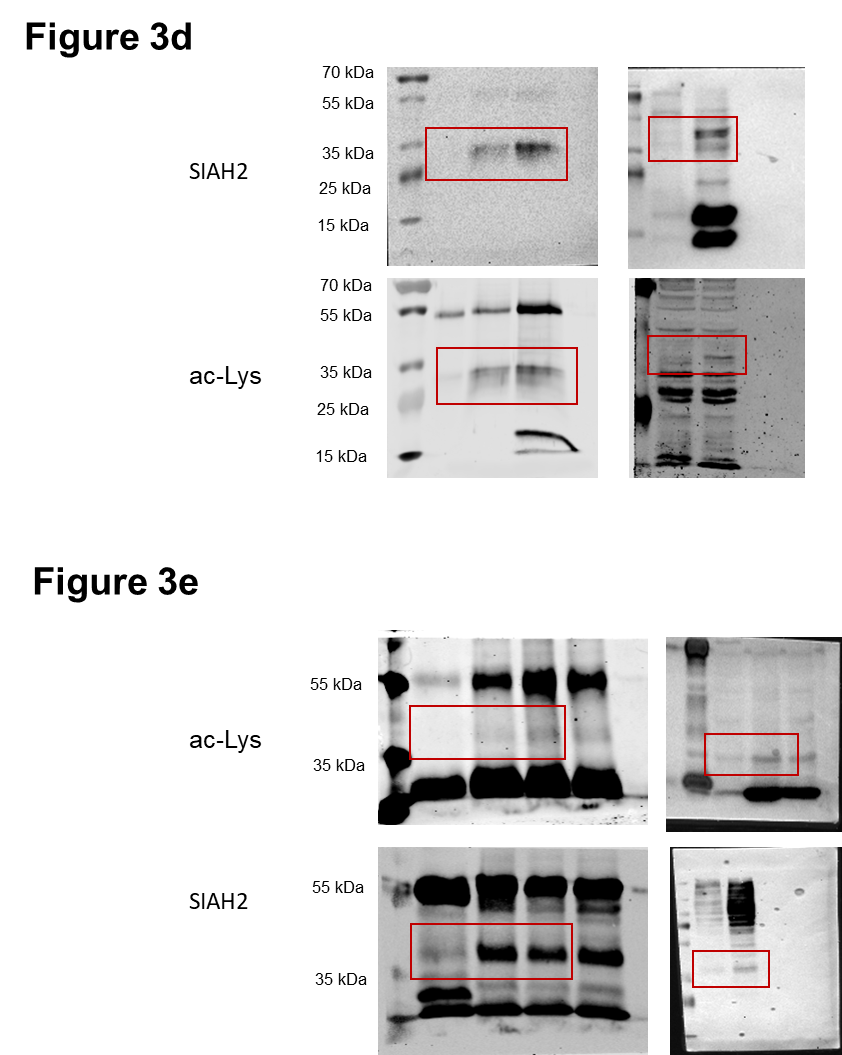


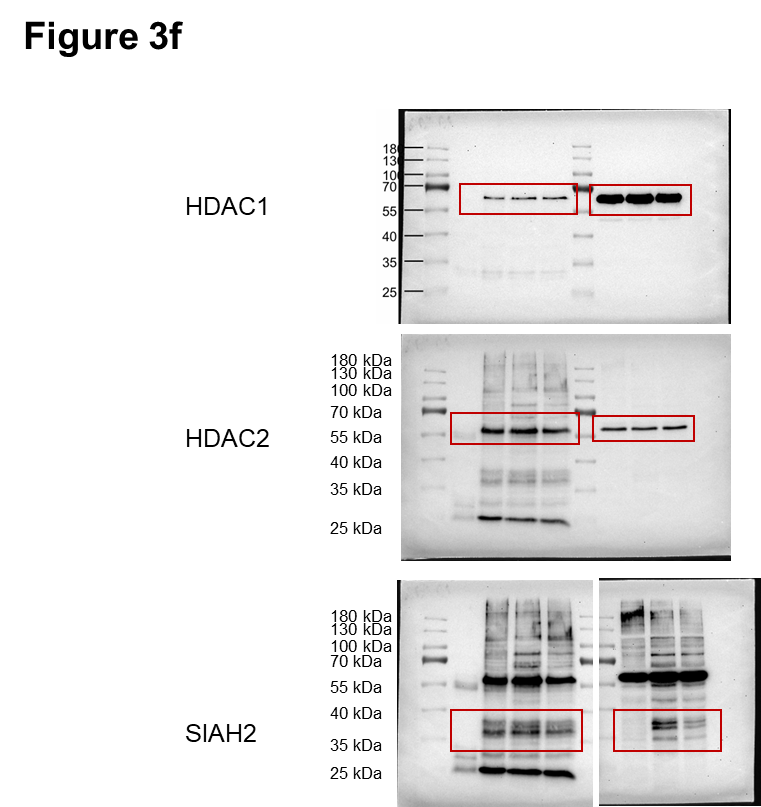


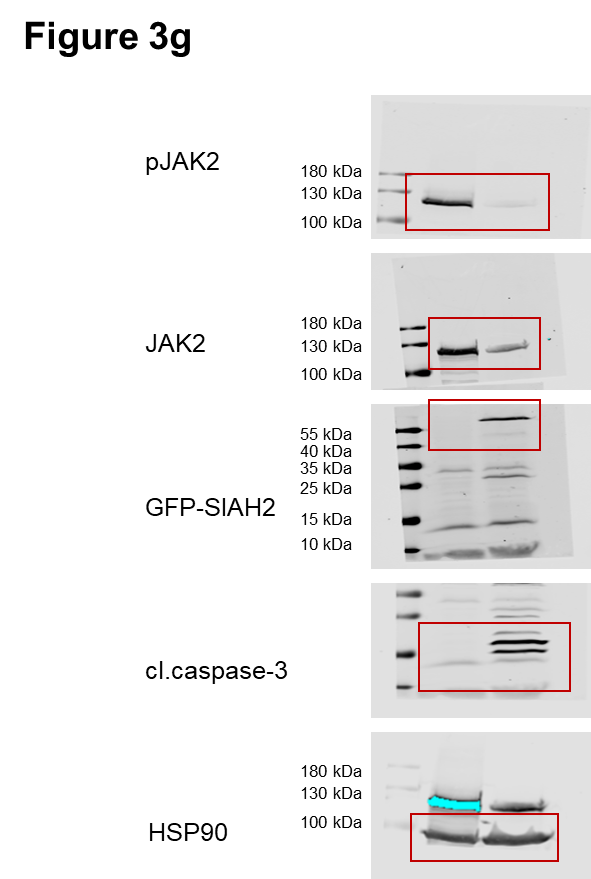


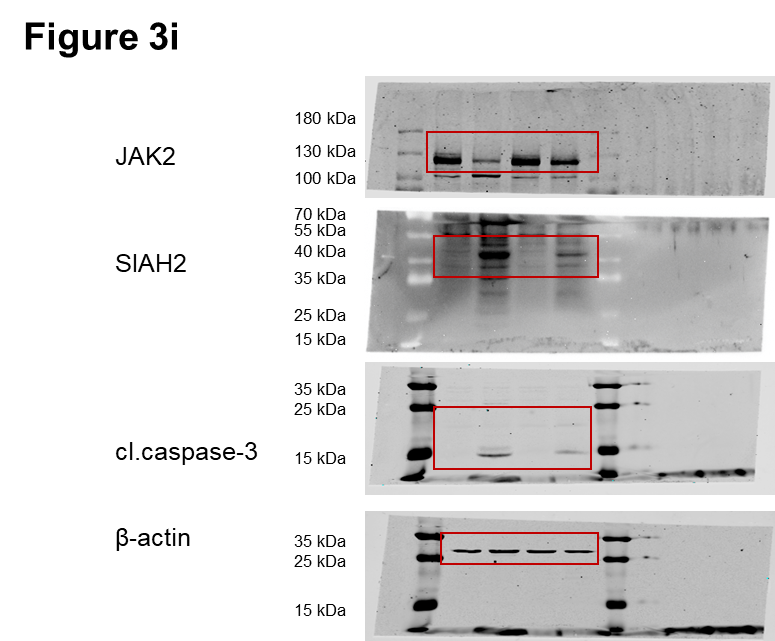


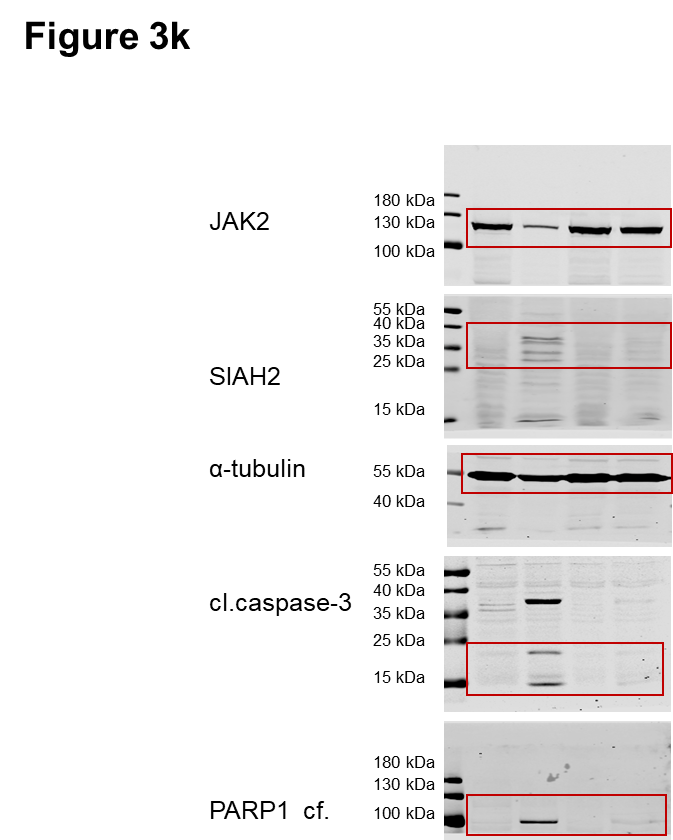


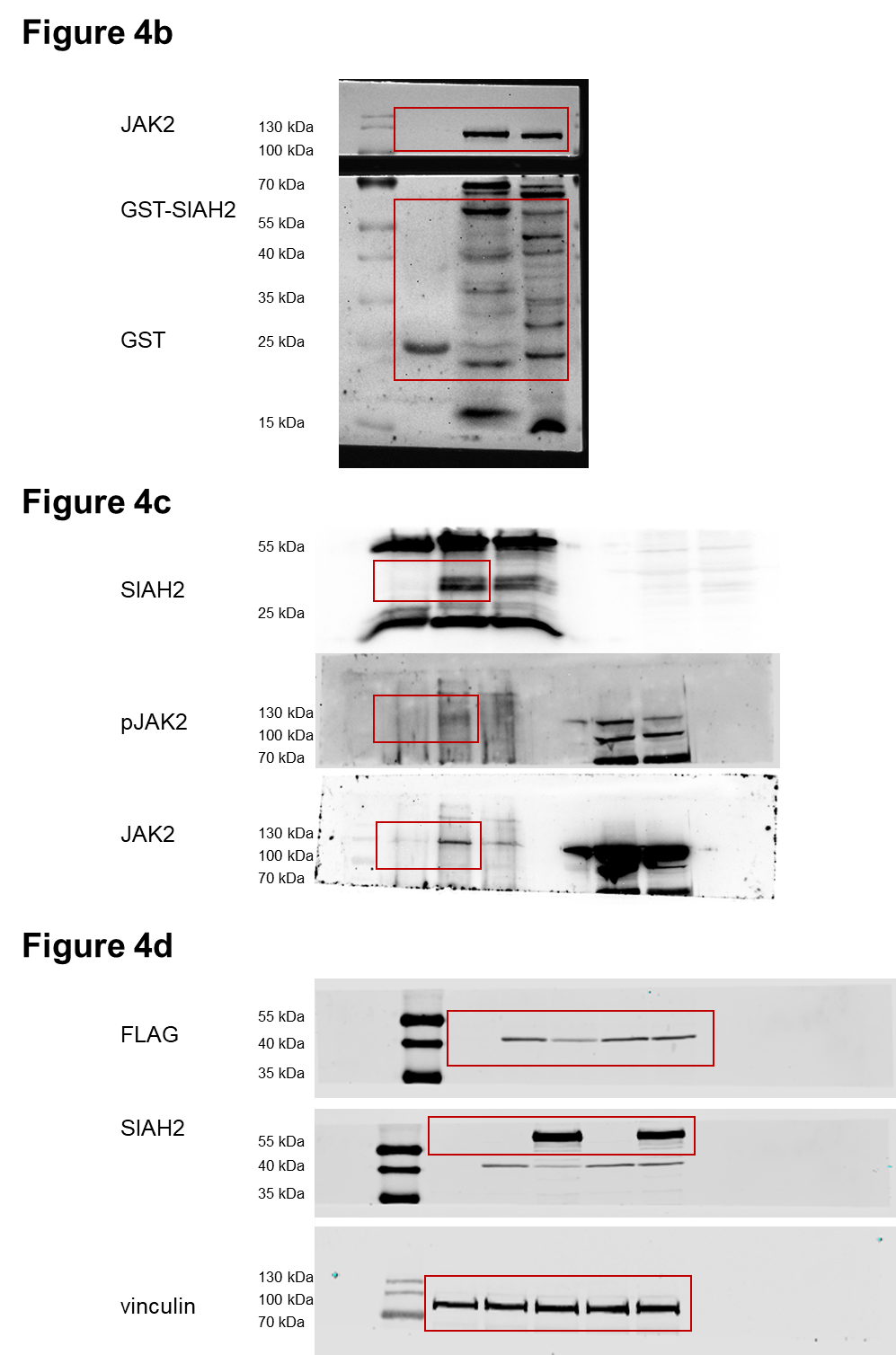


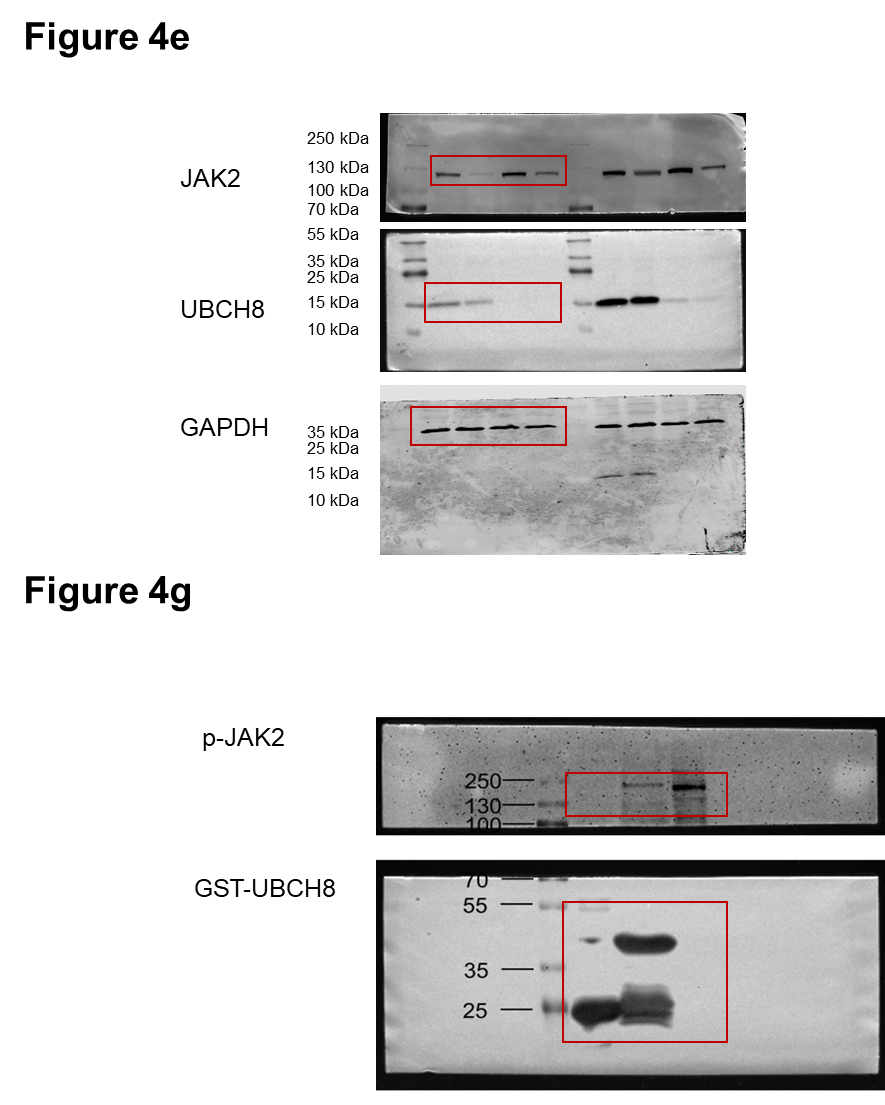


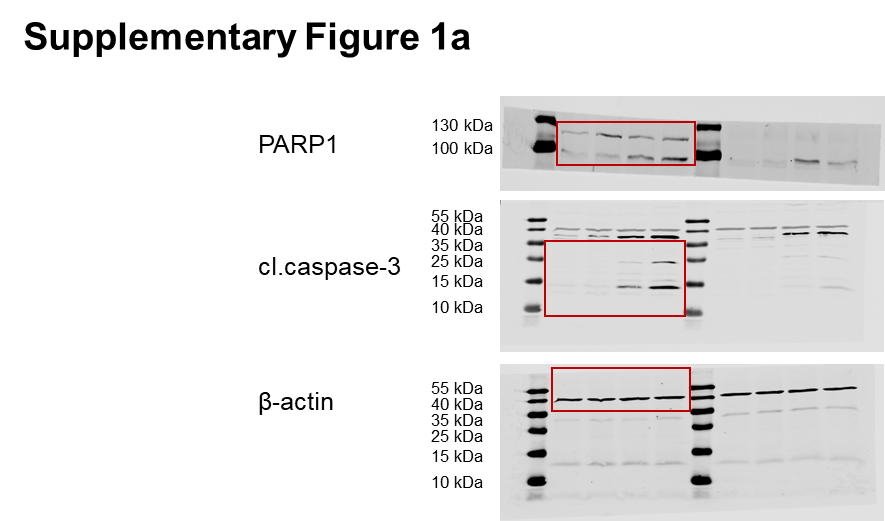


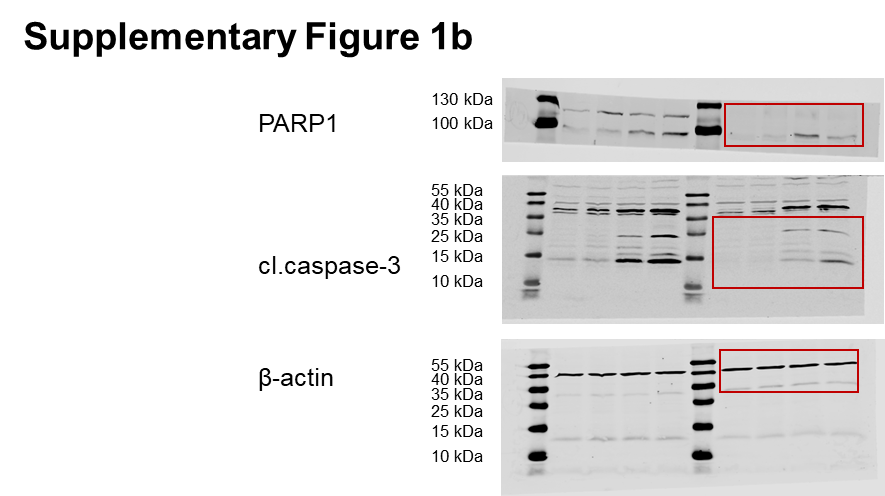


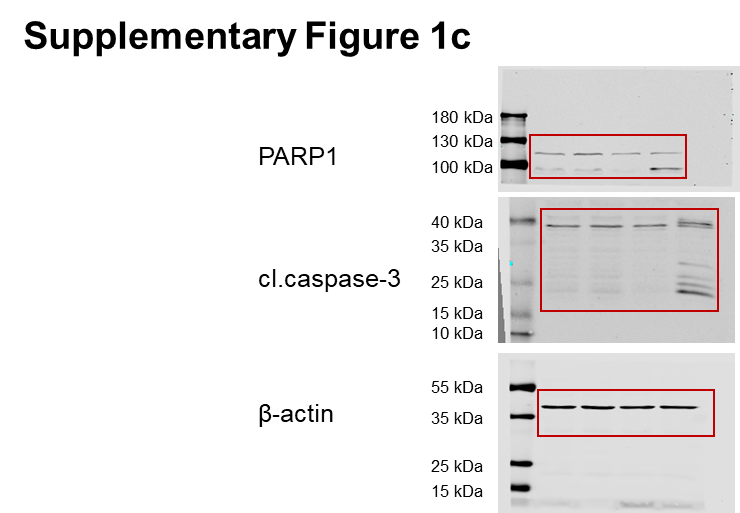


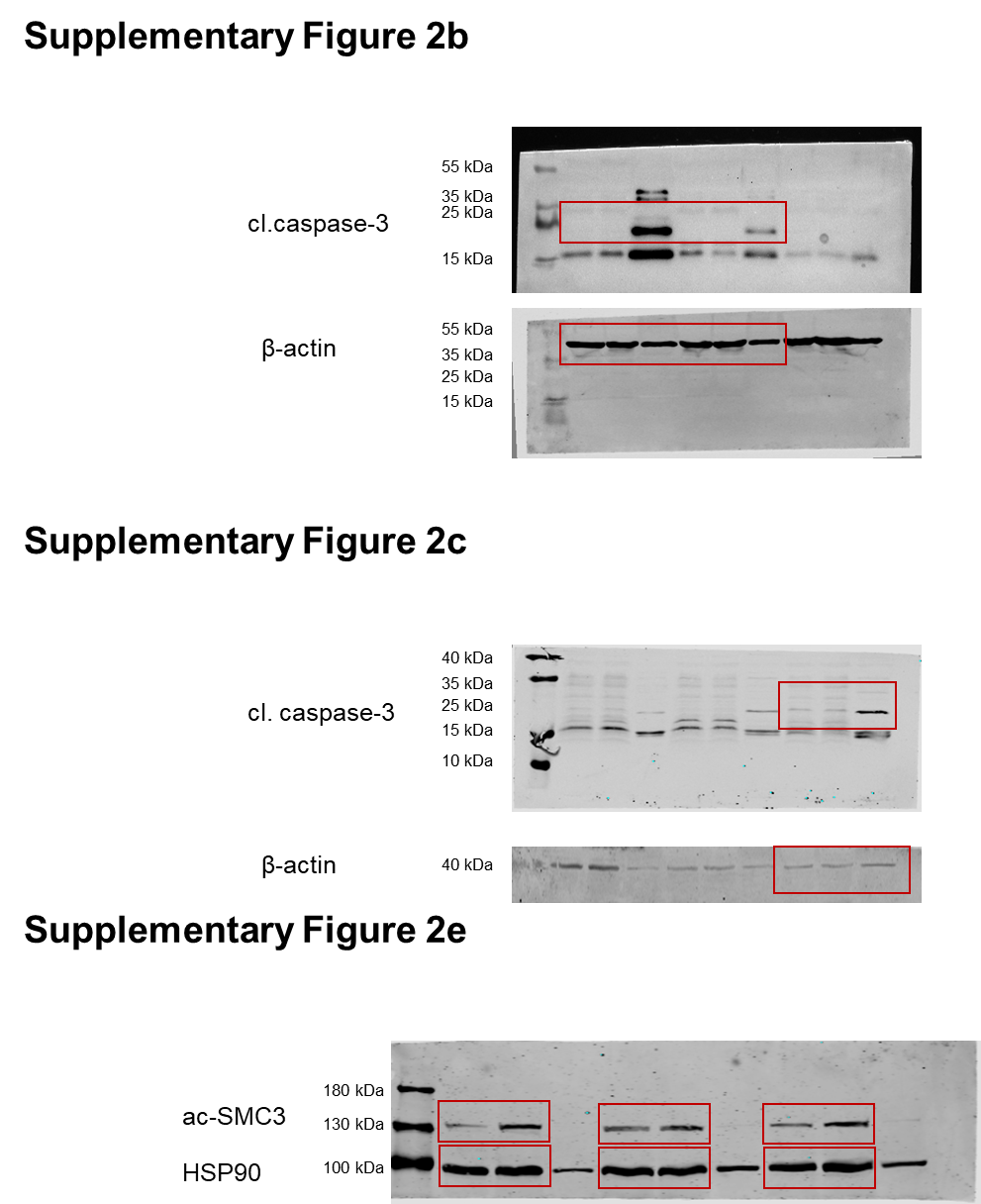


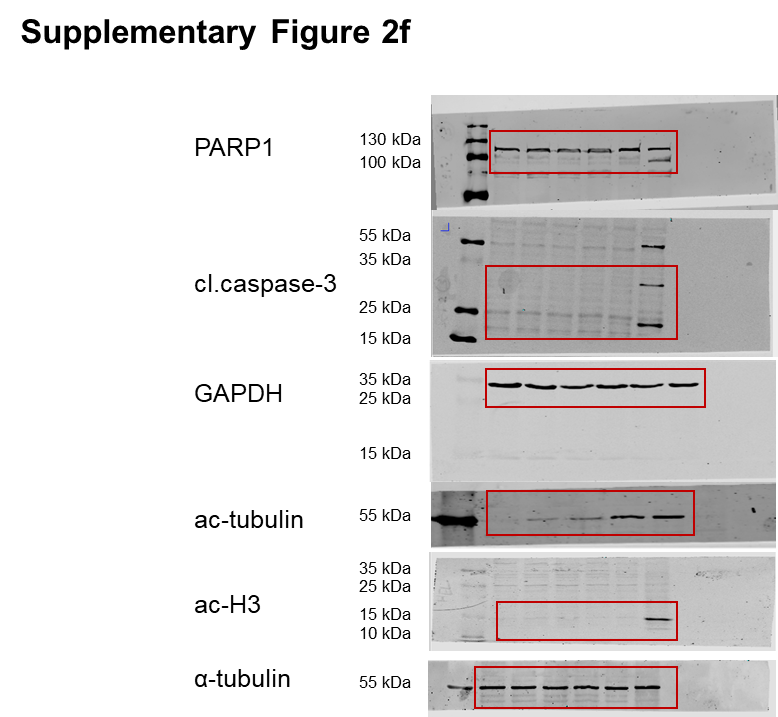


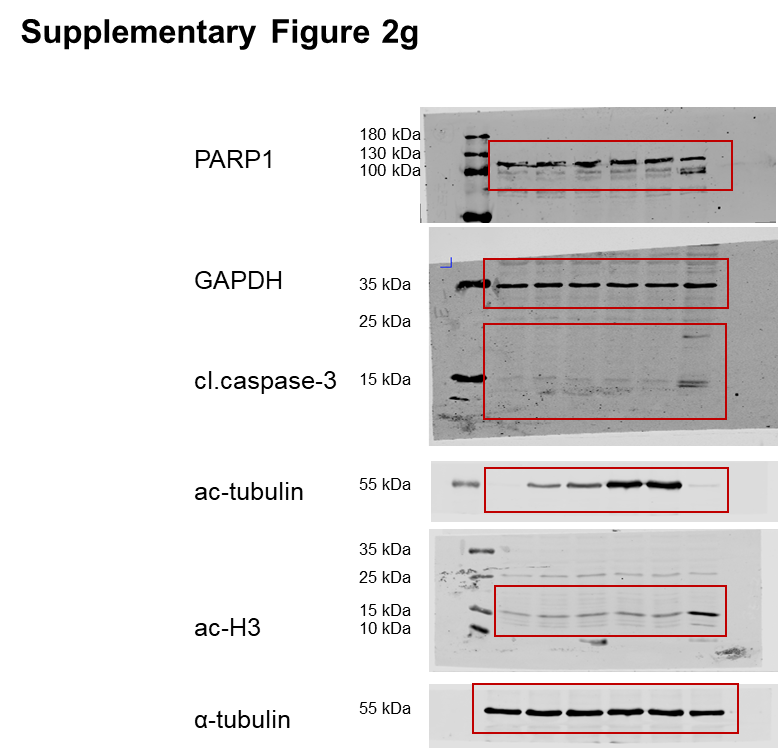


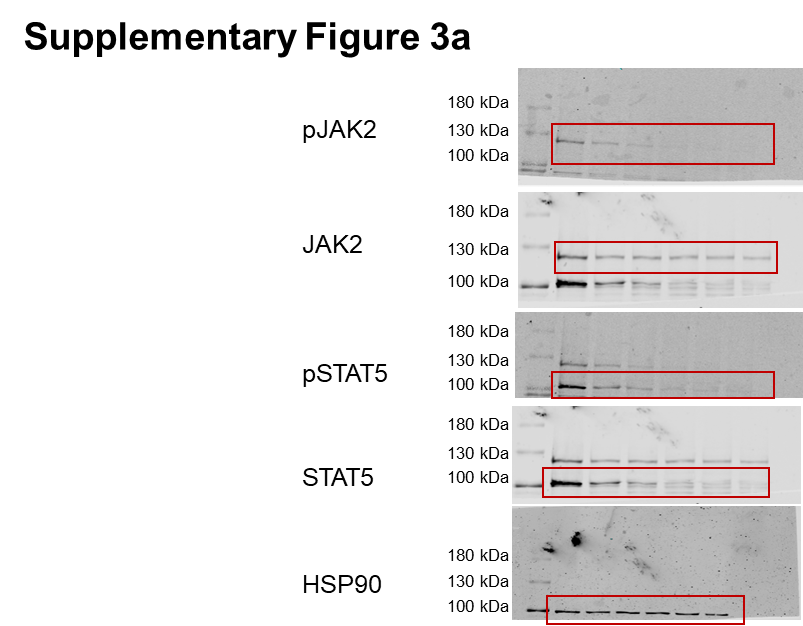


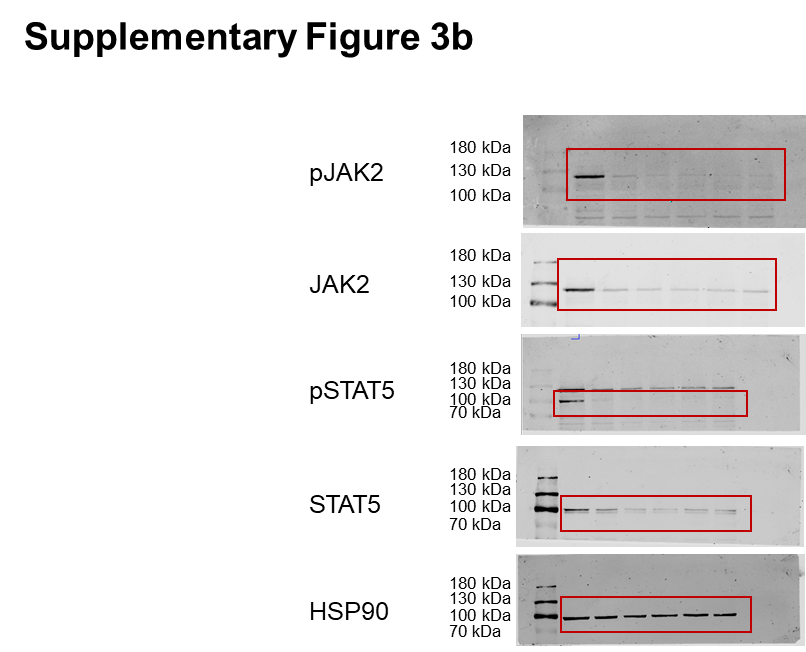


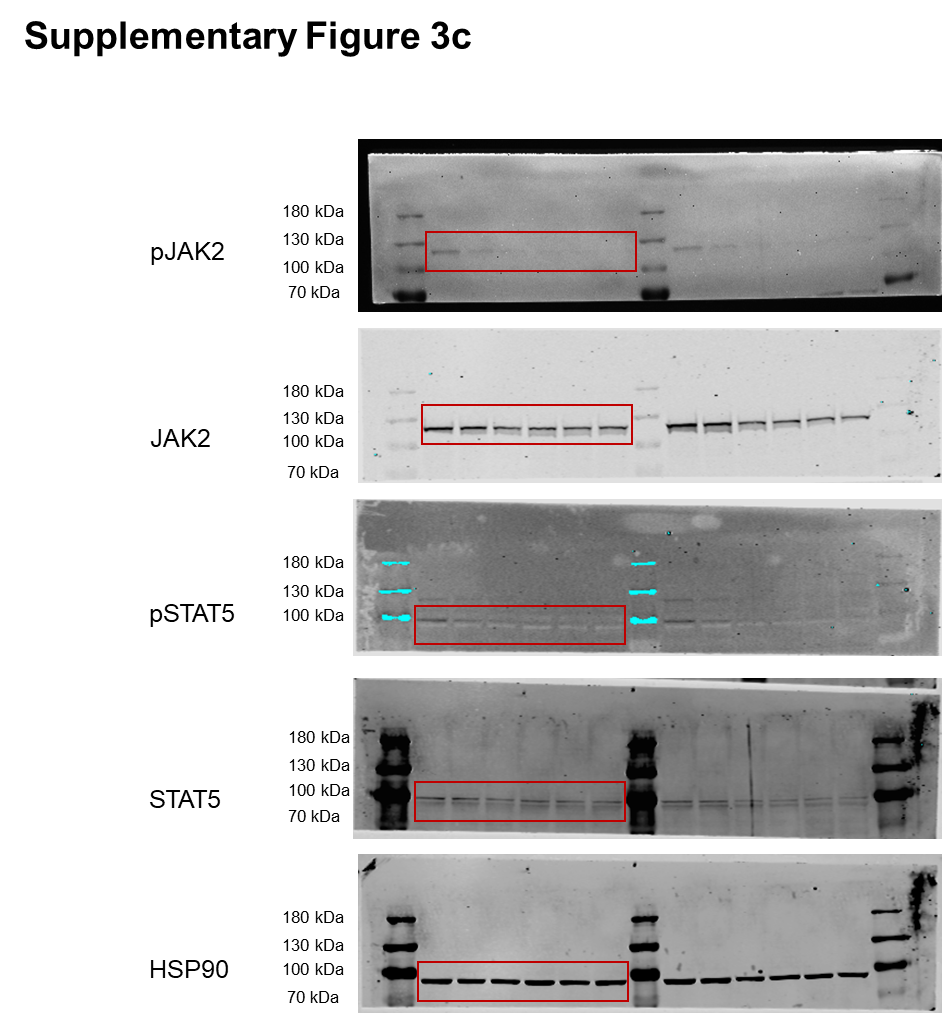


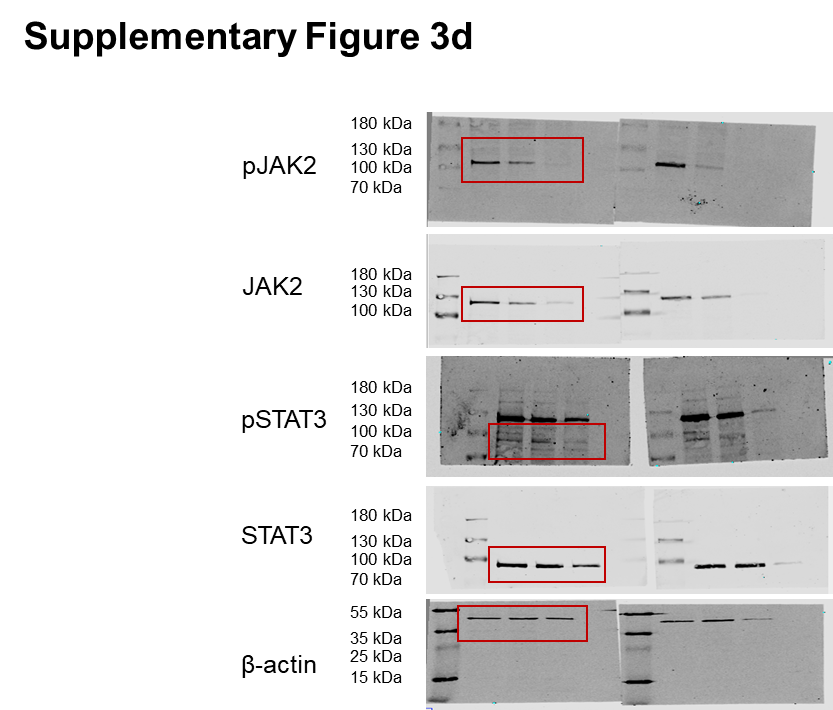


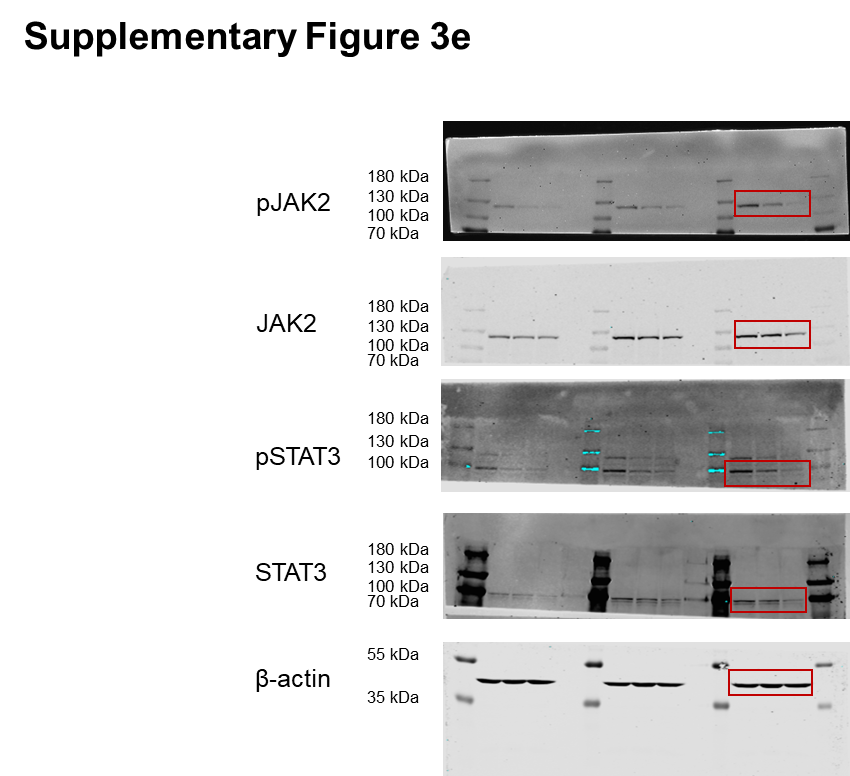


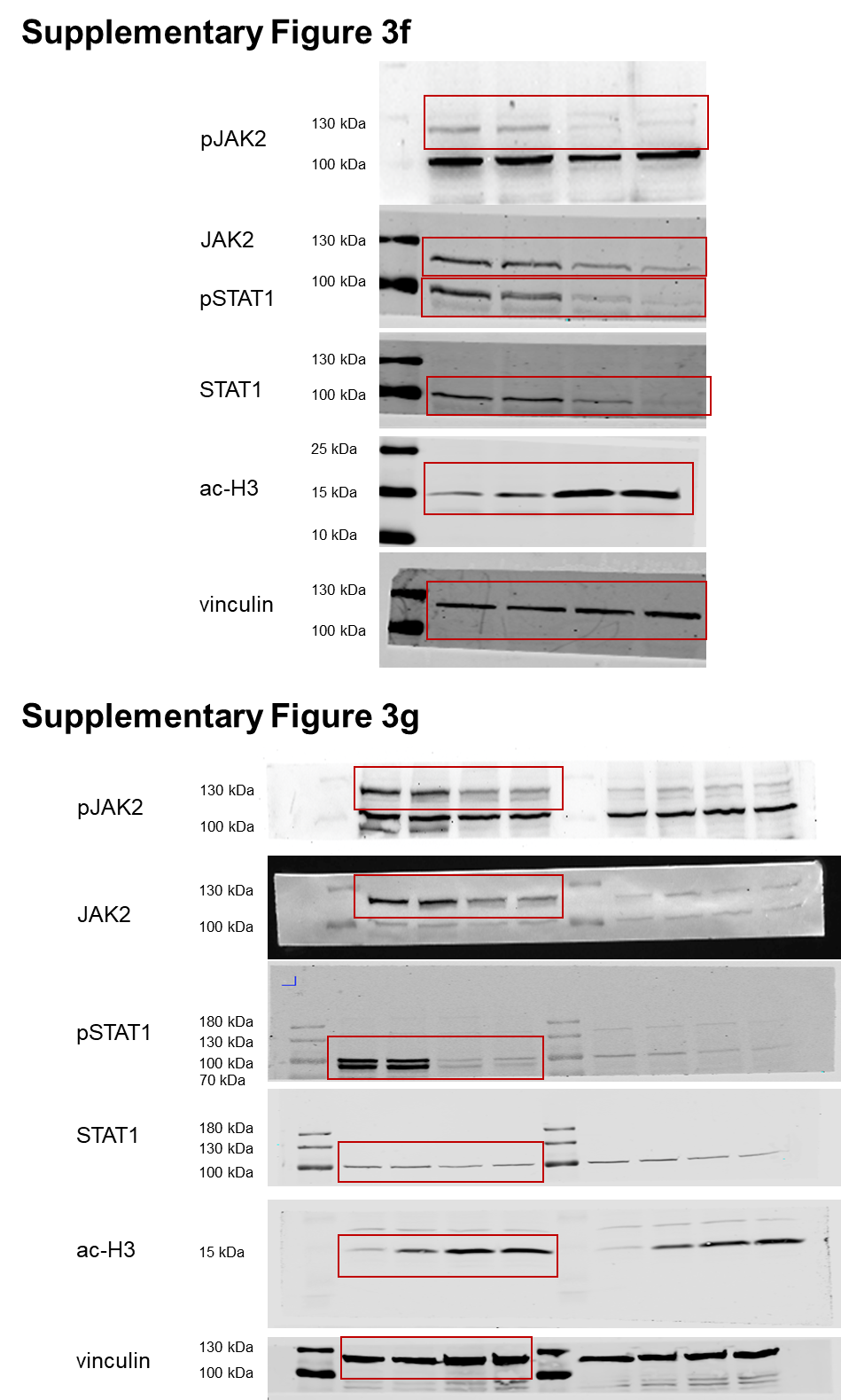


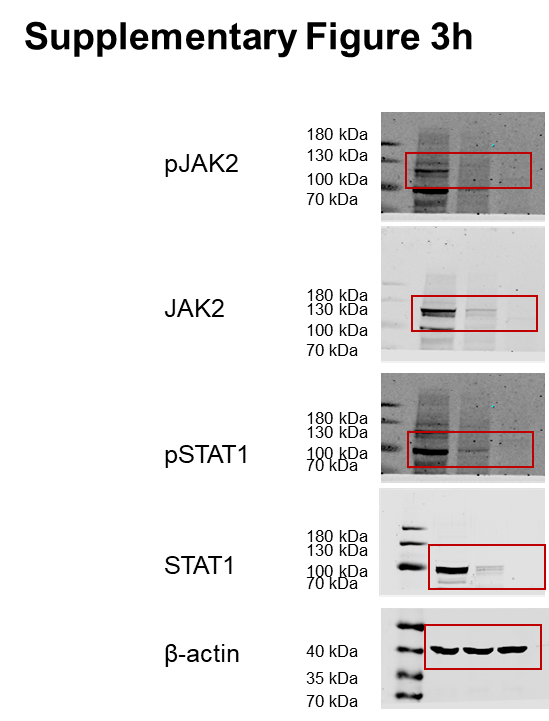


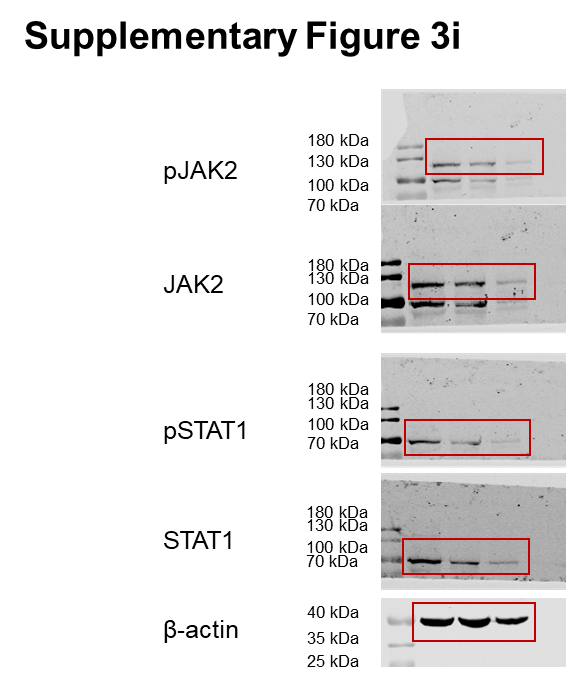


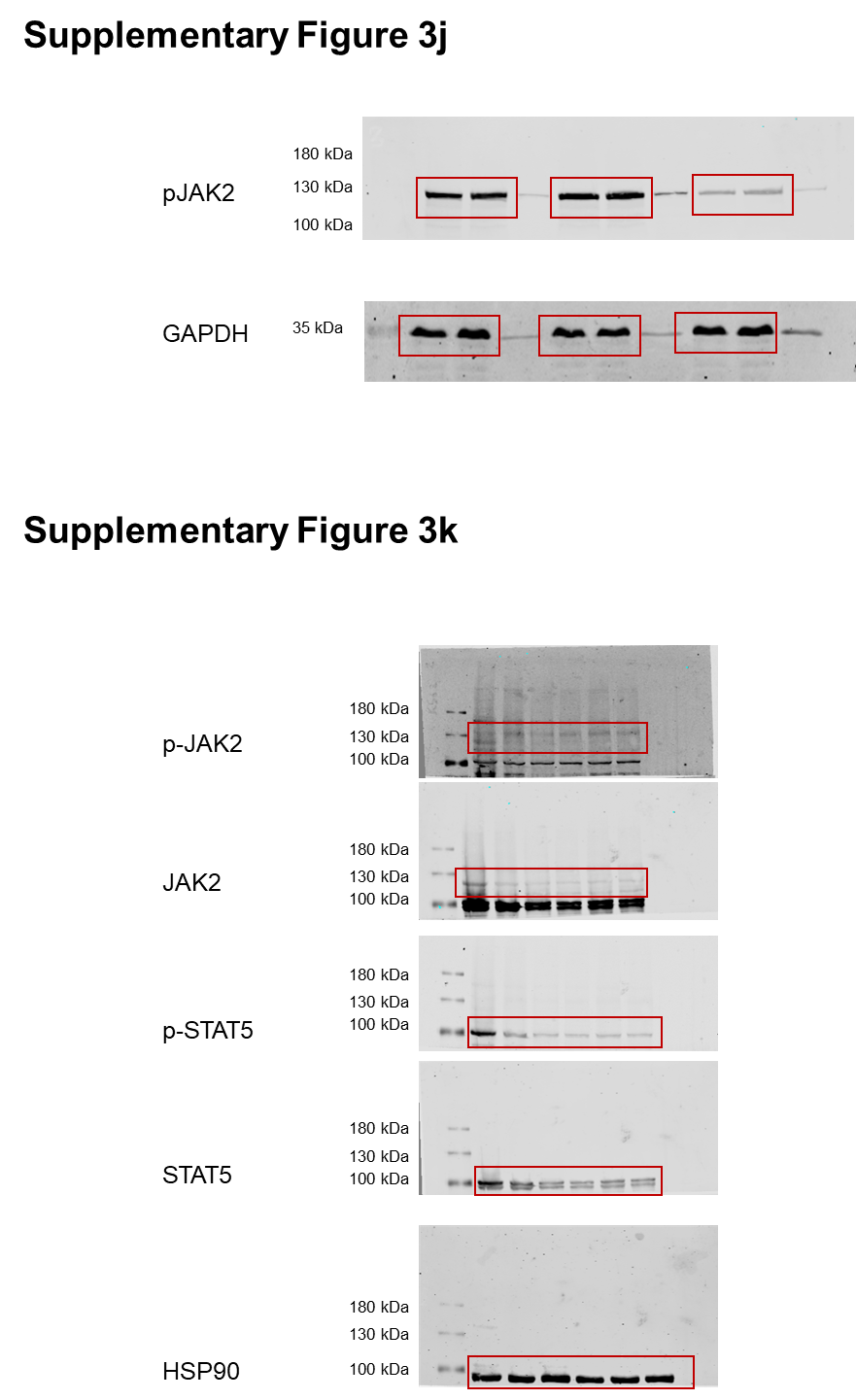


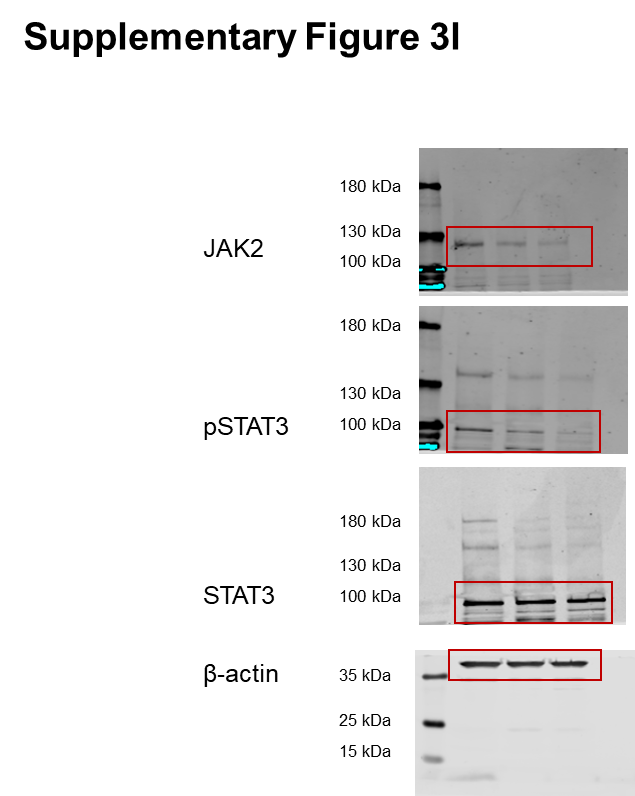


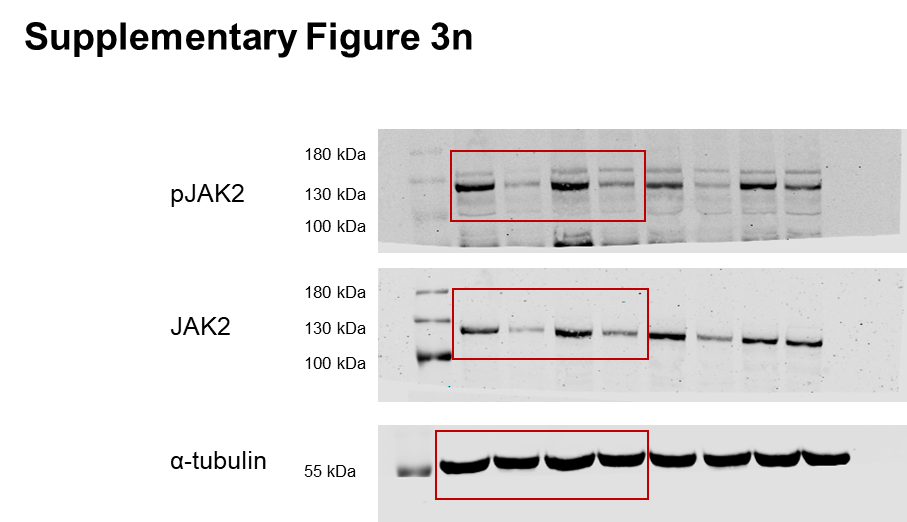


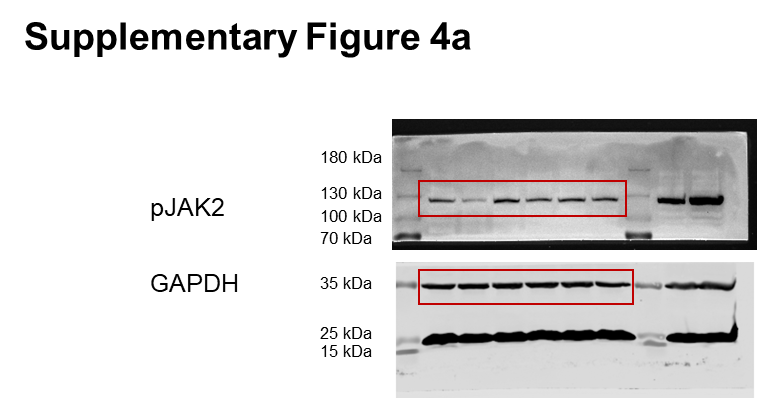


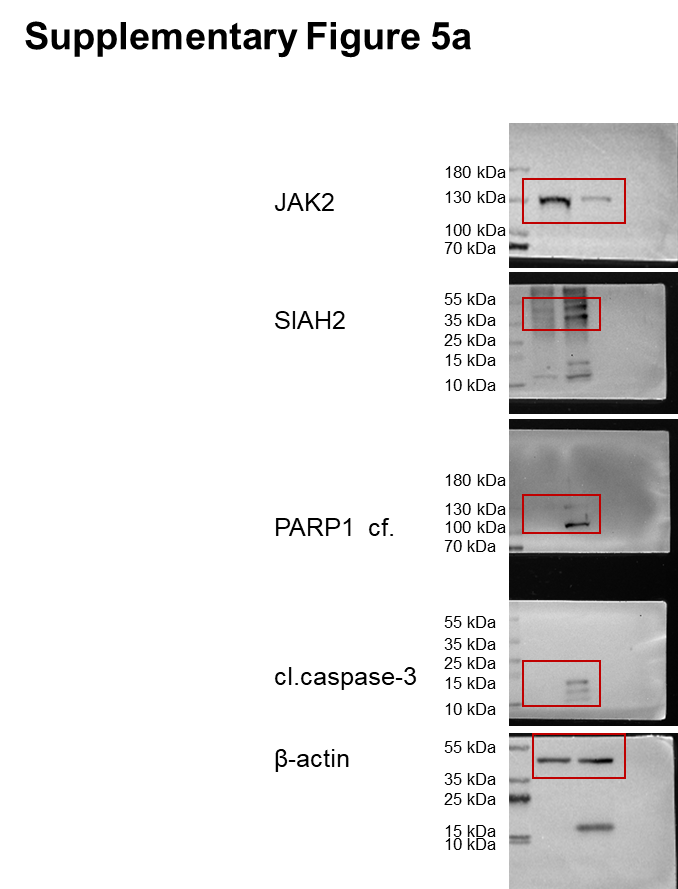


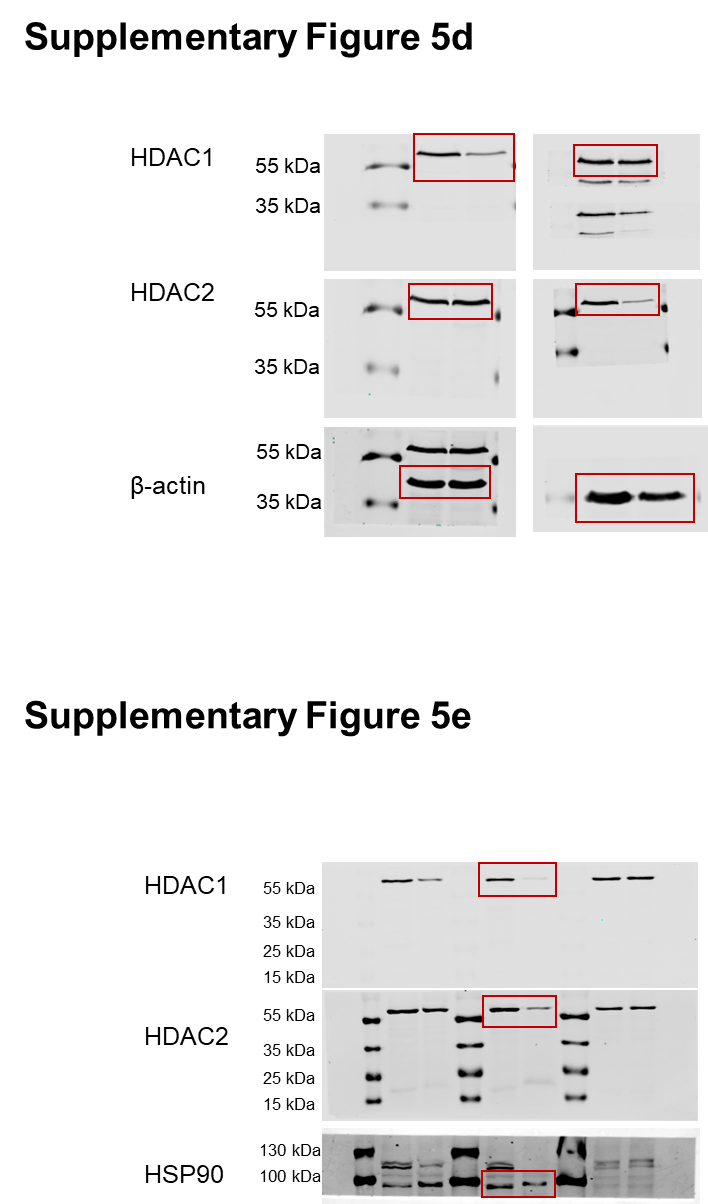


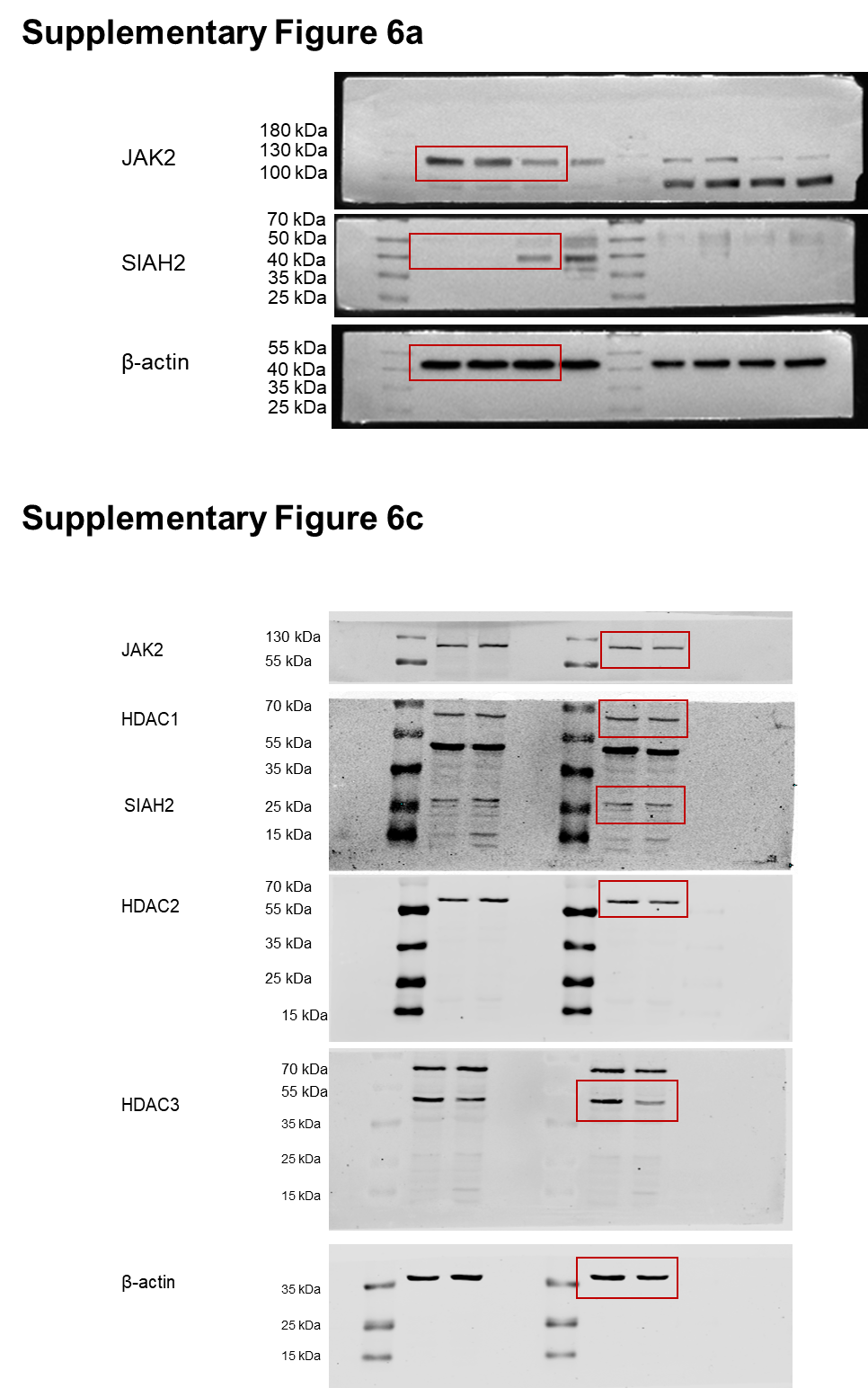


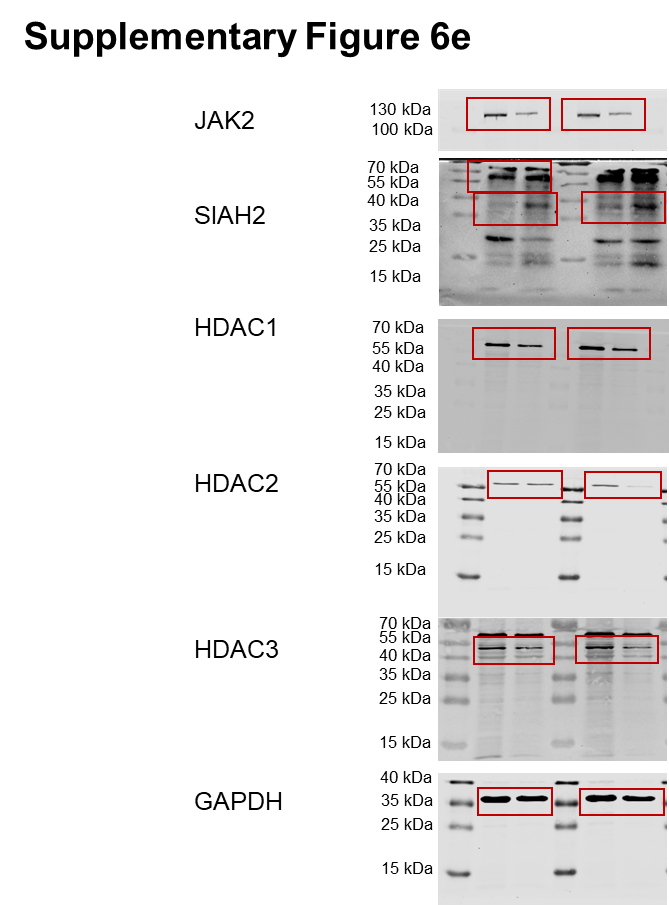


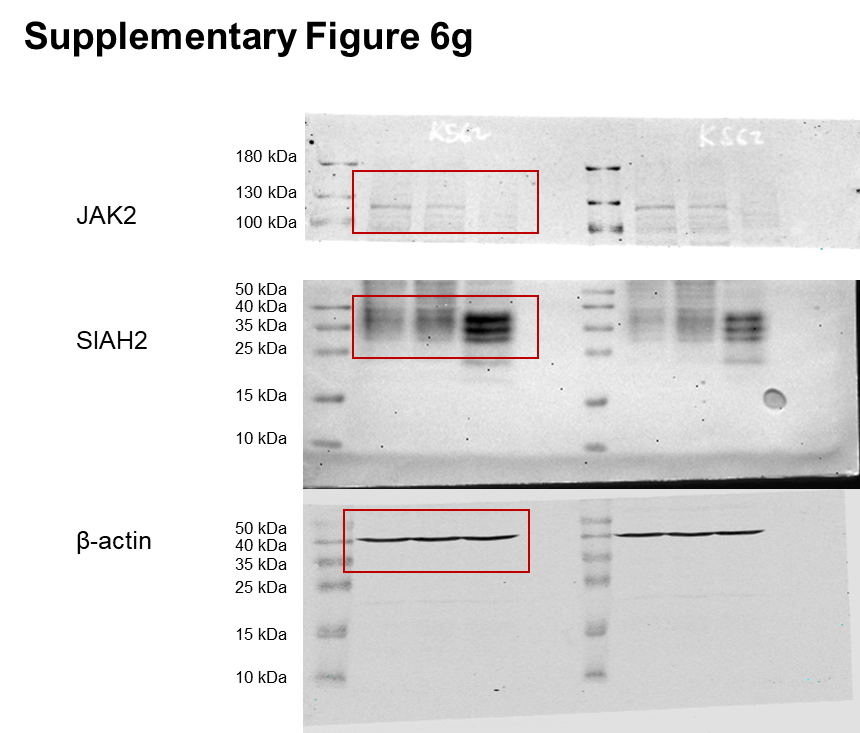


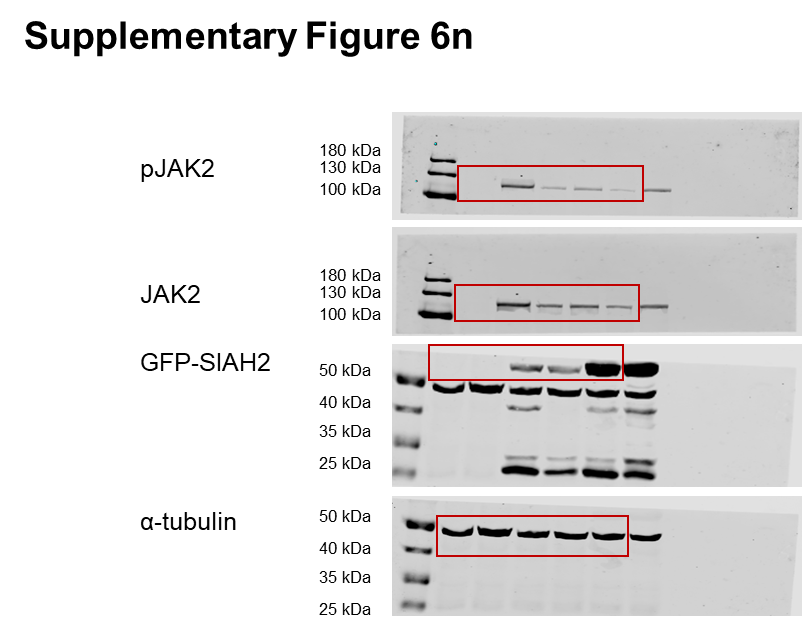


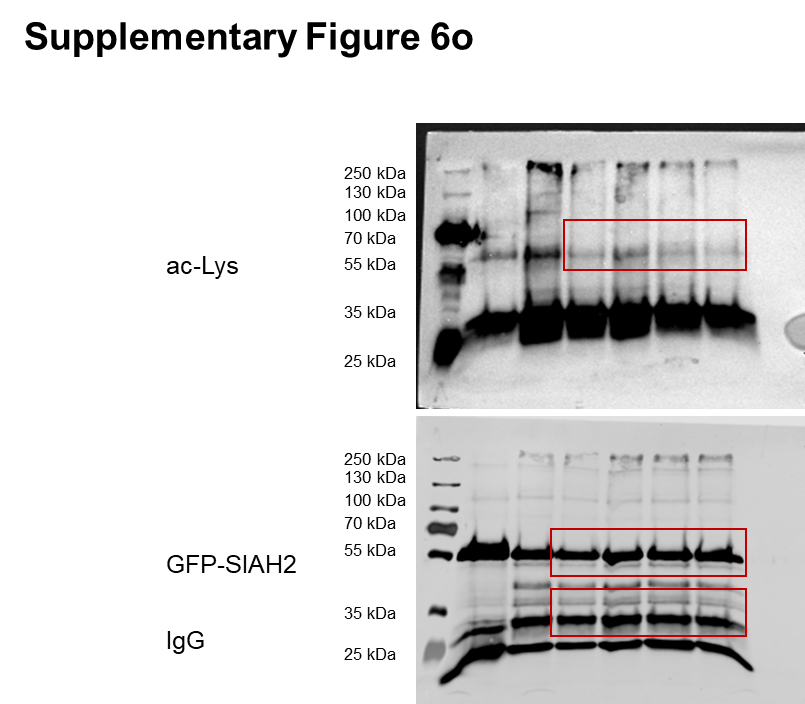


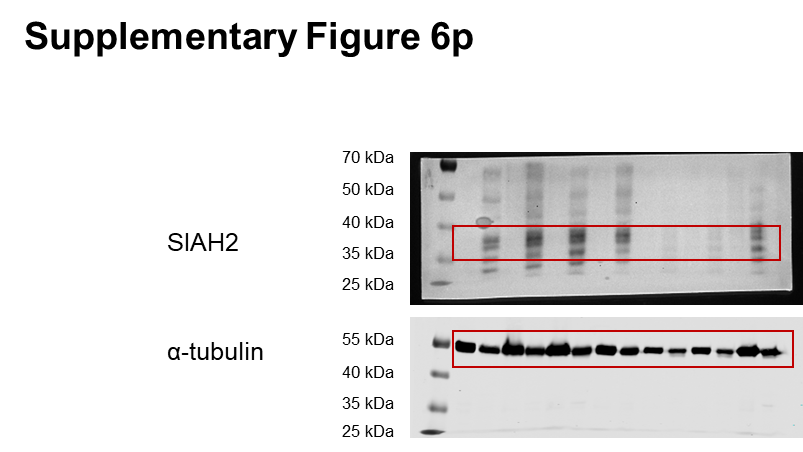


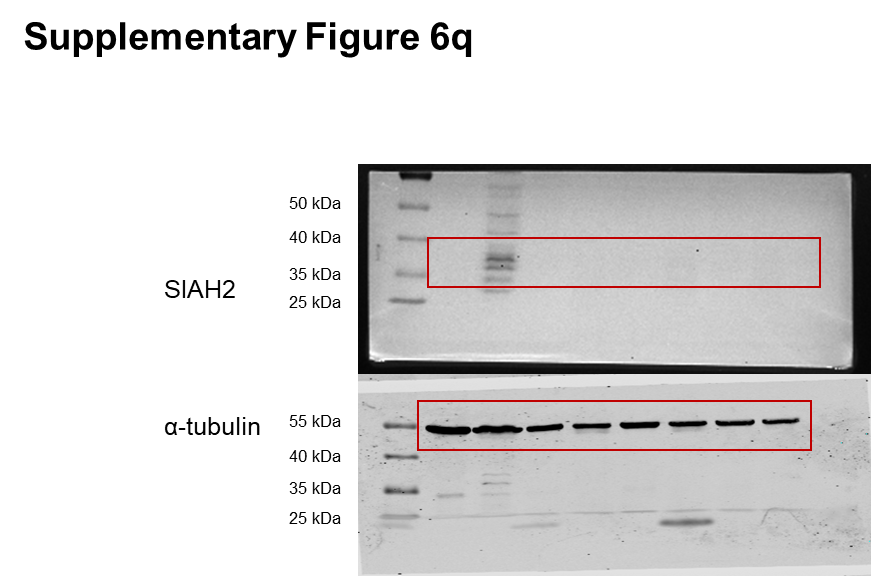


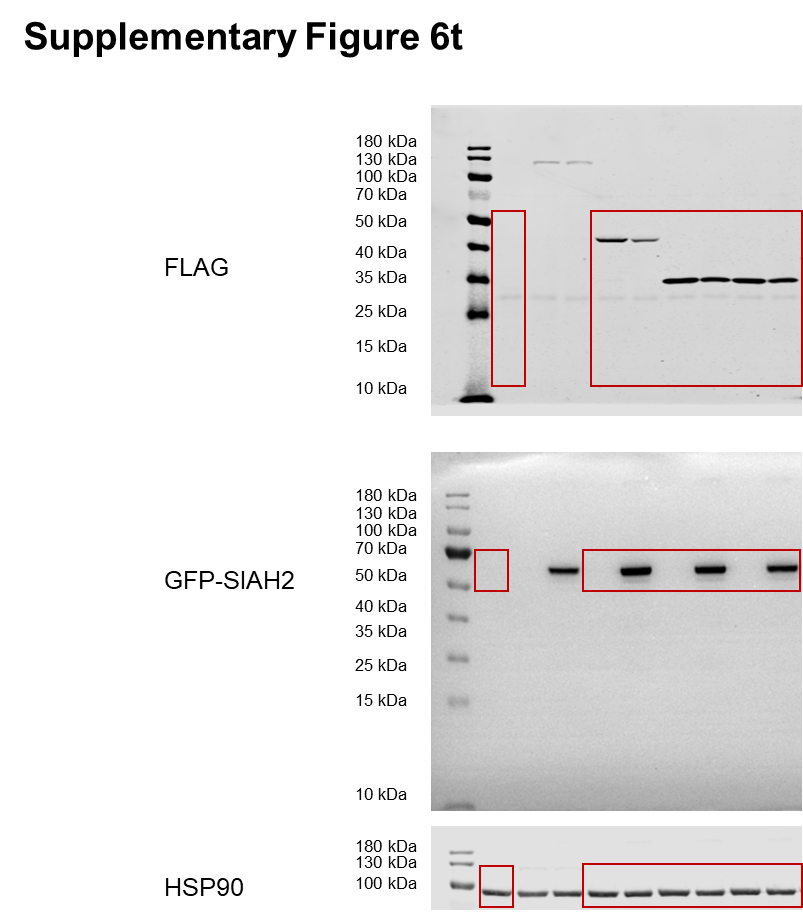


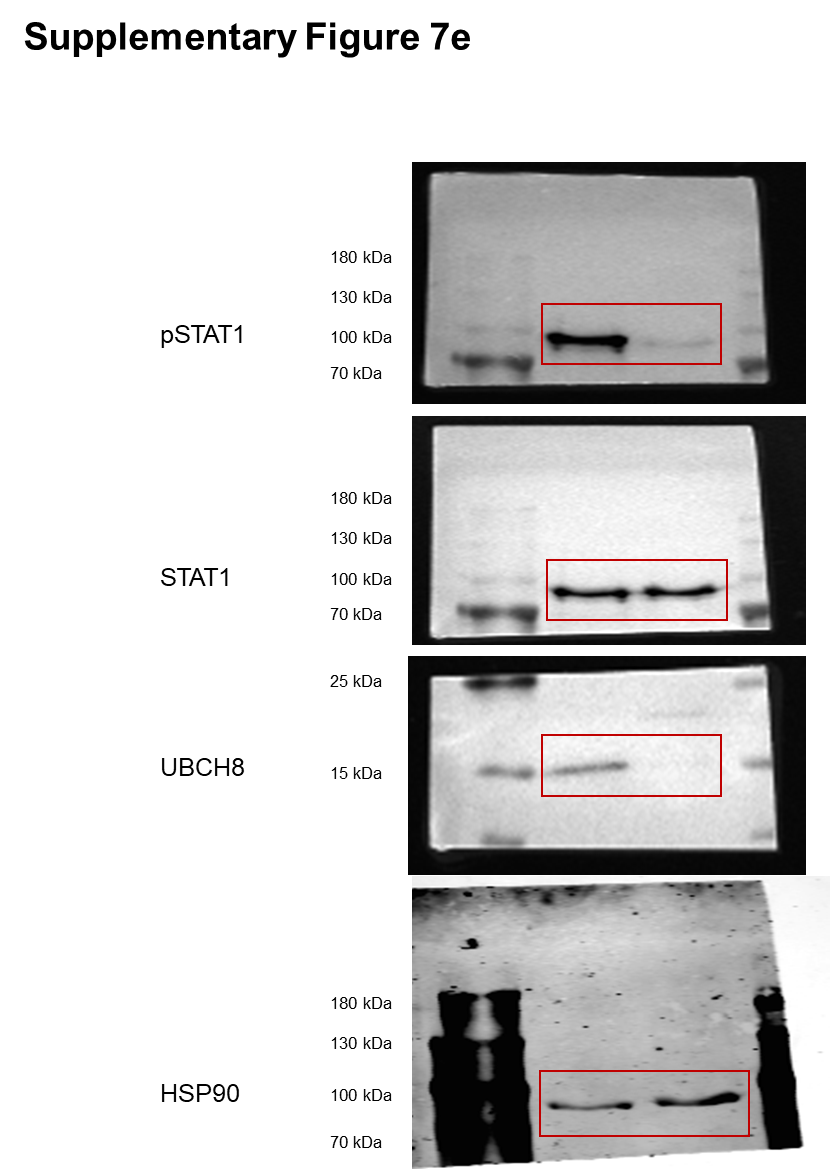


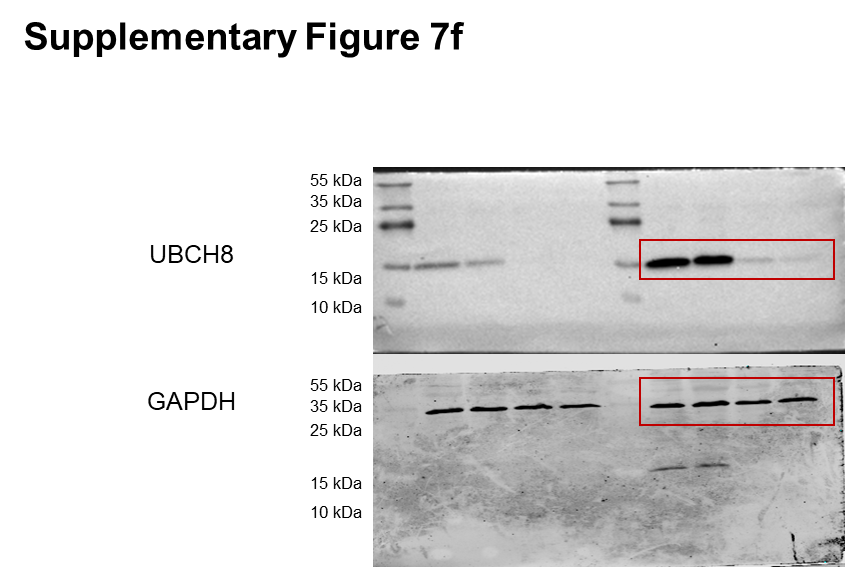

Supplement: Supplementary file 2 — Original Immunoblots [file 41392_2025_2369_MOESM2_ESM.docx]
